# Marine habitat use and feeding ecology of introduced anadromous brown trout at the colonization front of the sub-Antarctic Kerguelen archipelago

**DOI:** 10.1038/s41598-021-91405-x

**Published:** 2021-06-07

**Authors:** Jan Grimsrud Davidsen, Xavier Bordeleau, Sindre Håvarstein Eldøy, Frederick Whoriskey, Michael Power, Glenn T. Crossin, Colin Buhariwalla, Philippe Gaudin

**Affiliations:** 1 NTNU University Museum, Norwegian University of Science and Technology, Trondheim, Norway; 2 Department of Biology, Dalhousie University, Halifax, NS Canada; 3 Department of Fisheries and Oceans Canada, Maurice Lamontagne Institute, Mont-Joli, QC G5H 3Z4 Canada; 4 Ocean Tracking Network, Dalhousie University, 1355 Oxford St., Halifax, NS B3H 4R2 Canada; 5 Department of Biology, University of Waterloo, Waterloo, ON N2L 3G1 Canada; 6 Nova Scotia Department of Fisheries and Aquaculture, Pictou, NS Canada; 7 Université de Pau et des Pays de l’Adour, e2s UPPA, INRAE, ECOBIOP, Aquapôle INRAE, Saint-Pée-sur-Nivelle, France

**Keywords:** Ecology, Animal migration, Behavioural ecology, Stable isotope analysis

## Abstract

In 1954, brown trout were introduced to the Kerguelen archipelago (49°S, 70°E), a pristine, sub-Antarctic environment previously devoid of native freshwater fishes. Trout began spreading rapidly via coastal waters to colonize adjacent watersheds, however, recent and unexpectedly the spread has slowed. To better understand the ecology of the brown trout here, and why their expansion has slowed, we documented the marine habitat use, foraging ecology, and environmental conditions experienced over one year by 50 acoustically tagged individuals at the colonization front. Trout mainly utilized the marine habitat proximate to their tagging site, ranging no further than 7 km and not entering any uncolonized watersheds. Nutritional indicators showed that trout were in good condition at the time of tagging. Stomach contents and isotope signatures in muscle of additional trout revealed a diet of amphipods (68%), fish (23%), isopods (6%), and zooplankton (6%). The small migration distances observed, presence of suitable habitat, and rich local foraging opportunities suggest that trout can achieve their resource needs close to their home rivers. This may explain why the expansion of brown trout at Kerguelen has slowed.

## Introduction

The introduction of exotic species into novel landscapes, both deliberately and unintentionally, is a major biodiversity and ecological concern^1–3^. Historically, species introductions were often done to provide economic benefits to humans^4,5^, by establishing self-sustaining populations of plants and animals for harvest. The success of introductions is influenced by a broad array of environmental (physical and biological) factors as well as the life history characteristics and behaviour of the introduced species^6^, and these may act independently or in concert. In nature, many of these variables are beyond the control of investigators attempting to understand the characteristics of species that determine colonization success and/or their impacts on ecosystems. This makes it very difficult to predict the success or failure of species introductions.

Nevertheless, well-documented introductions can provide useful insights into the ecology and evolution of fishes, as well as the factors influencing their colonization success, while also providing valuable information for conservation-based restoration programs^7^. Recent studies have begun to describe the introductions and expansion of salmonid fishes (Salmonidae) into the previously fishless^8^ freshwater ecosystems of sub-Antarctic islands, including Kerguelen, Crozet, and Marion^9–15^. At Kerguelen, in particular, extensive monitoring and documentation of introduced brown trout (*Salmo trutta*) populations has chronicled the pattern of spread of the species, and provided hypotheses about the factors driving and determining colonization success^11^.

Freshwater systems located within the sub-Antarctic islands are generally nutrient limited e.g.,^16,17^ making it difficult to sustain fish populations. Introduced salmonids therefore have a simple and restricted diet of allochthonous and aquatic prey. In the Kerguelen Islands, diets primarily consist of earthworms, and lepidopteran and chironomid larvae and pupae^18,19^, whereas in the Marion Islands diets included largely spiders and snails^20^. Barriers to seaward migration and limited access to in-stream prey resources were suggested as contributors to the eventual extirpation of Marion Island brown trout^20^. In contrast, initial colonization success and subsequent spread of the brown trout at Kerguelen was attributed to anadromy, which allowed introduced populations access to the sea and its higher levels of food production. More broadly, adaptability, phenotypic plasticity^21^ and anadromy appear to have facilitated the successful establishment of brown trout on all continents except Antarctica^9^. Previous studies have shown that the marine migratory behaviour of sea-run brown trout is expressed as a continuum ranging from estuarine migration^22,23^, through short to-long-distance (and/or duration) coastal migration^24–27^, with migration flexibility also having facilitated the species’ response to varying conditions in the marine environment.

As an anadromous, iteroparous species, the ability of brown trout to make more than one sea feeding migration may be condition-dependent^25,28^. Factors such as individual body length, sex, and nutritional state are known to influence the spatiotemporal aspects of marine habitat use^27^. For example, in Norwegian populations, larger, nutritionally depleted individuals, especially females, tend to migrate furthest from their home watercourses. This is probably because more distant pelagic areas offer individuals greater foraging opportunities, which can offset the costs of reproduction to somatic body condition and increase future growth, fecundity, and fitness^25,27,28^.

Throughout the Southern Ocean, several sub-Antarctic archipelagos including the Falkland Islands, South Georgia, the Prince Edward Islands, Possession Island, Crozet Island, and Kerguelen, are dominated by environmental features that would seem to favor the successful introductions of salmonids. Brown trout have been successfully introduced to the Falklands^29^ and to Kerguelen^11^, where they have established reproductively successful populations that are known to have colonized adjacent watersheds. However, not all introductions in the sub-Antarctic have succeeded (e.g. Marion Island), despite the environments being mostly free from the anthropogenic stressors that limit salmonids in other parts of their natural range (e.g. fishing pressure, habitat destruction, pollution, warming river temperature, etc.). Furthermore, most sub-Antarctic rivers are naturally fishless, and so interspecific competition is limited or absent. These characteristics make sub-Antarctic rivers excellent model systems in which to examine the factors that promote or impede the successful colonization of natural ecosystems by exotic species such as brown trout.

Previous research at Kerguelen has used traditional sampling and analytical methods (electrofishing to determine species’ presence/absence) and modeling exercises to investigate the dynamics of the brown trout range expansion and colonization from initial introduction sites^10,11^. The modeling studies attempted to predict the rate of the expansion^11^, but contrary to model predictions an unanticipated and unexplained decrease in the invasion rate has occurred. Reasons for this are currently unknown, but it could be linked to possible changes in prevailing environmental conditions (i.e. warming), or changes in the species’ biology and/or behaviour.

Here we examine the pattern and physiological correlates of brown trout migrations along the current front of colonization at Kerguelen. By combining acoustic telemetry, physiological biopsies and dietary information, we also aim to reveal the possible factors that may explain the unexpected decrease in brown trout colonization rates. It has been hypothesized that colonization by brown trout is driven by a small number of “pioneering” individuals, who disperse from natal rivers into the marine environment and onwards to adjacent or nearby rivers where new spawning populations emerge^10^. To test the idea, we biopsied and acoustically tagged sea-run brown trout in an estuary at the colonization front and followed their movements at sea to determine how far from their home river they moved and whether they subsequently returned to the natal river or moved to other rivers. As such, we documented the marine migratory habitat use of brown trout in sub-Antarctic waters (coastal vs. offshore), and the environmental conditions experienced (salinity, temperature). From tissue biopsies, we assessed the physiological condition (energetic metabolites) of the tagged individuals prior to marine entry, which allowed us to explore correlates of movement ecology. We also inferred the feeding ecology of additional untagged brown trout using stomach content and stable isotope analyses.

## Material and methods

### Study area

**Figure 1:**
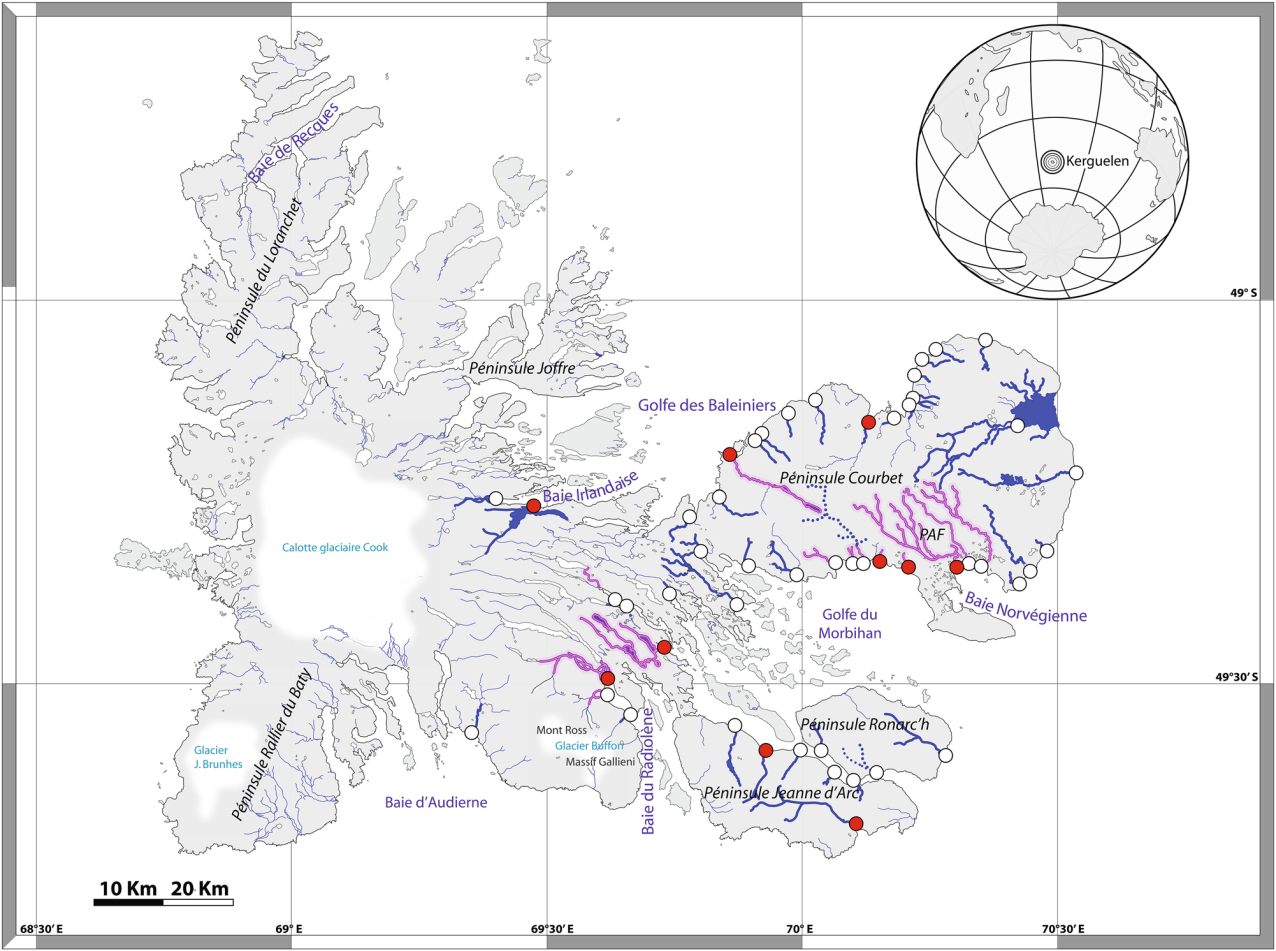
Map of Kerguelen Islands displaying the current distribution area of brown trout. The rivers with a thin blue line are still fishless, those with a blue bold line are populated only by *Salmo trutta*, those with a maroon bold line are populated by *Salmo trutta* and at least one other species (*Salvelinus* spp or *Salmo salar*), and those with a dotted line are populated only by *Salvelinus*. Red dots: locations where brown trout were released by humans. White dots: locations where brown trout introduced naturally. Map obtained with permission from^91^.

The Kerguelen Islands (7215 km^2^) consist of approximately 300 small islands and a single larger one called “La Grande Terre”, which accounts for 92% of the land surface of the archipelago (Fig. 1). Kerguelen is located at the convergence of the Southern and Indian Oceans (49°30′S–69°30′E), north of the Antarctic Polar Front that delimits Antarctic from sub-Antarctic waters^30^. The main island is characterized by an environmental cline, where the eastern side is a mix of lowland plains and mountains with relatively dry weather (rainfall < 500 mm per year). In contrast, the west is dominated by mountains and glaciers, with heavy precipitation and exposure to prevailing westerly winds and rainfall > 2000 mm per year^10^. The general dynamics of the landscape have been recently affected by climate change, with rapidly melting glaciers in the west^31,32^ that have changed the riverine ecosystems used, or potentially usable, by brown trout.

The freshwater systems at Kerguelen present a range of temperature conditions, from relatively warm waters (that may exceed 20 °C during the austral summer) to consistently cold waters in mountain lakes and river systems fed by glaciers^10^. The native freshwater invertebrate community is taxonomically poor and dominated by Chironomidae and zooplankton^33–35^.

At Kerguelen (Fig. 1), almost all the accessible watersheds on the eastern half of the “La Grande Terre” are now considered to contain brown trout^10^. In the east, river flows are predominantly driven by precipitation, while in the west flows are strongly influenced by meltwater from glaciers (Mount Ross and the Cook Ice Cap). In the westernmost part of the island, only a few rivers have been colonized or are in the early stages of colonization^11^; Gaudin, unpublished data. However, the estuaries of rivers along the 120 km western coastline are difficult to access either by sea or by land due to rough ocean conditions, their steep topography, and the presence of cliffs several hundred meters high, that make comprehensive assessments of colonization activity difficult.

**Figure 2:**
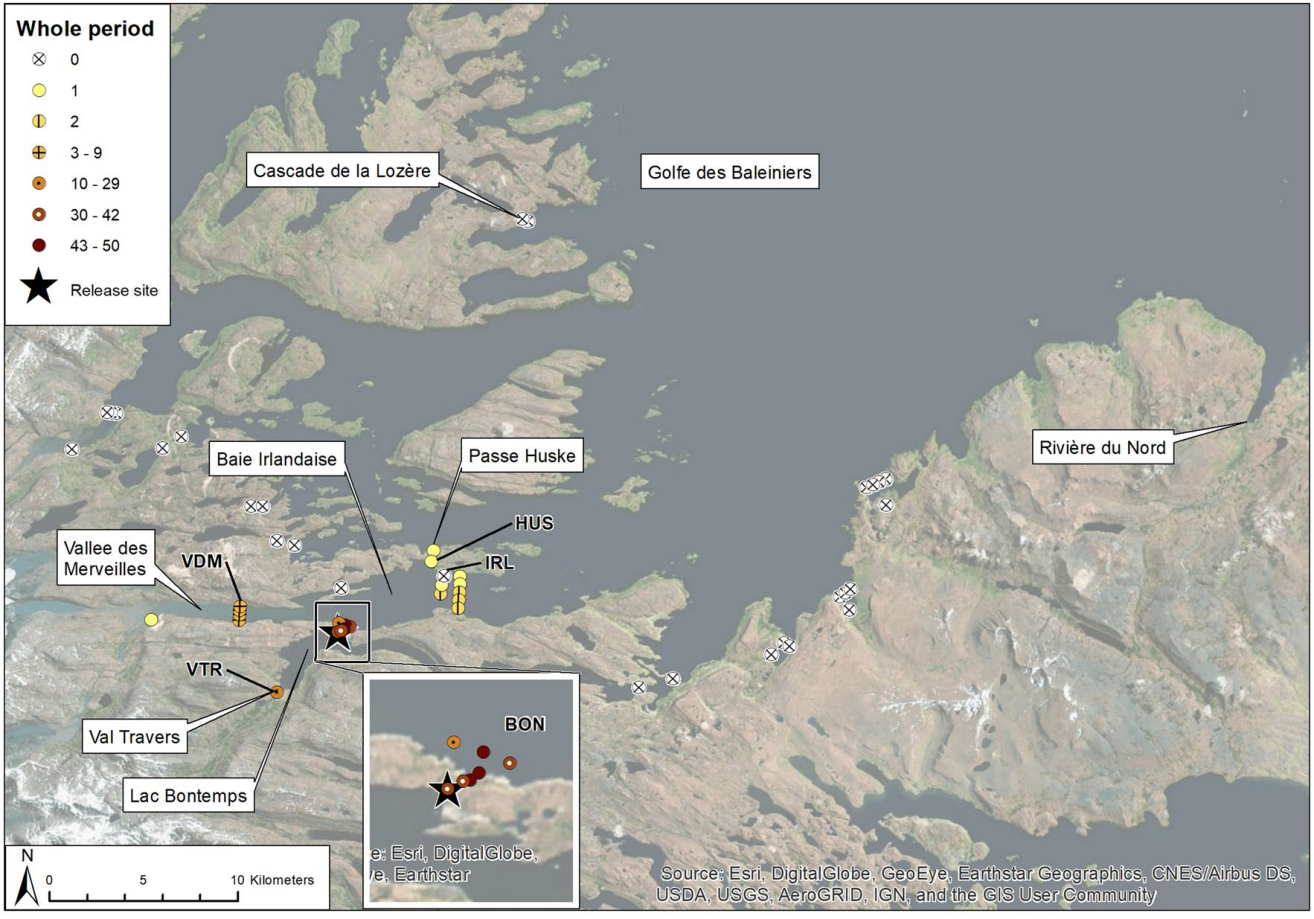
Map of the acoustic receiver array deployed (*n* = 50 receivers). Symbols give the location of individual receivers and number of individual sea trout registered at each receiver. The tagging site (large star) was located next to the outlet of Lake Lac Bontemps. The map was powered by ArcGIS 10.5 (https://support.esri.com/en/products/desktop/arcgis-desktop/arcmap/10-5).

The brown trout examined in this study were captured from the north-eastern side of Kerguelen, at the estuary of the coastal freshwater Lake Bontemps (7 Km^2^; Fig. 2), which is fed by the Val Travers River and drains into the marine fjord Baie Irlandaise by way of a 6 m high steep, but traversable, outlet (49°16′49″S—69°28′38″E). The estuary is small (about 0.01 Km^2^) but offers a wide variety of habitats and shelters for fish in brackish water. Water temperatures measured by thermistors incorporated into acoustic receivers (see description below) during the study period (Jan 2018–Jan 2019) varied from 1 to 11 °C at 1 m depth in the Lake Bontemps estuary (3 m maximum depth) to 1–11 °C at 20 m depth at the outlet of Baie Irlandaise (25 m maximum depth). Water temperatures measured during the study period are available from Davidsen et al.^36^. Accordingly, the fjord and estuary are ice-free all year.

Brown trout were first introduced into the Val Travers River in 1993 with 2000 fry originating from Kerguelen’s Rivière Chateau (Fig. 1) population where brown trout were introduced in 1962. The density of fish older than 2 years in the river was estimated in 2010–2012 using a De Lury sampling protocol, with the average density equalling 27,600 trout/Km^2^^37^.

### Capture, blood sampling and tagging of brown trout

During the austral summer of 2018 (27 Jan–10 Feb) a total of 50 brown trout (mean total length (L_T_) (± SD) = 530 ± 45 mm, range 390–615 mm) were captured in the estuary of the Lac Bontemps outlet using a combination of angling (n = 36) and gillnetting (n = 14; 35–42 mm mesh size). Strong winds during fieldwork, e.g. reported averages for Kerguelen are 66 km h^−1^ with wind in all months^38^, rendered field determinations of live body mass unreliable due to scale drift, and so mass data were excluded from analyses. Efforts were made to reduce stress and the risk of injury by minimizing angling fight times and by monitoring the gill net continuously (e.g., the net was pulled in as soon as vibrations were seen or felt). Shortly after landing and prior to anaesthesia, ~ 2 mL blood samples were collected via caudal venipuncture from all fish using literature protocols^39^. The mean time to blood sampling, as calculated from the time of hooking, was 4 min 17 s (range 02:31–07:20). Following blood sampling, fish were kept for no more than 6 h in a net cage situated in the river to allow recovery from capture and sampling prior to tagging. For tagging, the fish were anaesthetized with 2-phenoxyethanol (EC No. 204-589-7; SIGMA Chemical Co., USA; 0.5 mL L^−1^ water). An acoustic transmitter (Thelma Biotel, model CT-MP9L, 9.0 × 42.5 mm, mass in water 4.2 g, power output 146 re 1 μPa at 1 m, estimated minimum tag duration 300 days) was externally attached to the fish below the dorsal fin with 0.5 mm steel wires inserted horizontally through the upper part of the dorsal musculature. The wires were threaded by using two hollow needles (1.25 mm in diameter), sharpened at one end. The needles were pushed through the musculature approximately 20 mm below the dorsal fin. The spacing between the needles matched the length of the tag. To avoid erosion of the flesh by the tag or attachment wire, a silicone plate was attached between the tag and the skin, and a plastic plate was attached between the skin and the wire on the opposing dorsal side^40^. Throughout the tagging process, the gills were irrigated with fresh, ambient river water. For all fish a small part of the adipose fin was clipped and stored in alcohol for later genetics analysis of sex. After tagging, the fish were released in a calm part of the Bontemps estuary or near the shoreline close to the capture site.

To measure levels of salinity and water temperature encountered by the individual fish, each acoustic tag was equipped with a sensor for conductivity (range 0–34‰, resolution 2‰) and temperature (range 0–15 °C, resolution 1 °C). The tags were programmed to sample every 45 s and the most recent values, along with the individual fish ID, were transmitted with a random 30–90 s interval delay.

The care and use of field-sampled animals complied with the Government of France animal welfare laws, guidelines and policies (Comité d'Ethique) as approved by the Terres Australes et Antarctiques Françaises administration under the auspices of permit number (Référence du dossier) 2016122009113932-v2. All methods are reported in accordance with ARRIVE guidelines.

### Deployment of acoustic receivers

The tagged fish were tracked using a total of 50 acoustic receivers (InnovaSea (formerly Vemco Inc.), Canada, models VR2-AR and VR2W), 52 were originally deployed but two were lost. Of the 50 recovered, 43 were deployed in the marine fjord systems, while seven were deployed in accessible watercourses proximate to the tagging site (Fig. 2). All receivers deployed in the fjords were mounted on moorings at depths of 6–38 m (average of 18 m) below the surface and were operative from January 2018–January 2019. In freshwater, receiver depths ranged from 0.5 to 1.5 m.

### DNA sex determination

DNA was extracted from ethanol-preserved fin clips with a NaCl chloroform protocol^41^. The extraction volume was 100 µL. DNA quality was controlled on 1.5% agarose gel. Sex was determined by PCR amplification of a 200 base pair (bp) fragment situated in the first intron of the male-specific SDY gene, using the Salmo-sdY-F and Salmo sdY-R primers^42^ and a control fragment which amplified on both female and male DNA (control sex F-R1). The PCR was performed in a final volume of 10 µL using the Qiagen Type-it Microsatellite PCR kit, with 20 ng of DNA, and 0.2 µM of each primer. The following PCR profile was used: a first denaturation of 95 °C for 5 min, followed by 35 cycles of 95 °C for 30 s, 57 °C for 1 min 30 s, 72 °C for 1 min, with a final extension at 68 °C for 10 min. Sex was scored by running the PCR products on 3.5% agarose gels.

### Analyses of blood samples

The blood samples taken during the tagging procedure were stored in tubes and immediately placed in chilled (7 °C) sea water for up to 3 h before being centrifuged at 1163 g for 10 min. Blood glucose levels were measured on site using the commercially-available Accu-Chek Aviva Nano meter system (Roche Diabetes Care, Inc., USA). Blood plasma was flash-frozen in a LN_2_ dry shipper and subsequently stored at − 80 °C until biochemical analyses could be carried out. Plasma triglyceride levels were spectrophotometrically determined, in duplicate, using the manufacturer’s suggested protocols with a commercially available colorimetric kit (Cayman Chemical Company, USA). The average intra-assay coefficient of variation (CV) for plasma triglycerides was 1.68% (range 0.02–6.27%) and the average inter-assay CV was 2.07%. Total plasma protein levels were determined using a Bradford’s assay^43,44^ using commercial reagents (Bio-Rad Laboratories (Canada) Ltd., Mississauga, ON, Canada).

### Analyses of feeding ecology

An additional 31 non-tagged sea-run brown trout (total length = 515 ± 113, range = 240–670 mm; mass = 1906 ± 962, range = 140–3730 g) were collected from the Lac Bontemps estuary (*n* = 10), Cascade de la Lozère (*n* = 5) and the Rivière du Nord (*n* = 16). Dissection of these fish permitted us to assess stomach fullness (see below), and sex was determined by visual examination of gonads. From each individual, 10–20 scales were removed and stored in envelopes for age determination while stomach contents were preserved in 96% ethanol for further examination. Dorsal muscle tissue (10 g) was excised from each fish from above the lateral line and posterior to the dorsal fin and was frozen and then oven-dried at 50 °C for > 48 h for use in stable isotope analyses (δ^13^C, δ^15^N).

Stable isotope analyses (δ^13^C, δ^15^N) of the muscle tissue and prey items followed methods described in the literature^45,46^ and were conducted at the University of Waterloo Environmental Isotope Lab using a Delta Plus Continuous Flow Stable Isotope Ratio Mass Spectrometer (Thermo Finnigan, Bremen, Germany) coupled to a 4010 Elemental Analyser (CNSO 4010, Costech Analytical Technologies Inc., Valencia, USA). Analytical accuracy was ± 0.2% (δ^13^C) and ± 0.3% (δ^15^N), and analyses were calibrated against the International Atomic Energy Agency standards CH3 and CH6 for carbon and N1 and N2 for nitrogen. All results are reported relative to the international standard Vienna Peedee Belemnite for δ^13^C^47^, and atmospheric nitrogen for δ^15^N^48^. As C:N ratios in general did not exceed 4 (1 of 31 samples), lipid extraction or correction using mathematical models was not needed^49^.

### Analyses of stomach contents and age determination

A volumetric analysis of stomach fullness was conducted following literature protocols^50^. Stomach contents from the upper end of the oesophagus to the pyloric sphincter were identified to the lowest practical taxonomic level (typically order or family) under a stereoscopic microscope. The relative importance of each prey category was evaluated as volume % for each stomach and the total volume of the food category taken by all sampled fish with stomach contents was expressed as a percentage of the total volume of each stomach content^50^. Age was estimated from scales following literature^51^.

### Data analyses

Acoustic data were filtered to account for environmental effects that could have biased our interpretations. Signal collisions from simultaneously transmitting tags or noise in the environment can generate false detections^52^. Thus, an automated filter excluding single detections within a time span of eight hours was applied to all receiver data prior to statistical analysis. The filter excluded 1377 of 2055671 detections (0.07%) from further analyses. Data from three individuals, which had less than three days of recordings, were also excluded from further analyses.

To describe the environmental conditions (i.e., temperature and salinity) experienced by tagged individuals and the proportion of individuals present in the different habitat types throughout the year, we created three daily salinity categories. A trout was considered to have been: (i) resident in fresh water for a day if the average of all salinities reported by the sensor over a 24 h period was < 1.3‰; (ii) in brackish water for a day if the average daily salinity was between 1.3 and 30‰, inclusively; or iii) in salt water for a day if the average daily salinity was > 30‰. The average daily temperatures measured by the acoustic tags on individual fish in these three different habitat types throughout the year were then calculated and plotted.

Given the pronounced effect of sea spray sulfates on coastal δ^34^S values and the notable gradients in δ^13^C across freshwater water- marine interfaces e.g.^53−55^, δ^13^C values were used in a two end-member linear mixing model analysis^56–58^ to estimate the proportion of marine-sourced carbon (PMC) in the diets of sampled brown trout. Computations were corrected to account for trophic fraction between predator and prey using the mean trophic fraction value of 0.4‰ reported in Post^59^. End-members (mean ± SD) consisted of sampled freshwater plankton (− 25.6 ± 0.7‰), and pooled data obtained from the literature^60^ for echinoderms sampled from coastal Kerguelen habitats (− 15.8 ± 2.1‰).

To characterize the magnitude of the shift in trophic space (δ^13^C–δ^15^N) associated with anadromy, the Euclidean distance between a mean freshwater reference point (freshwater zooplanktivorous feeding Arctic charr, *Salvelinus alpinus*^46^) and the isotope values for each fish were computed. To characterize the direction of change in trophic (δ^13^C–δ^15^N) space associated with anadromy, the angle of change measured as the counter clockwise angle between the horizontal δ^13^C axis and the vector describing the magnitude of change was measured e.g.^61^.

The difference between sites was assessed using analysis of variance, Welch's t-test^62^ or circular statistical methods^61,62^ as appropriate for the data being analysed. Finally, to contextualize the trophic status of Kerguelen anadromous brown trout, comparisons were made to other literature reported values for invertebrates (e.g., echinoderms, *Thermisto sp*. cephalopods) and myctophid mesoplegaic fishes captured in nearshore Kerguelen waters^60,63,64^. Data from all of these were used to construct standard stable isotope cross-plots (mean ± SD along each isotope axis). Data used in this study are available from here https://doi.org/10.14286/2021-KERG-BROWNTROUT-SCIREP.

## Results

### Overall movements of acoustically tagged fish

No tagged brown trout were registered on the receivers located outside the Baie Irlandaise fjord connecting Lake Bontemps and Vallee des Merveilles (Fig. 2; S1 Supplementary Material). Of the 43 fish detected in the marine fjord, nine individuals (21%) visited the Vallee des Merveilles estuary 6 km west of the tagging site at Lake Bontemps estuary and two fish visited the Baie Irlandaise receiver area 6 km east of the Lake Bontemps estuary. Of the two fish visiting the Baie Irlandaise receiver area, one individual stayed next to the receiver area for a few hours before returning to the inner fjord, while the other individual exited the fjord for 6 days before returning, although it was not registered on any of the receivers positioned outside of the fjord. Of the 43 brown trout registered in the marine fjord, 28 (65%) migrated to Lake Bontemps for overwintering. The mean date for migrating to freshwater was 20 March (*n* = 28; range 2 Dec–30 June), while the mean date for migration back to the marine fjord was 18 September (*n* = 17; range 1 May–23 November). During the winter, fifteen of the fish were recorded one or several times in the inlet river to Lake Bontemps (Val Travers).

### Proportion of sexes and nutritional state of the acoustically tagged fish

Based on the genetic determinations, 20 fish (40%) were females and 30 fish (60%) were males. Mean level of plasma triglyceride of tagged fish was 2.45 mmol/L (SD ± 1.54; range 0.18–5.92). There were no significant differences between males and females (Anova; *P* > 0.05, *n* = 50). The mean level of blood glucose in the fish was 3.2 mmol/L (SD ± 0.4; range 2.2–3.9) and again there was no significant difference between males and females (Anova; *P* > 0.05, *n* = 50).

### Mortality or tag expulsion

While more than 50% of the brown trout were regularly detected up to 10 months after release (S2, Supplementary Materials), a number of individuals (n = 11) were lost to the study through mortality, tag expulsion or predation followed by the predator excreting the tag. These assessments were based on persistent stationary recordings of particular tags at particular places. Four were lost between 11 and 23 March, three more in June, and one in August.

### Salinity and temperature experienced

**Figure 3:**
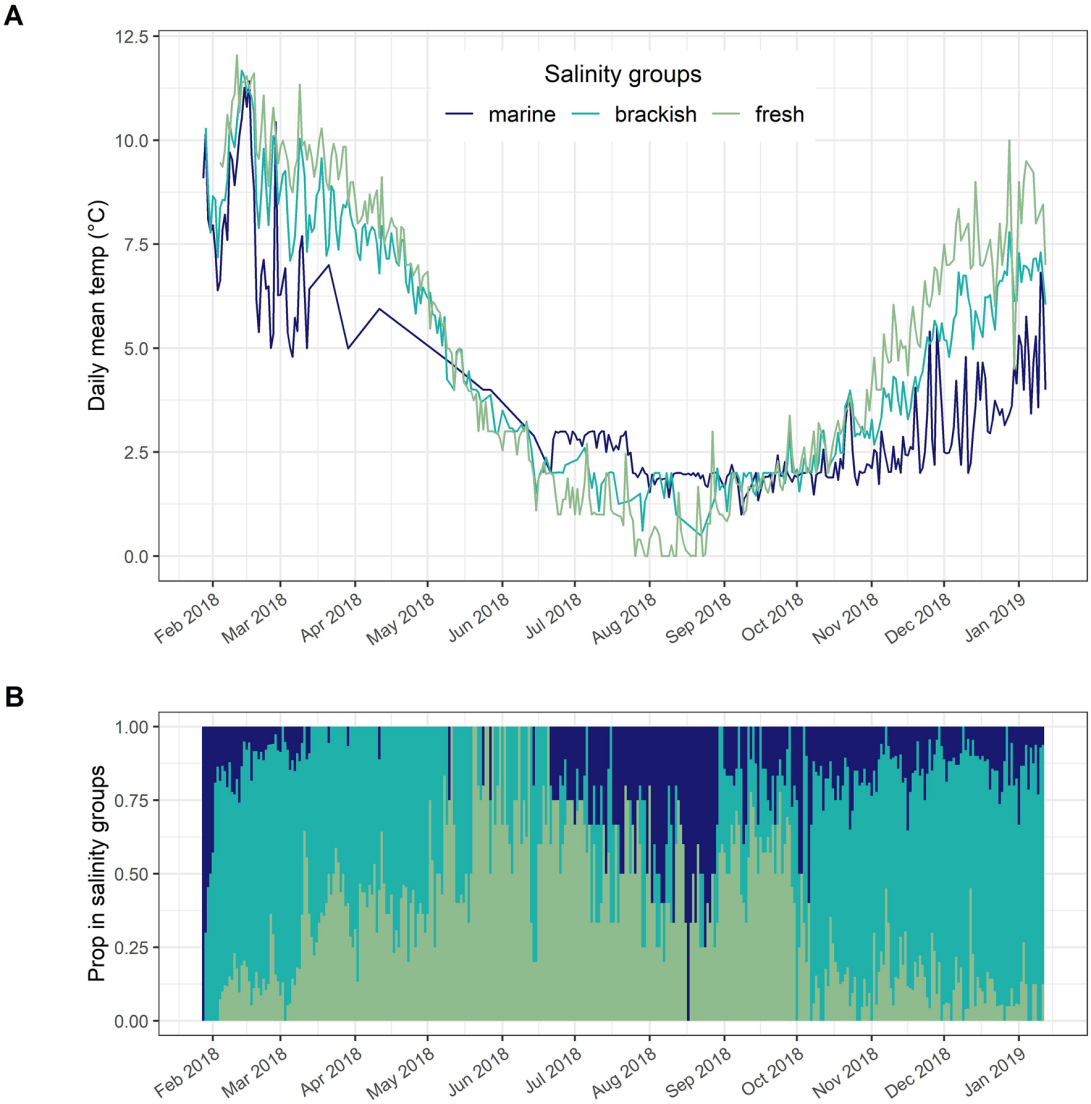
Salinity and temperature levels experienced by brown trout tracked in the Lake Bontemps estuary and nearby areas. (**A**) daily mean temperature experienced by the fish in three salinity categories (fresh (< 5‰), brackish (5–30‰) and marine waters (> 30‰); (**B**) daily proportion of fish divided in each salinity category (fresh, brackish and marine waters). The proportions are based on the relative sample size for the actual day.

The brown trout feeding in the Lac Bontemps estuary were exposed to fluctuating levels of salinity and water temperature (Fig. 3; S1 and S3 Supplementary Material). From February to May, median levels reported by the acoustic tags were in the range of 5–15‰, increasing to 25–35‰ in July–October and subsequently decreasing to 10–25‰. Median water temperatures utilized by brown trout varied from 12 °C in February to 2 °C in August–October (Fig. 3). Individuals residing in Lac Bontemps experienced a similar range of temperatures, ranging from 12 °C in late February to 0 °C in August–September. Mean water temperatures experienced by individual fish during the feeding season (1 Oct 2018–28 Feb 2019; Table 1) were 2.4 °C C higher in the brackish water habitat (6.6 °C) than in the marine habitat (4.2 °C).

**Table 1:** Mean temperatures experienced by the brown trout during from 1 Oct 2018–28 Feb2019.

Feeding area	Mean temperature (°C)	SD	Range	*n*
Marine water	4.2	2.9	1.0–12.5	45
Brackish water	6.6	2.8	0.2–12.9	47
Freshwater	5.6	3.5	0.0–12.6	46

n: number of unique fish with temperature measurements in each feeding area. Freshwater: < 5‰; Brackish water: 5–30‰; marine waters: > 30‰.

### Feeding ecology

The 31 individuals (16 males; 15 females) sacrificed for an analyses of feeding ecology mainly fed on amphipods (68% by volume), isopods (6%), fish (23%) and zooplankton (6%) and exhibited good condition (mean Fulton's F = 1.22). Other identified prey items included: beetles, larval insects, krill and crabs. Three fish (10%) had empty stomachs. Low prevalence and intensity of the endoparasite *Eubothrium* sp. was noted as 28 (90%) of examined fish had no, or only a few, *Eubothrium*). In 30 (97%) fish muscle colour was red or pink and indicative of a probable feeding preference for Gammaridae.

**Figure 4:**
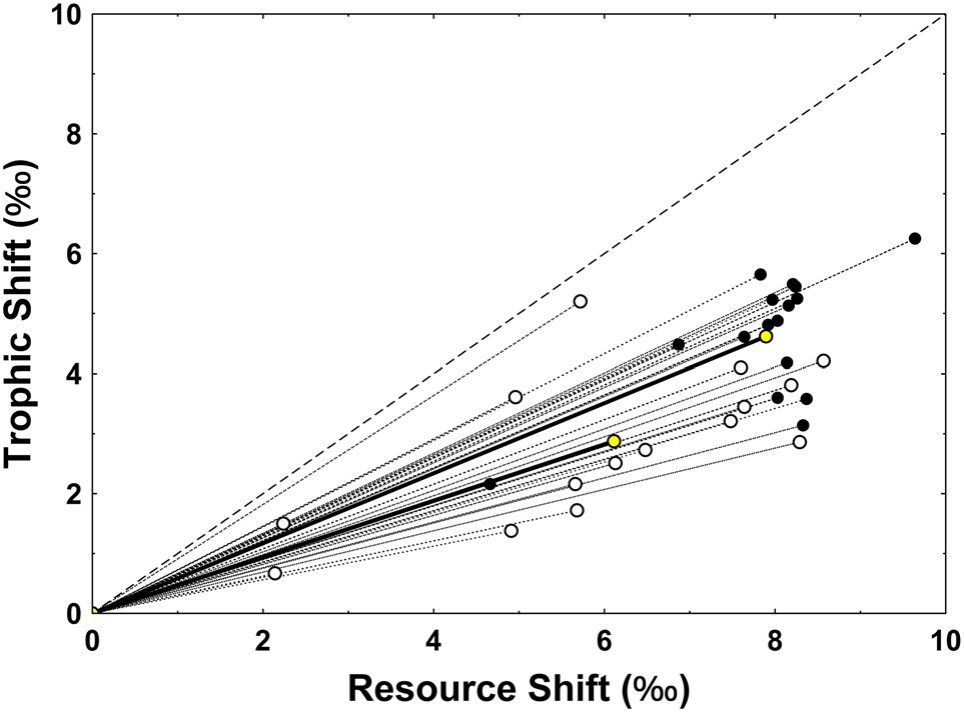
Net displacement of brown trout in isotopic (δ^13^C, δ^15^N) space as a result of moving from freshwater to marine feeding. Black and white dots, respectively, plot the individual isotope signatures of marine-captured trout from Rivière du Nord and Golfe des Baleiniers sample sites. Lines connecting each point to the origin define the magnitude (Euclidean distance) and angle of the shift. All lines fell below the 45° line (large dashes), indicating that trophic niche shifts were dominated by greater changes in δ^13^C than δ^15^N. Yellow dots and associated heavy solid lines plot the means and mean change vectors for the Rivière du Nord (upper dot) and Golfe des Baleiniers (lower dot) sample sites. The angular change, measured as the counter-clockwise angle from the resource shift axis, was significantly greater for the Rivière du Nord than the Golfe des Baleiniers sampled fish.

The stable isotope values of examined brown trout ranged from -23.28 to -15.78‰ for δ^13^C and 7.7‰ to 13.29‰ for δ^15^N. Differences between capture sites were evident, with fish captured proximate to Rivière du Nord (Eastside of Golfe des Baleiniers) having higher mean δ^13^C (F_1,29_ = 9.987, *P* = 0.004) and δ^15^N (F_1,29_ = 17.769, *P* < 0.001) values than fish captured in bays along the western side of the Golfe des Baleiniers. Rivière du Nord fish similarly showed greater proportional reliance (mean ± SD) on marine sourced carbon (0.783 ± 0.103) than the Westside Golfe des Baleiniers fish (0.603 ± 0.202), with the difference between sites being significant (F_1,29_ = 9.987, *P* = 0.004). Regardless of site, the observed trophic niche shift resulting from anadromy was dominated more by changes in δ^13^C than δ^15^N (Fig. 4), with the mean shifts in δ^13^C and δ^15^N, respectively, for Westside Golfe des Baleiniers fish equaling 6.11 and 2.87‰ and the mean shifts in δ^13^C and δ^15^N, respectively, for Rivière du Nord fish equaling 7.89 and 4.62‰. Changes in δ^13^C were significantly greater than changes in δ^15^N at both sites (Westside of Golfe des Baleiniers: t = − 5.338, df = 28, *P* < 0.001; Rivière du Nord: t = − 8.909, df = 30, *P* < 0.001). Overall the magnitude of the shifts in trophic niche as characterized by the Euclidean distance between individual stable isotopes values and a freshwater reference point based on freshwater feeding zooplanktivorous fish indicated greater increases in trophic position in Rivière du Nord fish than in Westside Golfe des Baleiniers fish (F_1,29_ = 13.649, *P* = 0.001). The Watson-Williams test for equal means similarly showed greater angular adjustment (30.04°) in Rivière du Nord fish than in Westside Golfe des Baleiniers (24.9°) fish (Williams–Watson U = 5.364, *P* = 0.028 and Fig. 4).

**Figure 5:**
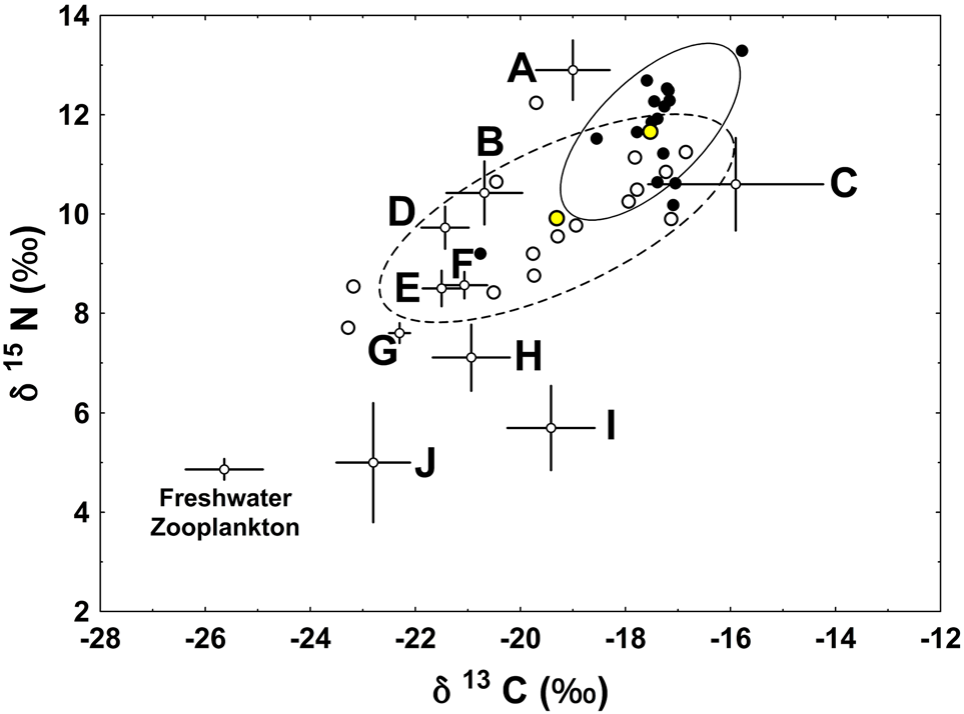
Stable isotope bi-plot of brown trout (circles) and Kerguelen invertebrate and fishes (black squares and crosses) grouped by Genus. Solid and dotted lines, respectively, plot the standard ellipse area (SEA_C_) for Rivière du Nord (black circles) and Golfe des Baleiniers (open circles) sampled brown trout. Ellipse means are denoted by the yellow circles. Freshwater zooplankton are labelled and provided as a freshwater reference value. Species plotted (and data sources) include: A—*Dissostichus eleginoides* (Cherel et al.^63^); B—*Cephalopod* sp. (Cherel et al.^63^; Guerreiro et al.^64^); C—*Ecinoderms* (Saucede et al.^60^)*;* D—*Gymnoscopelus* sp. (Cherel et al.^92^); E—*Protomyctophum* sp. (Cherel et al.^92^); F—*Electrona* sp. (Cherel et al.^63^; Cherel et al.^92^); G—*Krefftichthys andersonii* (Cherel et al.^63^); H—prey fish species samples obtained from brown trout guts (this study); I—nearshore sampled amphiod sp. (this study); J—*Thermisto* sp. (Cherel et al.^63^).

When compared to literature data for resident nearshore invertebrates, cephalopods and myctophid mesoplegaic fishes, sampled brown trout were largely bounded by *Protomyctophum* sp. (lantern fishes) and *Echinoderms* on the δ^13^C axis and *Krefftichthys anderssoni* (Rhombic lanternfish) and *Dissostichus eleginoides* (Patagonian tooothfish) on the δ^15^N axis (Fig. 5).

## Discussion

In this study, no acoustically tagged brown trout were observed exploring previously uncolonized watercourses. In general, the tagged individuals remained close to their presumed home river during the marine feeding migration. The restricted migratory distances observed here (< 7 km) were much smaller than observed in other tracking studies of brown trout (typically 15–30 km) in marine fjord habitats^24,25,27,28^. Brown trout generally remain in near-coastal areas during their seaward migrations, close to their river of origin (< 80 km), and on the Patagonian Continental Shelf, brown trout have never been reported in the catch record further than 50 km from their rivers of origin^65^. Nevertheless, there are reports of individuals making very long-distance marine migrations at sites in the Northern hemisphere > 500 km^66^, and so the extent of marine migrations by this species shows great plasticity and deserves further study.

As for all anadromous salmonids, the marine migration is an important life-history strategy for brown trout. Coastal marine environments, like that at Baie Irlandaise, generally have higher levels of primary and secondary production and are better sources of nutritionally important fatty acids^67,68^ than the oligotrophic rivers that flow into it. Consequently, brown trout can exploit better feeding conditions in the Baie and achieve higher growth rates, larger sizes-at-age, and higher fecundities than they would if they remained exclusively in freshwater.

This has been corroborated by previous studies of anadromous brown trout in Kerguelen^69^ and in Patagonian Rivers^70^. However, potential costs of migration to sea could affect physiological processes, change energy allocation for swimming, and result in a greater probability of mortality due to the increased risks of predation, parasitism, and disease^71,72^. Additionally, a distant or pelagic marine migration versus an estuarine or near-shore residency could be driven by a tradeoff between predation risk and foraging opportunity, mediated by intra-specific competition. The Baie Irlandaise is occupied by several marine predators such as Antarctic fur seals (*Arctocephalus gazella*), and gentoo (*Pygoscelis papua*) and king penguins (*Aptenodytes patagonicus*), all of which could possibly prey on brown trout and/or compete for prey. Although gentoo penguins have been observed swimming into Kerguelen rivers in search of prey^73^, it is likely that predation risk for the trout is higher further out in the bay than in the estuary or river.

While marine migrations are risky, energetic demands driven by the costs of reproduction and growth may determine whether or not a fish moves to sea. It has been shown that individual brown trout, particularly females with low somatic energy stores in spring-time, will migrate further away from the home watercourse to search for prey and facilitate nutritional and somatic re-conditioning^27,28,74^. Therefore, it might be predicted that these far-ranging individuals are more likely to encounter and explore new freshwater habitats. However, we did not observe far-ranging or exploratory behaviour in this study, and from the analysis of blood samples collected at the beginning of seaward migration, nutritional indicators suggest that all fish were in relatively good condition at the time of tagging (triglycerides levels, mean = 1.54 mmol/L; glucose, mean = 3.2 mmol/L; see^41^). Indeed, contemporaneously sampled brown trout used for stable isotope analysis had excellent body condition (high Fulton's Condition Factors). Based on these nutritional data, and the results of our stable isotope analysis, it appears that the brown trout in the Baie Irlandaise had good access to rich, local feeding opportunities and were able to meet reconditioning/growth energetic demands locally without having to incur the increased risks of long seaward migrations.

The importance of food related shortages as a determinant of migration dispersal has been documented elsewhere. For example, studies in Newfoundland (Canada) suggest that large, productive watersheds are more likely to experience successful brown trout invasions^75^. McDowall et al.^29^ similarly noted the importance of resources as a trigger for movement in the Falkland Islands (another sub-Antarctic island group), with seaward brown trout migrations thought to be related to low biological productivity and the sparseness of food in freshwater streams. Food availability in Patagonian rivers has also been suggested as a key determinant of the decision to migrate, although genetic and other environmental factors are also likely involved^70,75^.

The temperatures experienced by the tagged brown trout during their feeding migration (range 2–13 °C) were in general lower than the 12 − 17 °C range reported as optimal for growth in brown trout^76–78^. It has been suggested that brown trout optimize growth at sea by seeking temperatures within this range. For example, studies from Denmark have indicated that brown trout actively avoided temperatures higher than 17 °C by diving to colder waters, while studies in northern Norway, close to the northern boundary of the species’ distribution, have shown that brown trout reside close to the surface and seek areas in the fjords with higher temperatures^79,80^. In the current study, the mean temperature experienced by brown trout feeding in the estuary was 2.4 °C higher than for individuals feeding in more marine waters. Temperature optimization is apparent in the changes in the relative use of fresh and brackish waters versus marine waters, with higher proportionate use of fresh and brackish waters observed for most of the year except the mid-June to mid-August period when marine temperatures typically exceeded those in fresh and brackish waters (Fig. 3).

During the marine feeding migration from September to March, most of the brown trout stayed in brackish water (5–25‰) in, or near, the estuary of the natal river. To our knowledge, this is the first published report on in situ measurements of salinity levels experienced by free-ranging brown trout in a marine habitat but see^40^. Based on previous studies, it has been shown that brown trout are variable in their use of estuaries. Some brown trout remain in the estuary^24,28,81,82^ throughout the feeding season, while others move more quickly through the transition zone and into marine waters^24,28,79^.

The brown trout at Lake Bontemps migrated to sea from May to November (mean date 18 September) and returned from December–June (mean date 20 March). Previous studies have found that spawning at Kerguelen most likely occurs between July and August, possibly even starting in June^69,83^, which probably is an adaptation to foster the survival of eggs and progeny. Fry emergence occurs in December–early January at the beginning of summer when temperatures range from 5 to 9 °C and more food is available^83,84^. Within its natural range, anadromous brown trout generally spend the boreal summer months at sea, but display marked variation in the timing and duration of their marine migration^66^. The spawning period is variable among watercourses but in the species natural range in the northern hemisphere occurs typically sometime between late September and early November. The seasons are reversed in the Southern Hemisphere, and we might reasonably expect the timing of important life history events for sea trout like seaward migration and spawning to be shifted by about 6 months compared to when these events occur in the North. At Kerguelen, this expectation held true for the seaward migration, where timing was offset compared to northern populations by 6 months. However, the pattern for spawning differed. In the north, spawning typically occurs in the autumn (September to November), whereas at Kerguelen spawning occurred in the winter (July–August) This suggests that facultative behaviors like migration have adapted more quickly to the austral summer than patterns of reproductive behaviour, which appears to need more time to adapt.

Our analyses show that anadromous brown trout at Kerguelen mainly feed on amphipods and fish, but also on isopods and zooplankton, and undergo marked shifts in the diets when moving from fresh to estuarine and marine feeding. Trophically, brown trout feeding in nearshore waters at Kerguelen resemble anadromous brown trout introduced to Patagonia in South America, which feed toward the top of nearshore foodweb, and can likely be functionally grouped on the basis of their stable isotope values with other predators on intermediate-sized fishes and cephalopods^85^. Furthermore, the trophic position of brown trout reported here is similar to that reported from coastal systems in the northern hemisphere^86–90^ and highlights their generalist foraging strategy. At Kerguelen, brown trout from Rivière du Nord had a higher level of marine prey in their diet than those from Lac Bontemps and Cascade de la Lozère and relied more on fish than individuals from the other two populations. Rivière du Nord was stocked with brown trout resulting in its colonization in 1981, 12 years before Lac Bontemps was similarly stocked and colonized in 1993, and 36 years before Cascade de la Lozère was naturally colonized in 2017 (see^11^, Gaudin unpublished data). These dietary differences suggest that the more established population of brown trout at Rivière du Nord migrate further from their home river than the less established populations at Lac Bontemps and de la Lozère.

With regards to the observed and unexplained pause in the spread of brown trout to the other rivers of Kerguelen^10^, our results suggest that the anadromous fish at the colonization front are currently nutritionally and energetically healthy and consequently did not need to venture far from their home. Limited movements by fish from the site may continue until population density increases to the point where intra-specific competition reduces feeding opportunities and fish condition to the point where trade-offs between feeding opportunities and predation risks favour more extensive and expansive ocean feeding. Thus, we might predict that it is the energetically challenged individuals that will venture furthest into the Baie Irlandaise during feeding migrations (i.e., condition-dependent risk-taking) and that those fish will have the highest likelihood of straying to new, currently fishless rivers, and starting new populations.

## Supplementary Information

Supplementary Information.
