# Supplementary material for: Marine habitat use and feeding ecology of introduced anadromous brown trout at the colonization front of the sub-Antarctic Kerguelen archipelago
Source: Sci Rep. 2021 Jun 7;11:11917. doi: 10.1038/s41598-021-91405-x (PMC8184814; doi:10.1038/s41598-021-91405-x)
Supplement: Supplementary file 1 — Supplementary Information. [file 41598_2021_91405_MOESM1_ESM.pdf]

Marine habitat use and feeding ecology of introduced anadromous brown trout at the colonization front of the sub-Antarctic Kerguelen archipelago

Jan G. Davidsen[1]*, Xavier Bordelau[2,3], Sindre Håvarstein Eldøy[1], Frederick Whoriskey[4], Michael Power[5], Glenn T. Crossin[2], Colin Buhariwalla[6] and Philippe Gaudin[7]

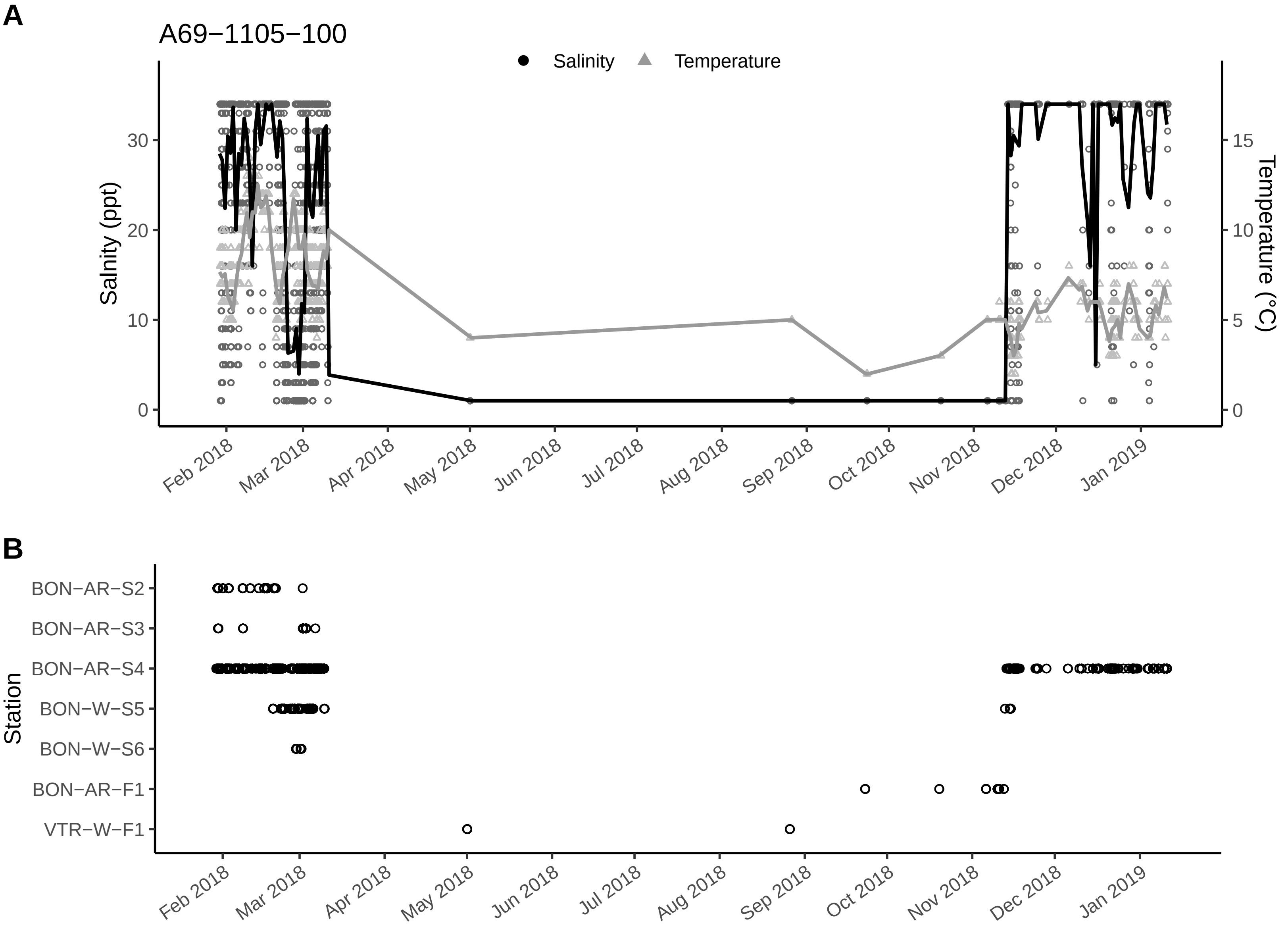
A
A69−1105−100
Salinity
Temperature
Salnity (ppt)
Temperature (°C)
0
10
20
30
0
5
10
15
Feb 2018
Mar 2018
Apr 2018
May 2018
Jun 2018
Jul 2018
Aug 2018
Sep 2018
Oct 2018
Nov 2018
Dec 2018
Jan 2019
B
Station
BON−AR−S2
BON−AR−S3
BON−AR−S4
BON−W−S5
BON−W−S6
BON−AR−F1
VTR−W−F1
Feb 2018
Mar 2018
Apr 2018
May 2018
Jun 2018
Jul 2018
Aug 2018
Sep 2018
Oct 2018
Nov 2018
Dec 2018
Jan 2019

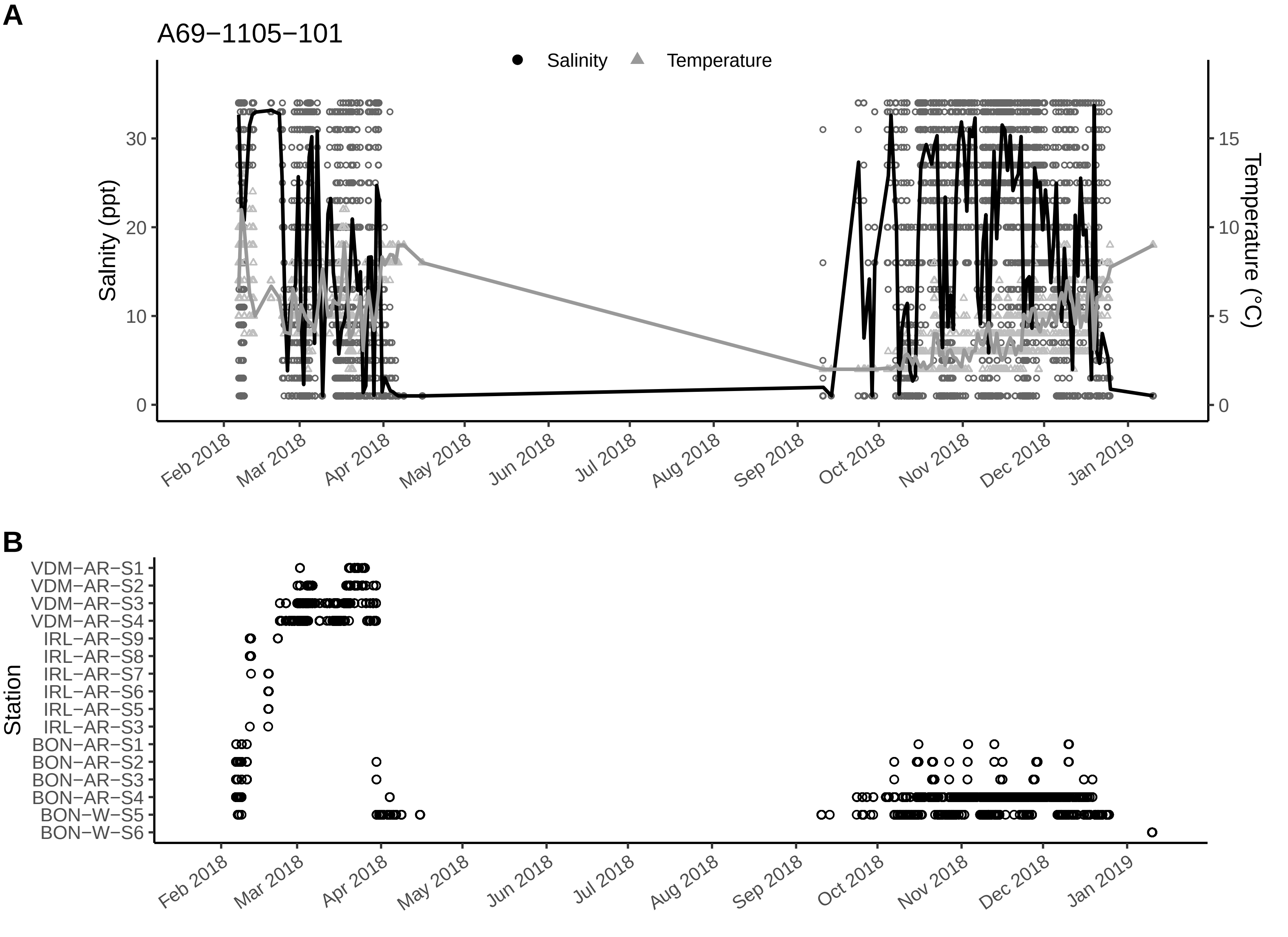

A
A69−1105−101
Salinity
Temperature
Salinity (ppt)
Temperature (°C)
Feb 2018
Mar 2018
Apr 2018
May 2018
Jun 2018
Jul 2018
Aug 2018
Sep 2018
Oct 2018
Nov 2018
Dec 2018
Jan 2019
B
Station
VDM−AR−S1
VDM−AR−S2
VDM−AR−S3
VDM−AR−S4
IRL−AR−S9
IRL−AR−S8
IRL−AR−S7
IRL−AR−S6
IRL−AR−S5
IRL−AR−S3
BON−AR−S1
BON−AR−S2
BON−AR−S3
BON−AR−S4
BON−W−S5
BON−W−S6

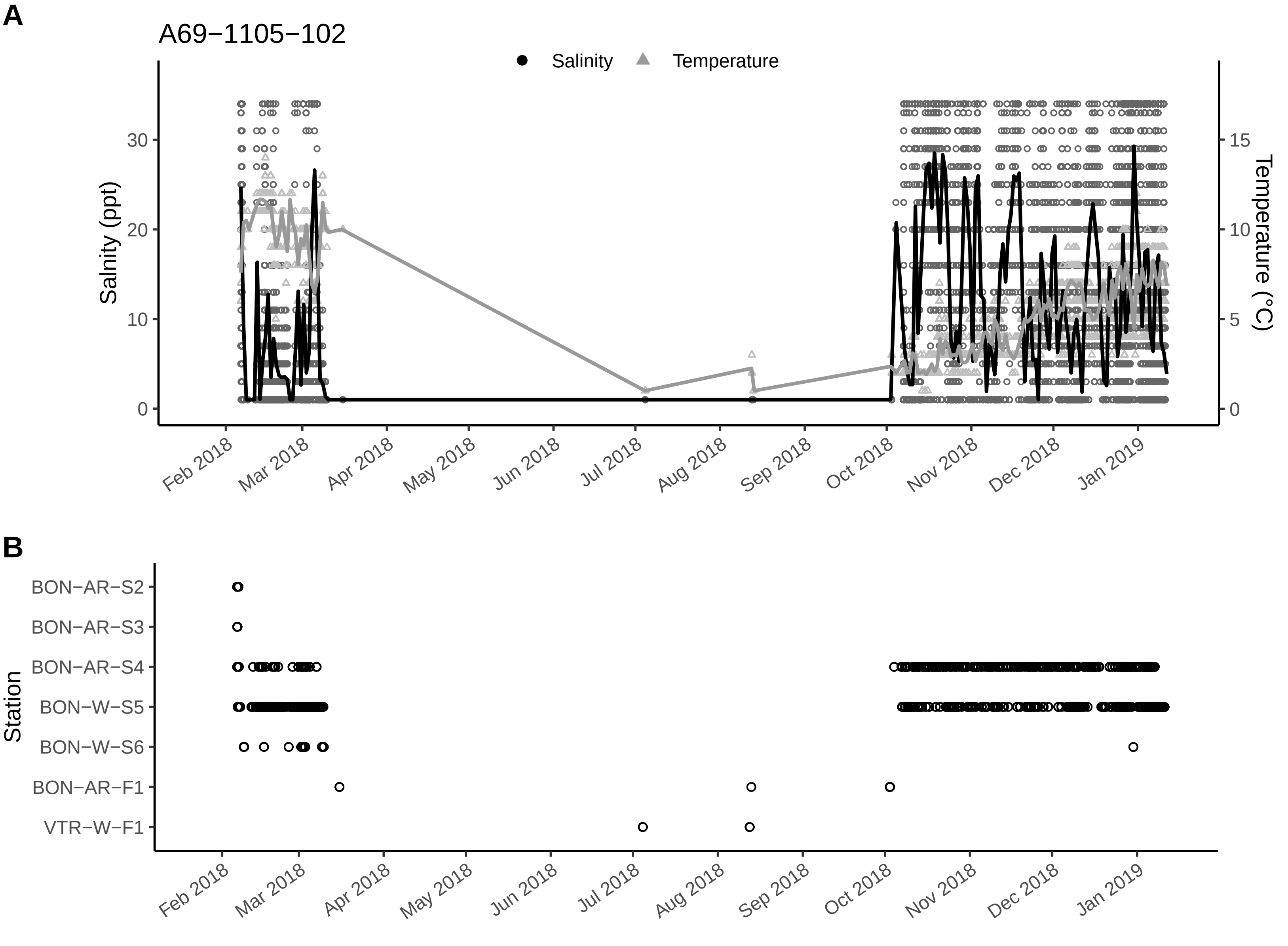

A
A69−1105−102
Salinity
Temperature
Salinity (ppt)
Temperature (°C)
0
10
20
30
0
5
10
15
Feb 2018
Mar 2018
Apr 2018
May 2018
Jun 2018
Jul 2018
Aug 2018
Sep 2018
Oct 2018
Nov 2018
Dec 2018
Jan 2019
B
Station
BON−AR−S2
BON−AR−S3
BON−AR−S4
BON−W−S5
BON−W−S6
BON−AR−F1
VTR−W−F1
Feb 2018
Mar 2018
Apr 2018
May 2018
Jun 2018
Jul 2018
Aug 2018
Sep 2018
Oct 2018
Nov 2018
Dec 2018
Jan 2019

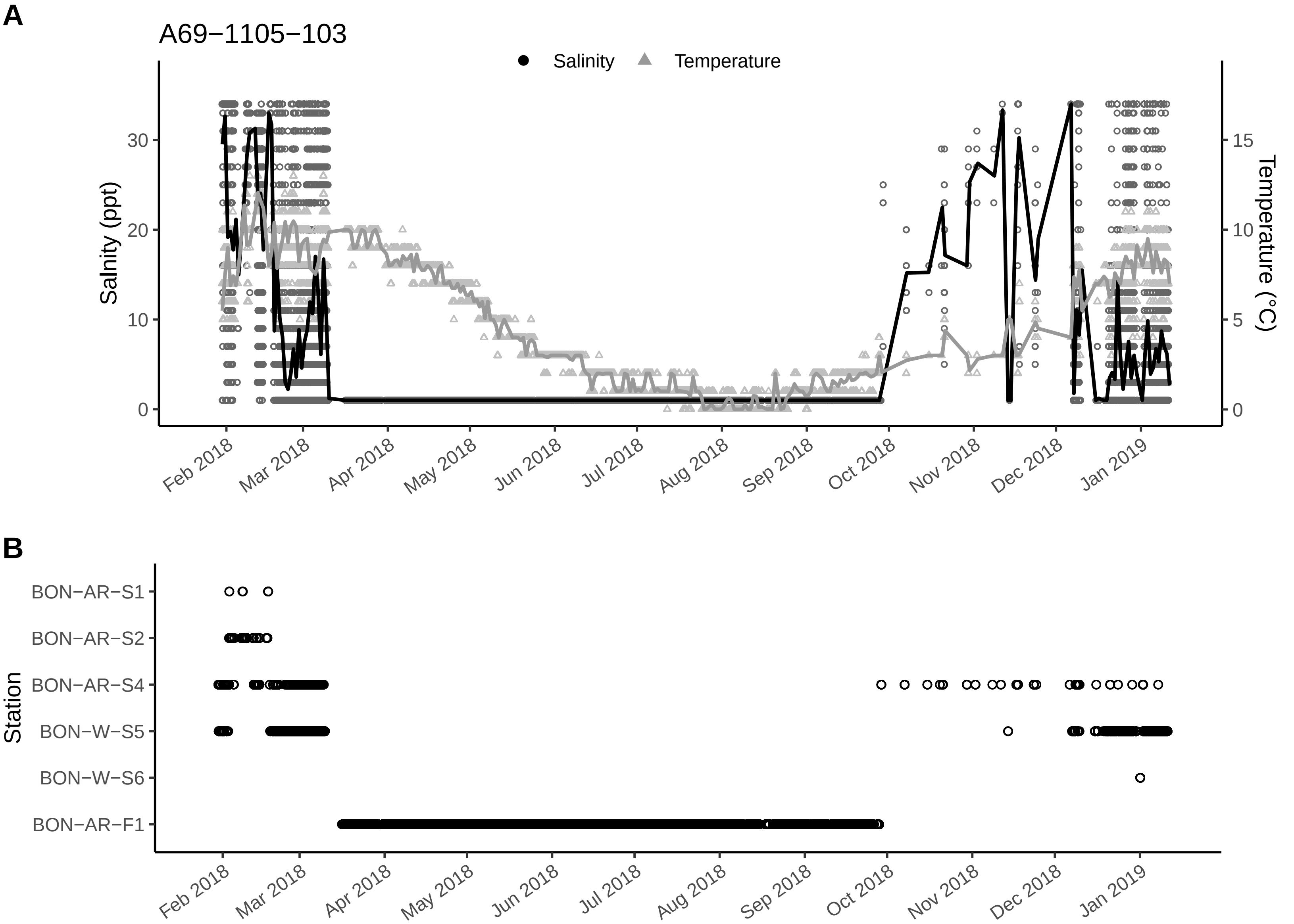
A
A69−1105−103
Salinity
Temperature
Salinity (ppt)
Temperature (°C)
0
10
20
30
0
5
10
15
Feb 2018
Mar 2018
Apr 2018
May 2018
Jun 2018
Jul 2018
Aug 2018
Sep 2018
Oct 2018
Nov 2018
Dec 2018
Jan 2019
B
Station
BON−AR−S1
BON−AR−S2
BON−AR−S4
BON−W−S5
BON−W−S6
BON−AR−F1
Feb 2018
Mar 2018
Apr 2018
May 2018
Jun 2018
Jul 2018
Aug 2018
Sep 2018
Oct 2018
Nov 2018
Dec 2018
Jan 2019

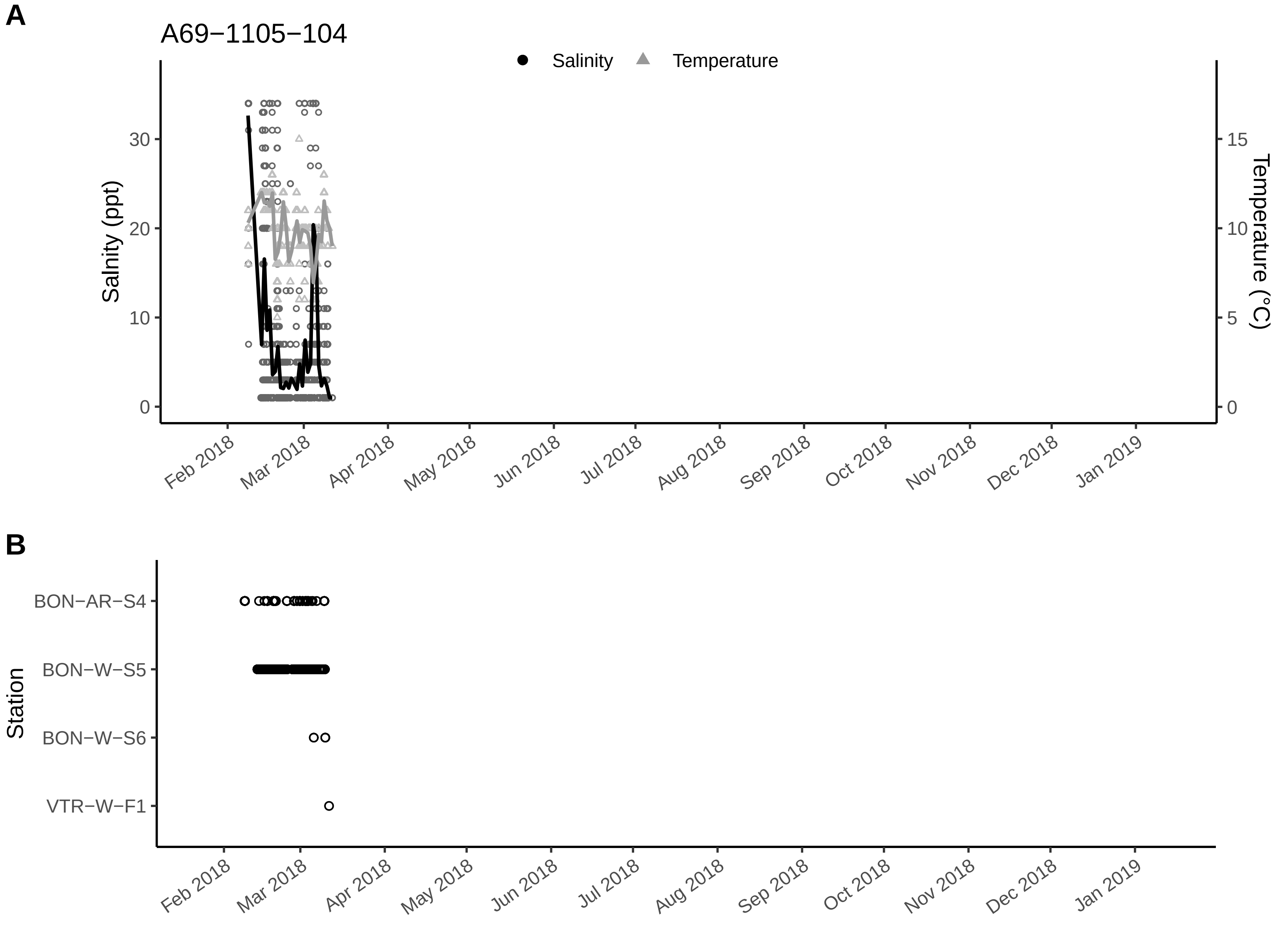

A
A69−1105−104
Salinity
Temperature
Salnity (ppt)
Temperature (°C)
0
10
20
30
0
5
10
15
Feb 2018
Mar 2018
Apr 2018
May 2018
Jun 2018
Jul 2018
Aug 2018
Sep 2018
Oct 2018
Nov 2018
Dec 2018
Jan 2019
B
Station
BON−AR−S4
BON−W−S5
BON−W−S6
VTR−W−F1
Feb 2018
Mar 2018
Apr 2018
May 2018
Jun 2018
Jul 2018
Aug 2018
Sep 2018
Oct 2018
Nov 2018
Dec 2018
Jan 2019

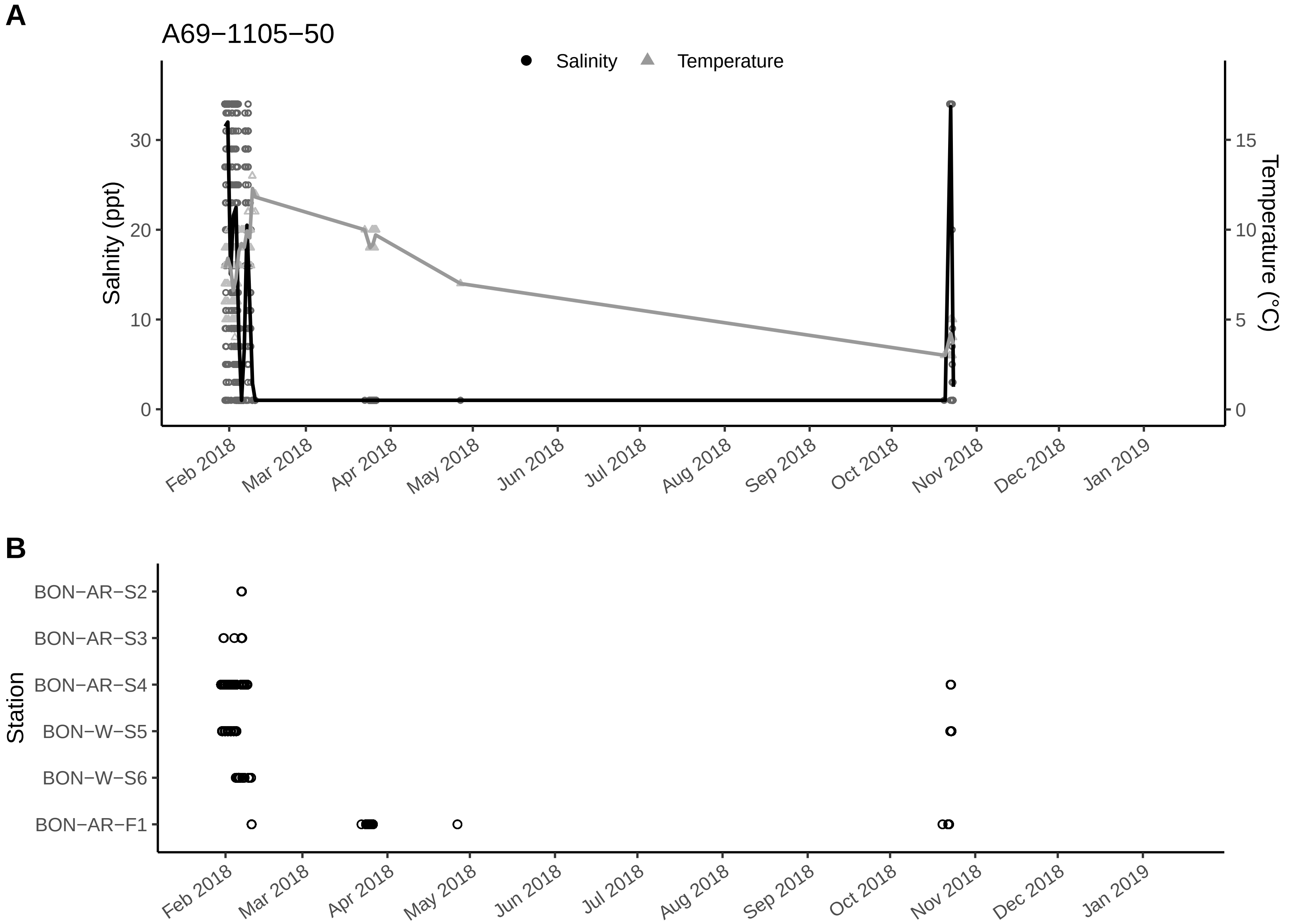
A
A69−1105−50
Salinity
Temperature
Salnity (ppt)
Temperature (°C)
0
10
20
30
0
5
10
15
Feb 2018
Mar 2018
Apr 2018
May 2018
Jun 2018
Jul 2018
Aug 2018
Sep 2018
Oct 2018
Nov 2018
Dec 2018
Jan 2019
B
Station
BON−AR−S2
BON−AR−S3
BON−AR−S4
BON−W−S5
BON−W−S6
BON−AR−F1
Feb 2018
Mar 2018
Apr 2018
May 2018
Jun 2018
Jul 2018
Aug 2018
Sep 2018
Oct 2018
Nov 2018
Dec 2018
Jan 2019

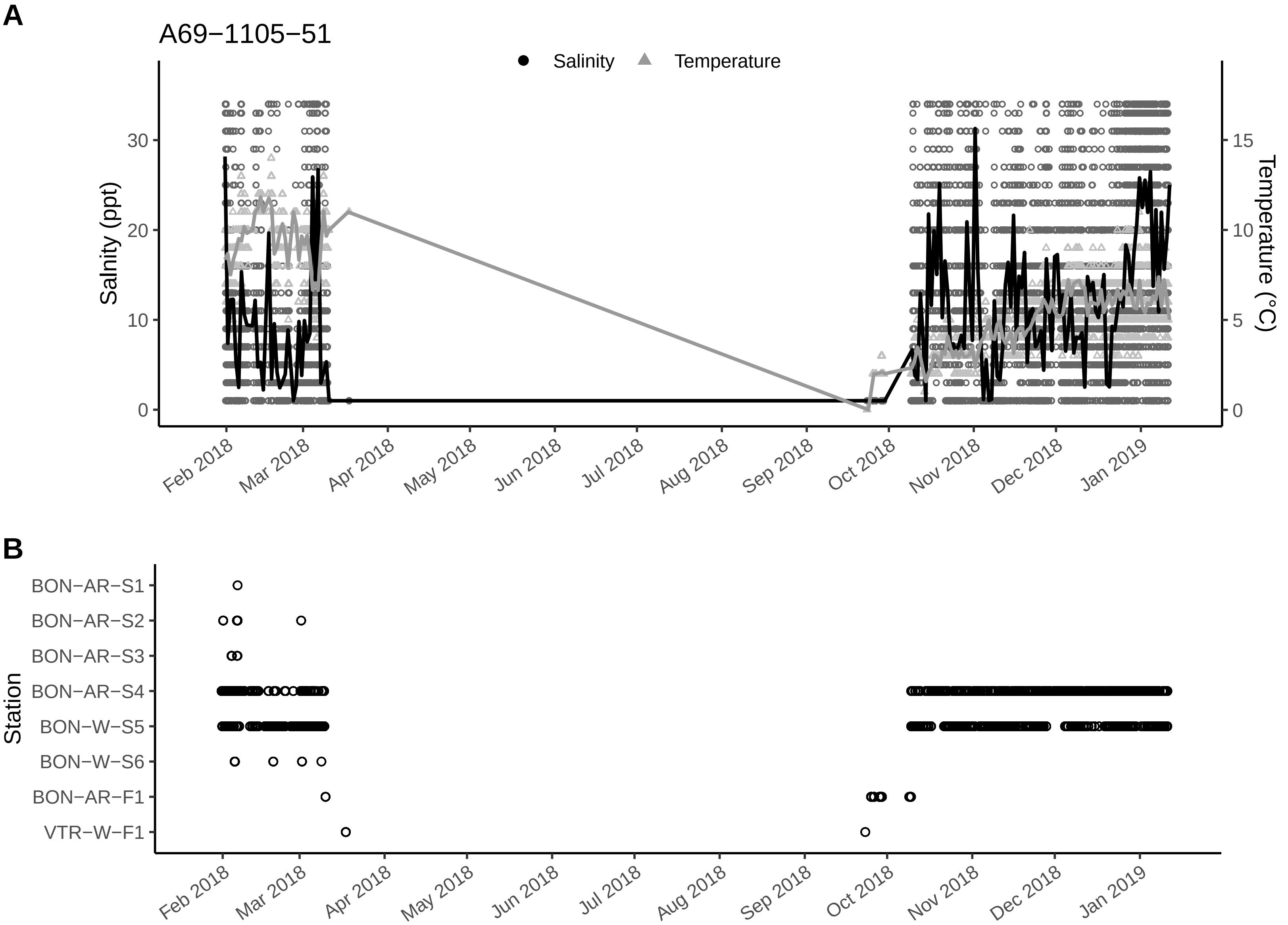
A
A69−1105−51
Salinity
Temperature
Salinity (ppt)
Temperature (°C)
0
10
20
30
0
5
10
15
Feb 2018
Mar 2018
Apr 2018
May 2018
Jun 2018
Jul 2018
Aug 2018
Sep 2018
Oct 2018
Nov 2018
Dec 2018
Jan 2019
B
Station
BON−AR−S1
BON−AR−S2
BON−AR−S3
BON−AR−S4
BON−W−S5
BON−W−S6
BON−AR−F1
VTR−W−F1
Feb 2018
Mar 2018
Apr 2018
May 2018
Jun 2018
Jul 2018
Aug 2018
Sep 2018
Oct 2018
Nov 2018
Dec 2018
Jan 2019

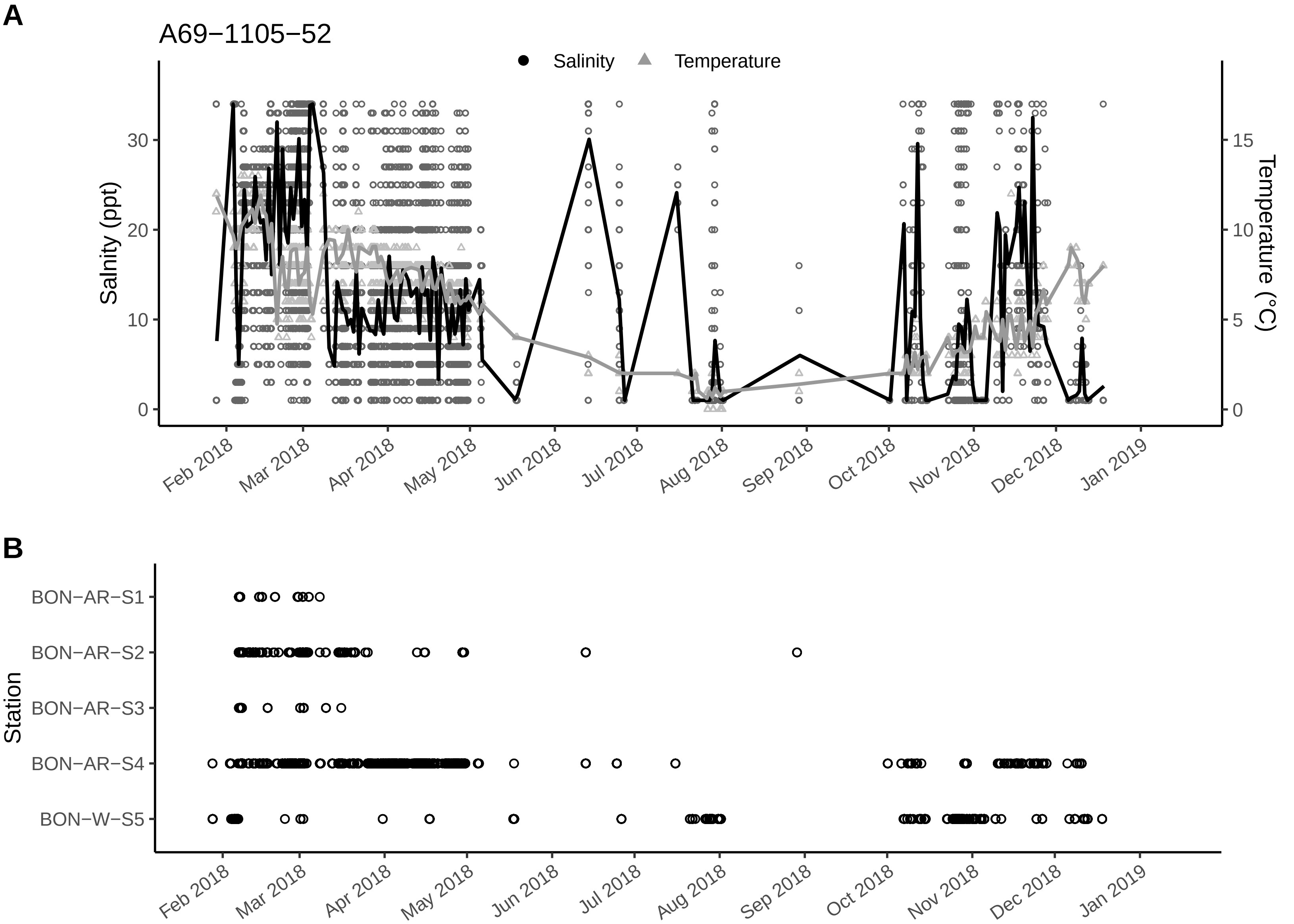
A
A69−1105−52
Salinity
Temperature
Salinity (ppt)
Temperature (°C)
0
10
20
30
0
5
10
15
Feb 2018
Mar 2018
Apr 2018
May 2018
Jun 2018
Jul 2018
Aug 2018
Sep 2018
Oct 2018
Nov 2018
Dec 2018
Jan 2019
B
Station
BON−AR−S1
BON−AR−S2
BON−AR−S3
BON−AR−S4
BON−W−S5

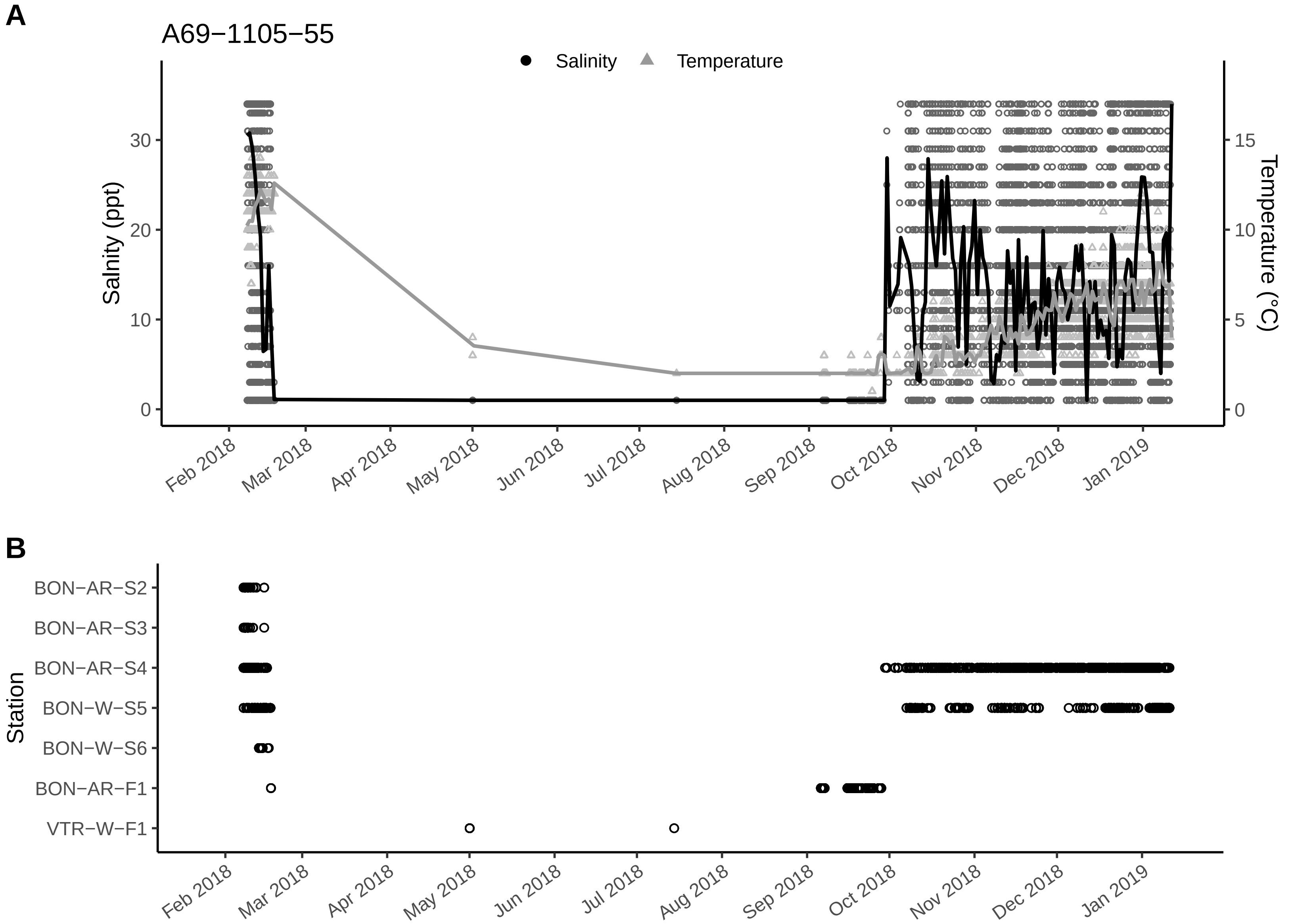
A
A69−1105−55
Salinity
Temperature
Salnity (ppt)
Temperature (°C)
0
10
20
30
0
5
10
15
Feb 2018
Mar 2018
Apr 2018
May 2018
Jun 2018
Jul 2018
Aug 2018
Sep 2018
Oct 2018
Nov 2018
Dec 2018
Jan 2019
B
Station
BON−AR−S2
BON−AR−S3
BON−AR−S4
BON−W−S5
BON−W−S6
BON−AR−F1
VTR−W−F1
Feb 2018
Mar 2018
Apr 2018
May 2018
Jun 2018
Jul 2018
Aug 2018
Sep 2018
Oct 2018
Nov 2018
Dec 2018
Jan 2019

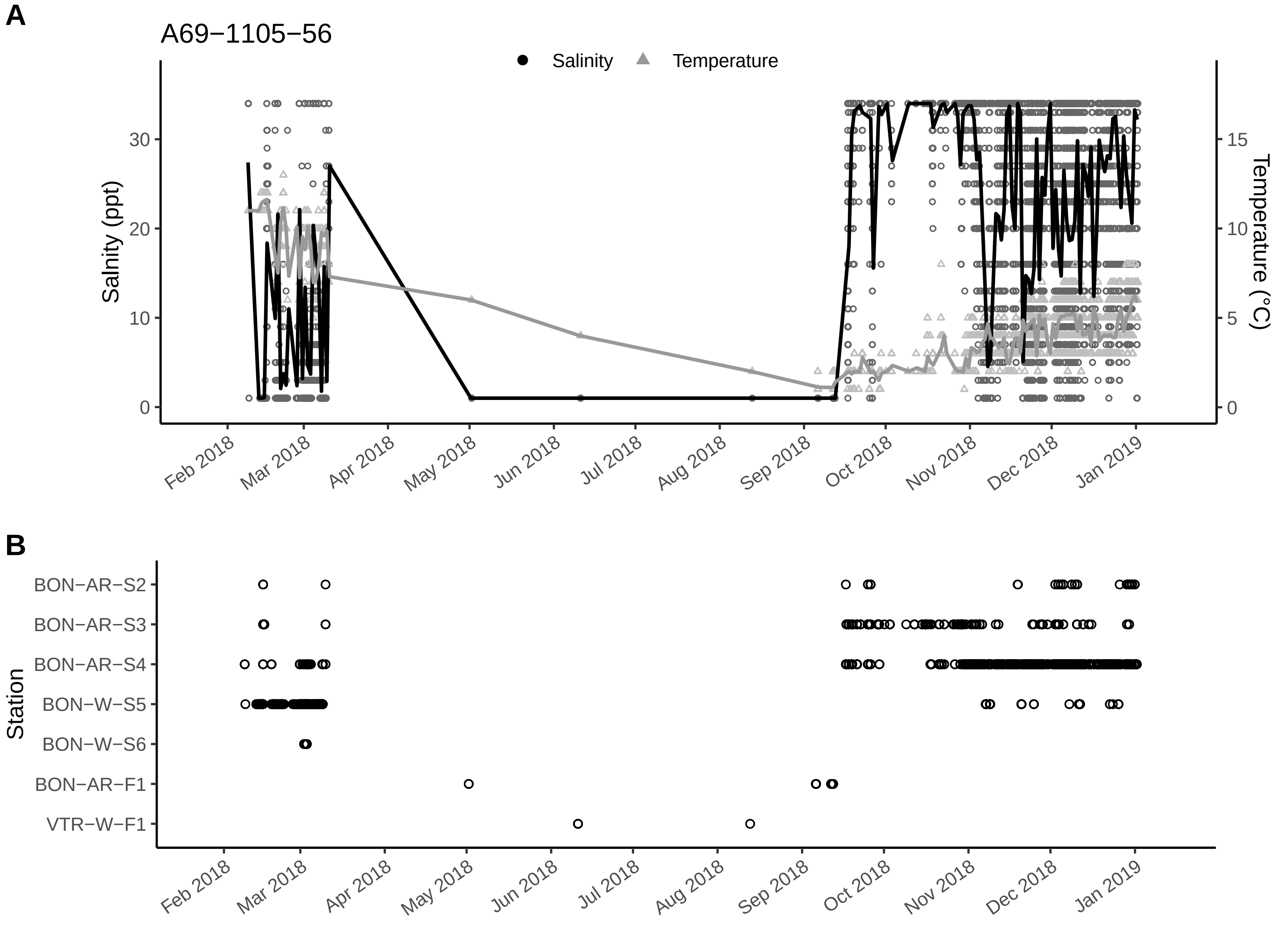
A
A69−1105−56
Salinity
Temperature
Salinity (ppt)
Temperature (°C)
0
10
20
30
0
5
10
15
Feb 2018
Mar 2018
Apr 2018
May 2018
Jun 2018
Jul 2018
Aug 2018
Sep 2018
Oct 2018
Nov 2018
Dec 2018
Jan 2019
B
Station
BON−AR−S2
BON−AR−S3
BON−AR−S4
BON−W−S5
BON−W−S6
BON−AR−F1
VTR−W−F1
Feb 2018
Mar 2018
Apr 2018
May 2018
Jun 2018
Jul 2018
Aug 2018
Sep 2018
Oct 2018
Nov 2018
Dec 2018
Jan 2019

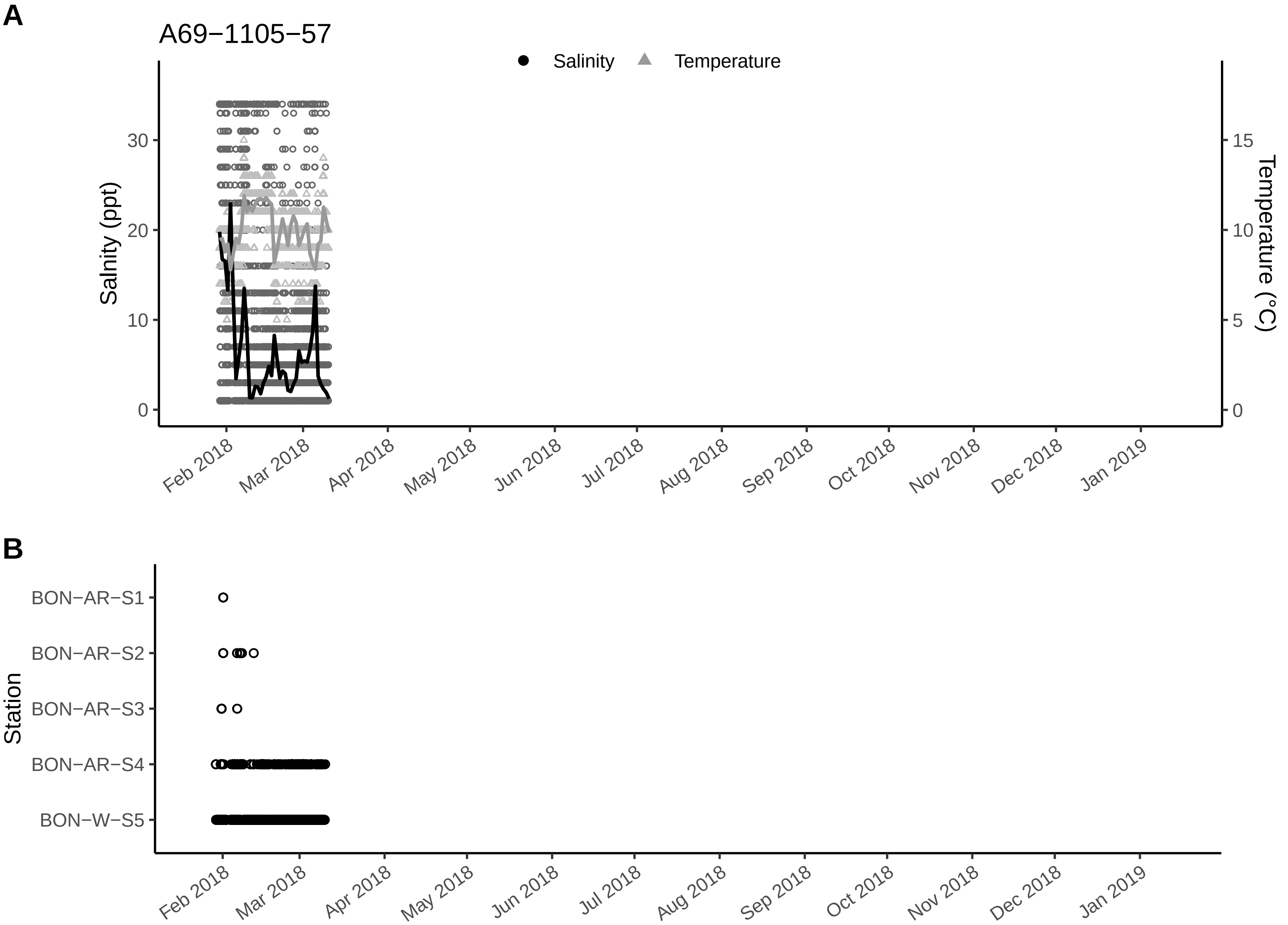

A
A69−1105−57
Salinity
Temperature
Salnity (ppt)
Temperature (°C)
0
10
20
30
0
5
10
15
Feb 2018
Mar 2018
Apr 2018
May 2018
Jun 2018
Jul 2018
Aug 2018
Sep 2018
Oct 2018
Nov 2018
Dec 2018
Jan 2019
B
Station
BON−AR−S1
BON−AR−S2
BON−AR−S3
BON−AR−S4
BON−W−S5
Feb 2018
Mar 2018
Apr 2018
May 2018
Jun 2018
Jul 2018
Aug 2018
Sep 2018
Oct 2018
Nov 2018
Dec 2018
Jan 2019

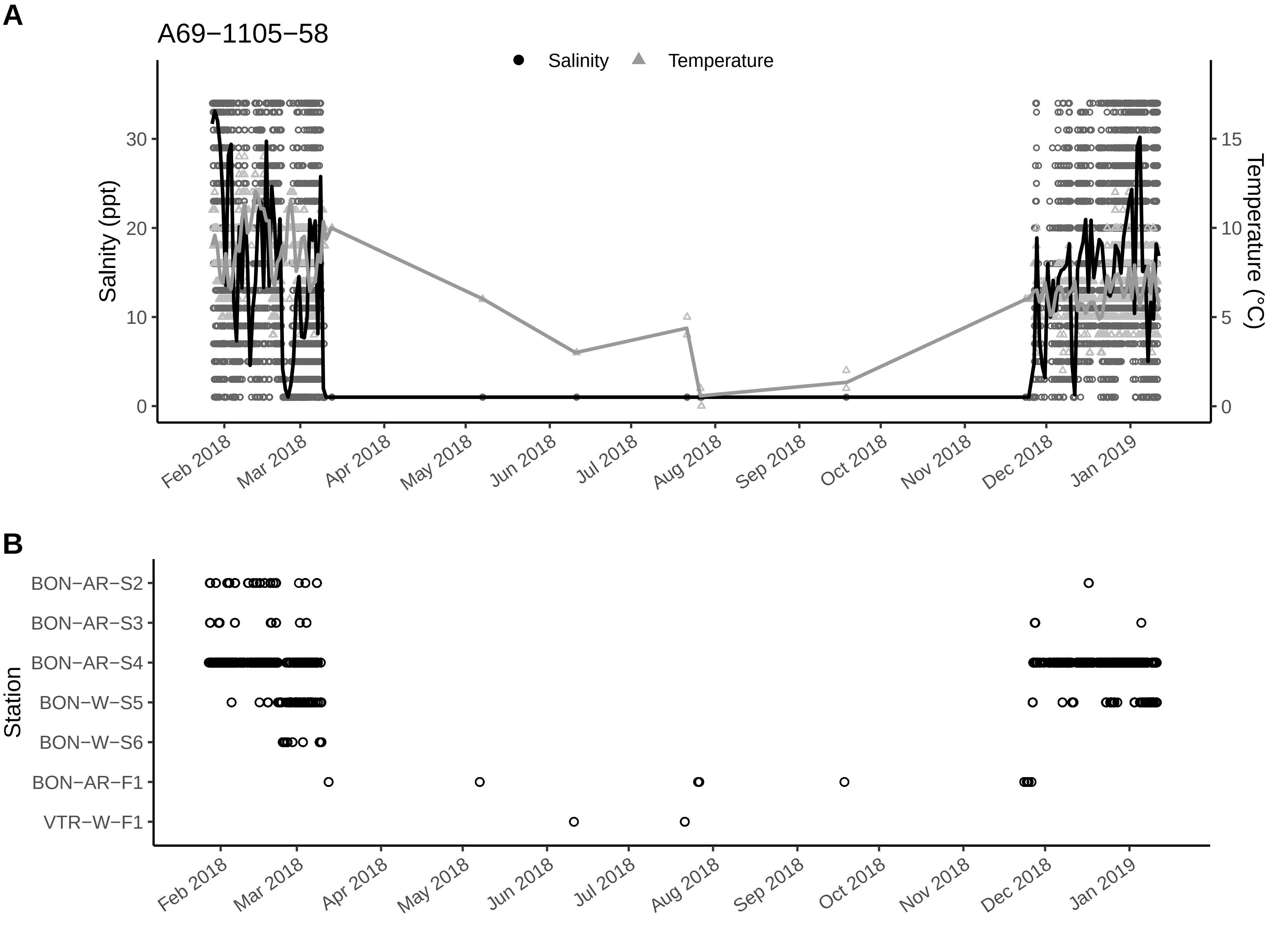
A
A69−1105−58
Salinity
Temperature
Salinity (ppt)
Temperature (°C)
0
10
20
30
0
5
10
15
Feb 2018
Mar 2018
Apr 2018
May 2018
Jun 2018
Jul 2018
Aug 2018
Sep 2018
Oct 2018
Nov 2018
Dec 2018
Jan 2019
B
Station
BON−AR−S2
BON−AR−S3
BON−AR−S4
BON−W−S5
BON−W−S6
BON−AR−F1
VTR−W−F1

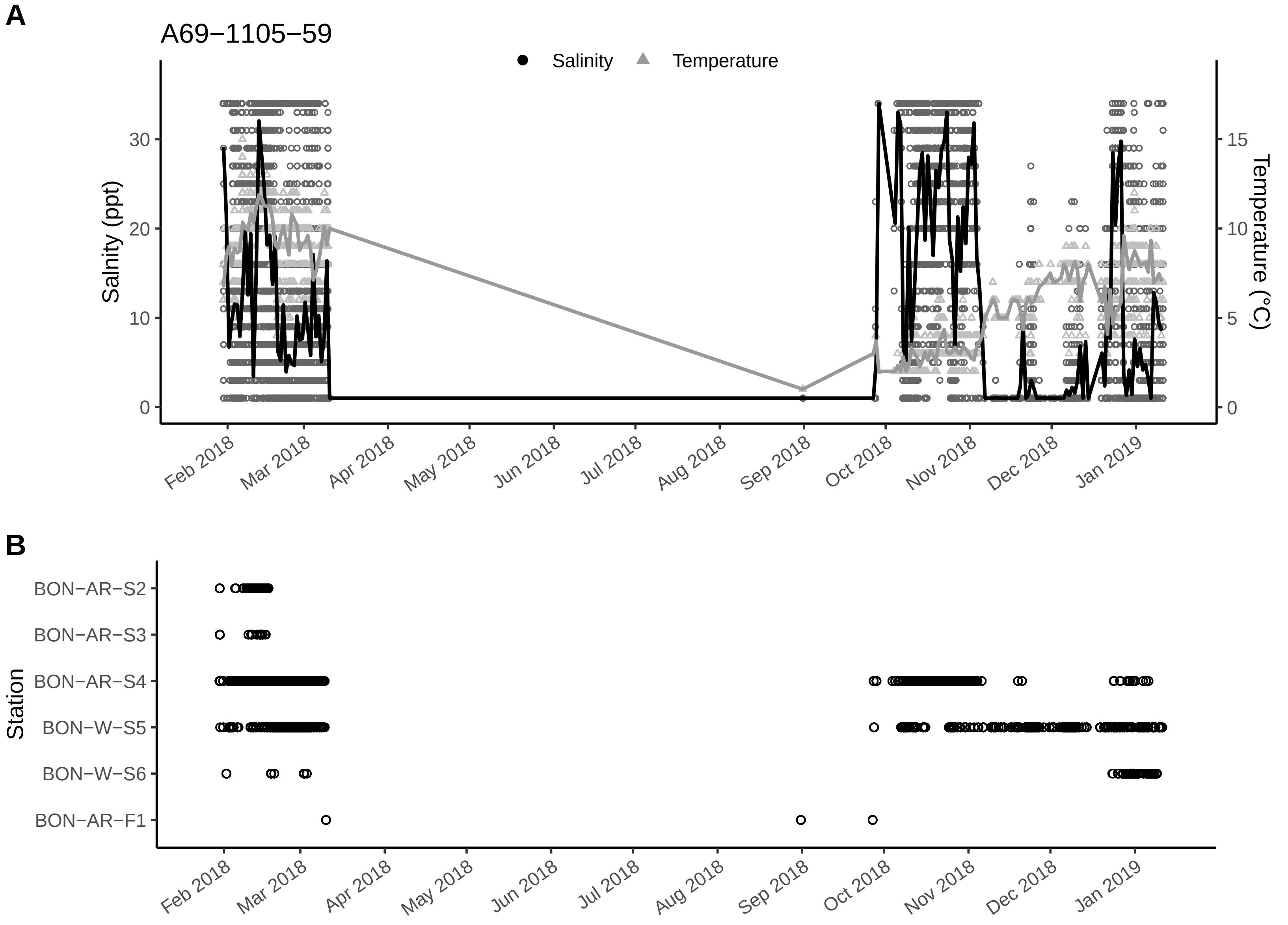
A
A69−1105−59
Salinity
Temperature
Salinity (ppt)
Temperature (°C)
Feb 2018
Mar 2018
Apr 2018
May 2018
Jun 2018
Jul 2018
Aug 2018
Sep 2018
Oct 2018
Nov 2018
Dec 2018
Jan 2019
B
Station
BON−AR−S2
BON−AR−S3
BON−AR−S4
BON−W−S5
BON−W−S6
BON−AR−F1

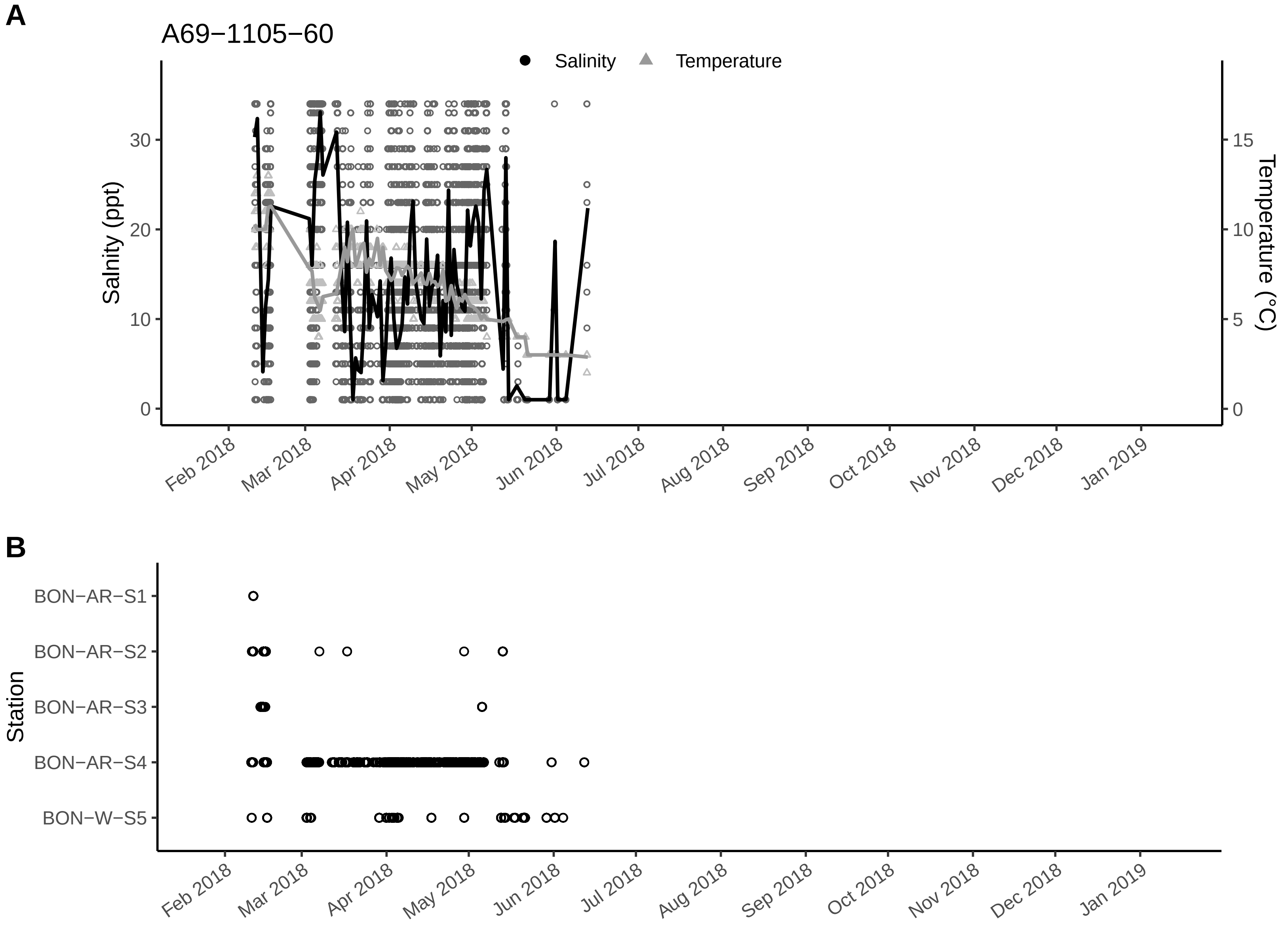
A
A69−1105−60
Salinity
Temperature
Salinity (ppt)
Temperature (°C)
Feb 2018
Mar 2018
Apr 2018
May 2018
Jun 2018
Jul 2018
Aug 2018
Sep 2018
Oct 2018
Nov 2018
Dec 2018
Jan 2019
B
Station
BON−AR−S1
BON−AR−S2
BON−AR−S3
BON−AR−S4
BON−W−S5

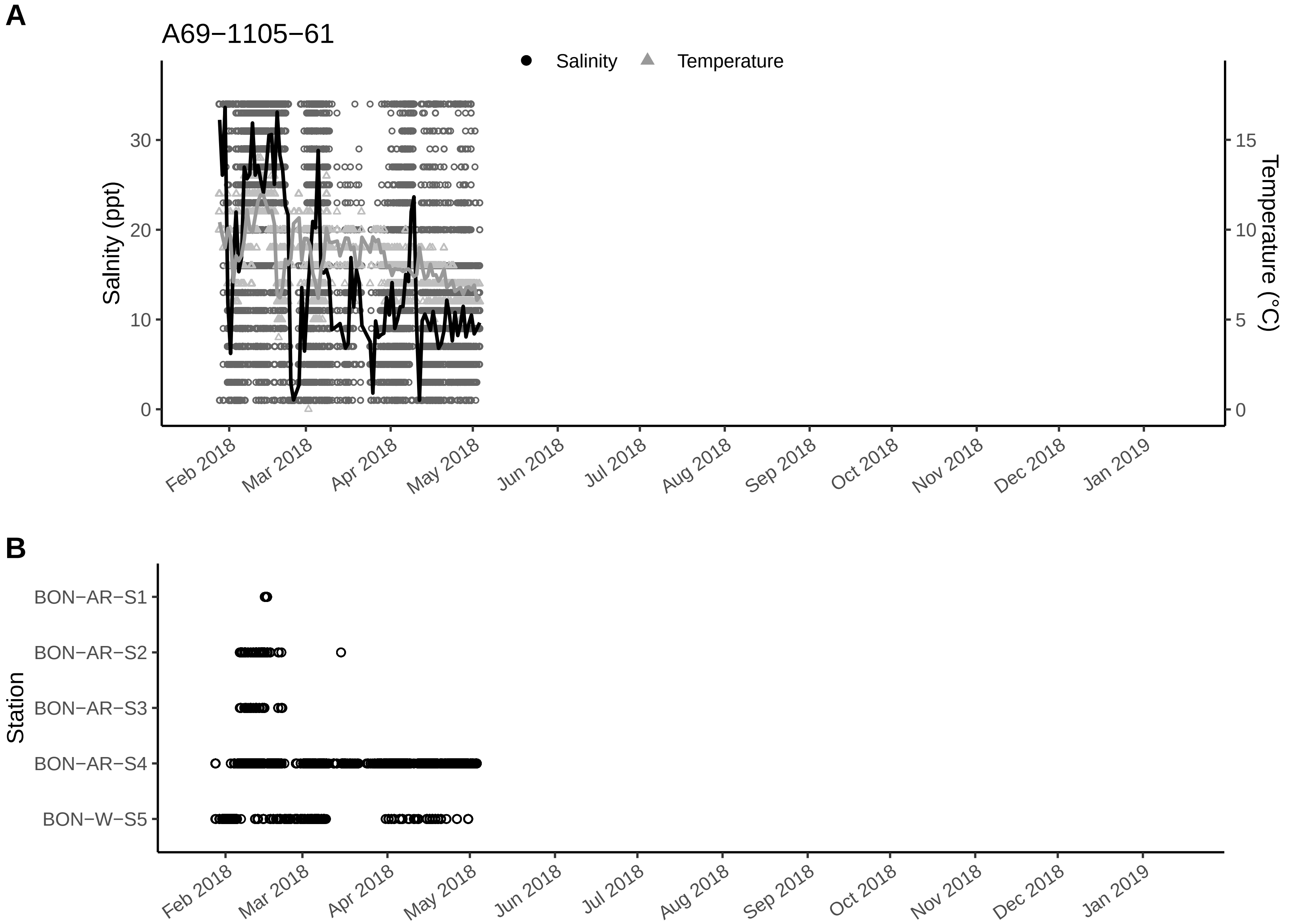

A
A69−1105−61
Salinity
Temperature
Salnity (ppt)
Temperature (°C)
0
10
20
30
0
5
10
15
Feb 2018
Mar 2018
Apr 2018
May 2018
Jun 2018
Jul 2018
Aug 2018
Sep 2018
Oct 2018
Nov 2018
Dec 2018
Jan 2019
B
Station
BON−AR−S1
BON−AR−S2
BON−AR−S3
BON−AR−S4
BON−W−S5
Feb 2018
Mar 2018
Apr 2018
May 2018
Jun 2018
Jul 2018
Aug 2018
Sep 2018
Oct 2018
Nov 2018
Dec 2018
Jan 2019

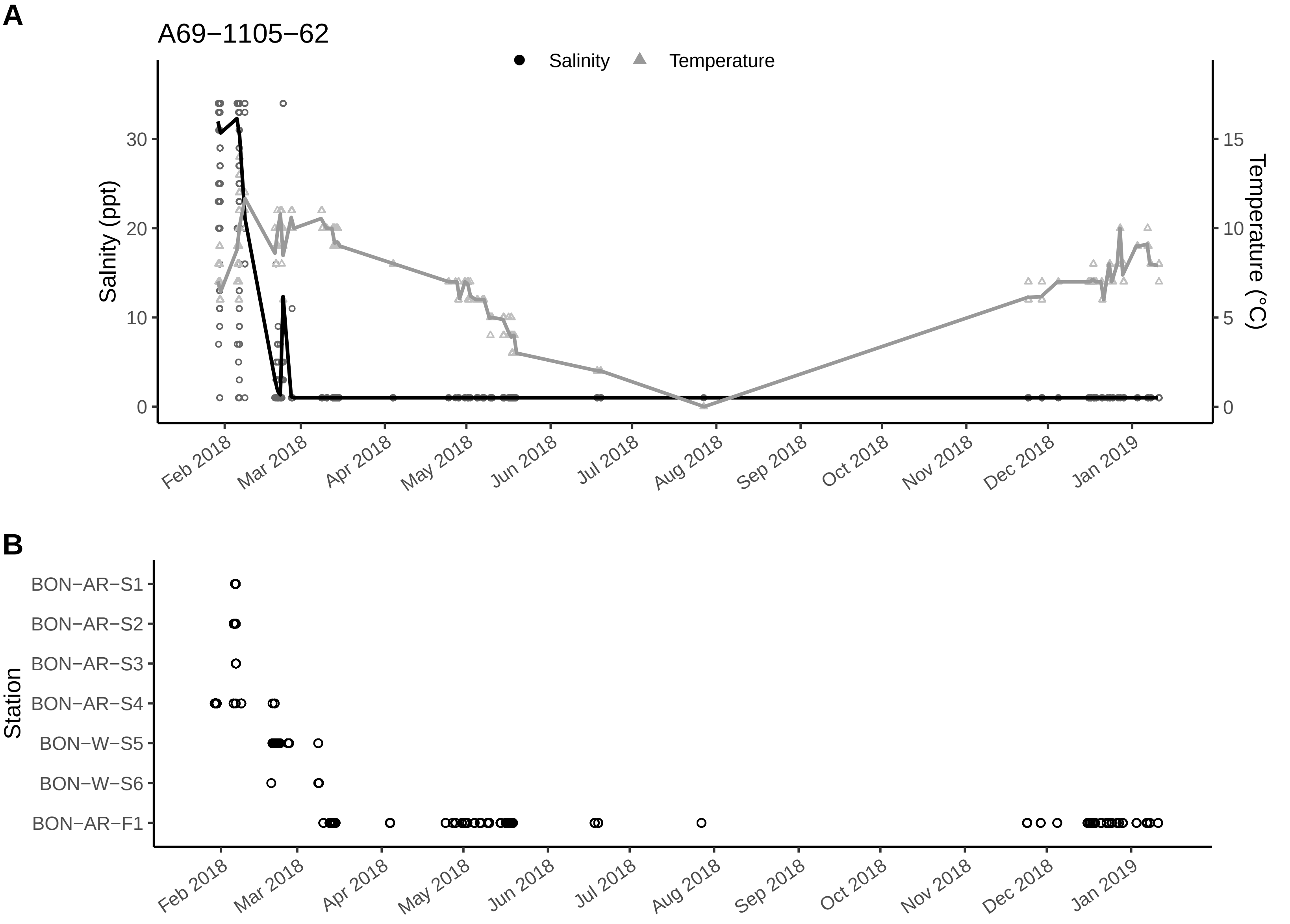
A
A69−1105−62
Salinity
Temperature
Salnity (ppt)
Temperature (°C)
0
10
20
30
0
5
10
15
Feb 2018
Mar 2018
Apr 2018
May 2018
Jun 2018
Jul 2018
Aug 2018
Sep 2018
Oct 2018
Nov 2018
Dec 2018
Jan 2019
B
Station
BON−AR−S1
BON−AR−S2
BON−AR−S3
BON−AR−S4
BON−W−S5
BON−W−S6
BON−AR−F1
Feb 2018
Mar 2018
Apr 2018
May 2018
Jun 2018
Jul 2018
Aug 2018
Sep 2018
Oct 2018
Nov 2018
Dec 2018
Jan 2019

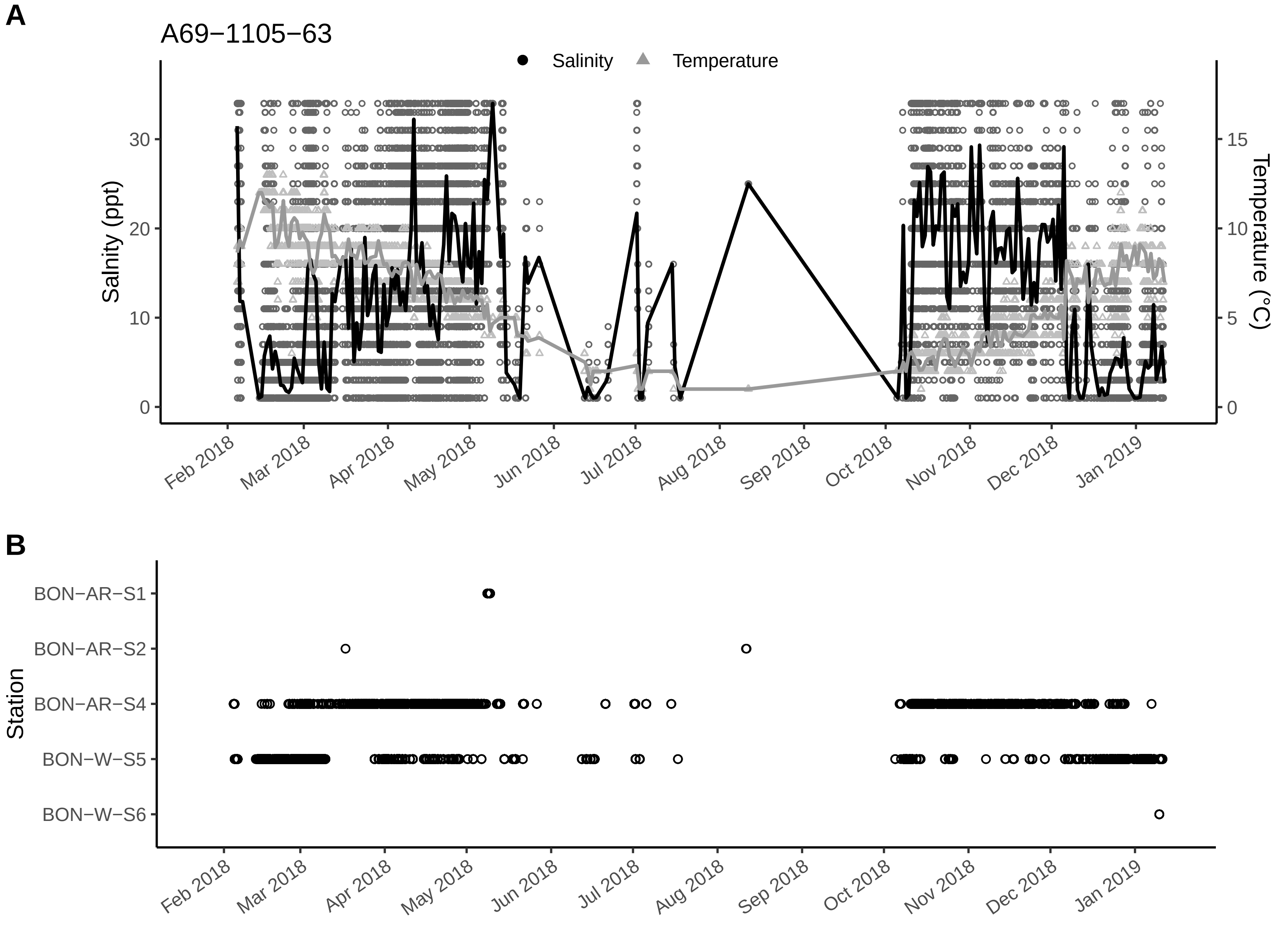
A
A69−1105−63
Salinity
Temperature
Salinity (ppt)
Temperature (°C)
0
10
20
30
0
5
10
15
Feb 2018
Mar 2018
Apr 2018
May 2018
Jun 2018
Jul 2018
Aug 2018
Sep 2018
Oct 2018
Nov 2018
Dec 2018
Jan 2019
B
Station
BON−AR−S1
BON−AR−S2
BON−AR−S4
BON−W−S5
BON−W−S6
Feb 2018
Mar 2018
Apr 2018
May 2018
Jun 2018
Jul 2018
Aug 2018
Sep 2018
Oct 2018
Nov 2018
Dec 2018
Jan 2019

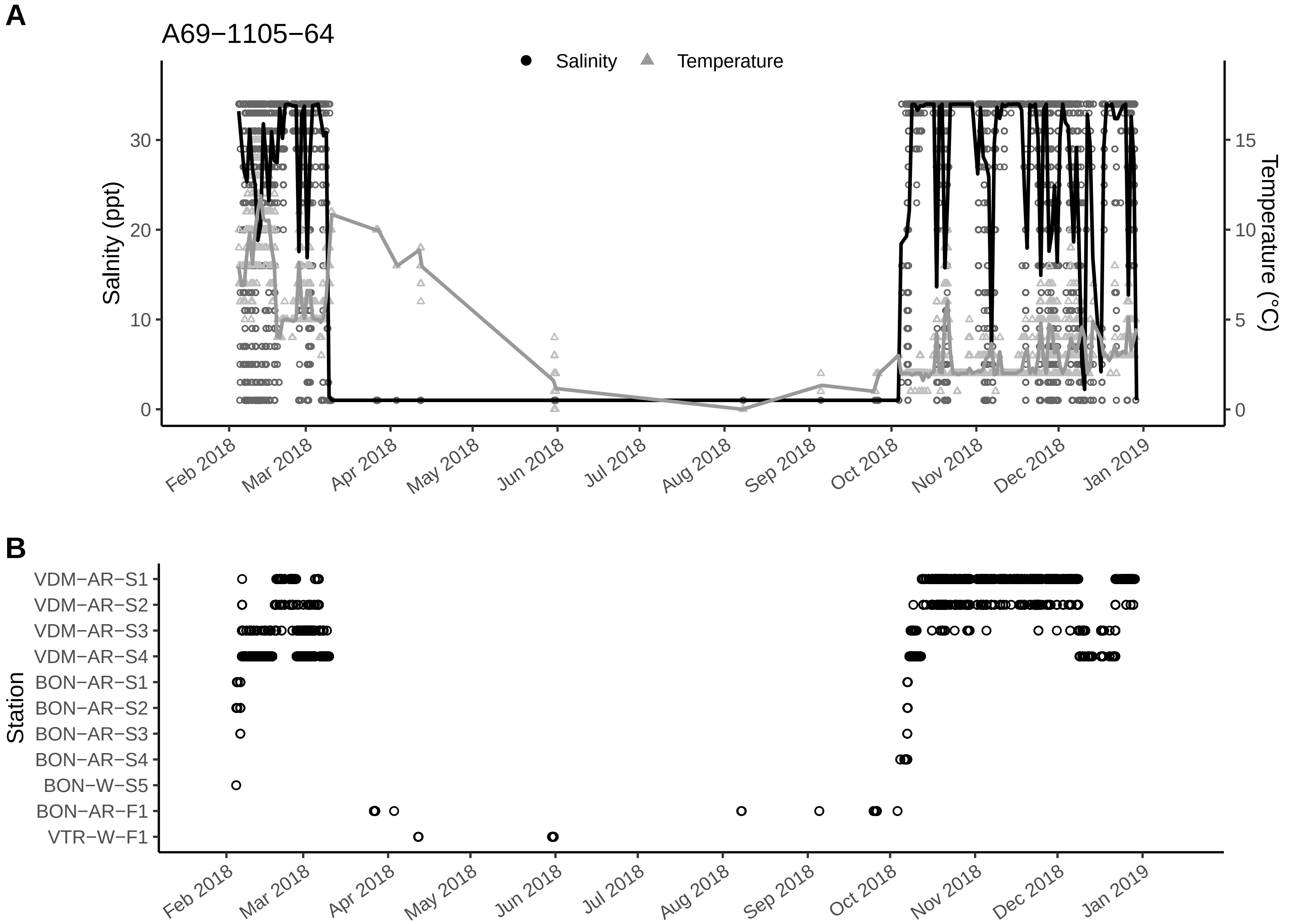
A
A69−1105−64
Salinity
Temperature
Salinity (ppt)
Temperature (°C)
0
10
20
30
0
5
10
15
Feb 2018
Mar 2018
Apr 2018
May 2018
Jun 2018
Jul 2018
Aug 2018
Sep 2018
Oct 2018
Nov 2018
Dec 2018
Jan 2019
B
Station
VDM−AR−S1
VDM−AR−S2
VDM−AR−S3
VDM−AR−S4
BON−AR−S1
BON−AR−S2
BON−AR−S3
BON−AR−S4
BON−W−S5
BON−AR−F1
VTR−W−F1
Feb 2018
Mar 2018
Apr 2018
May 2018
Jun 2018
Jul 2018
Aug 2018
Sep 2018
Oct 2018
Nov 2018
Dec 2018
Jan 2019

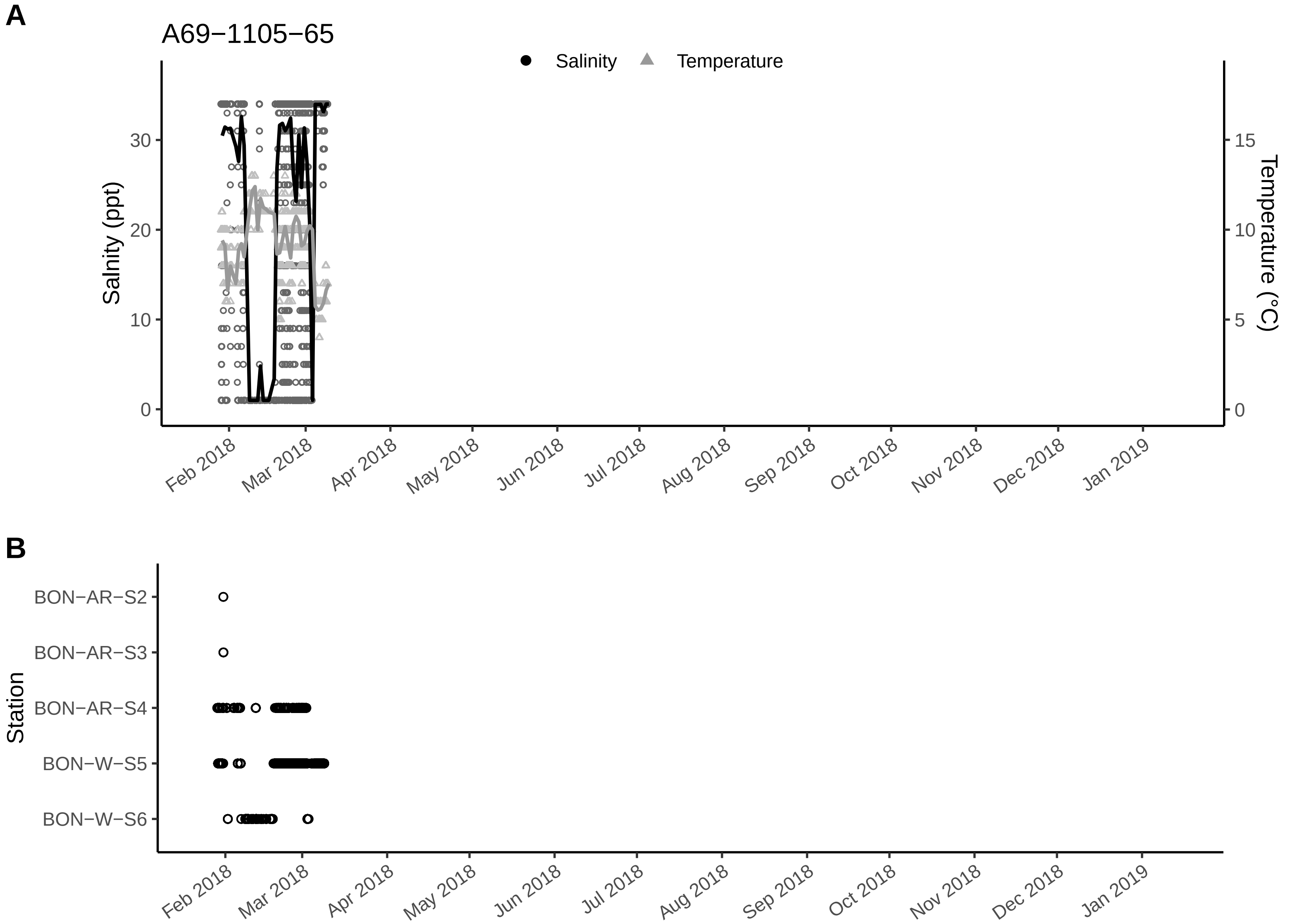
A
A69−1105−65
Salinity
Temperature
Salnity (ppt)
Temperature (°C)
0
10
20
30
0
5
10
15
Feb 2018
Mar 2018
Apr 2018
May 2018
Jun 2018
Jul 2018
Aug 2018
Sep 2018
Oct 2018
Nov 2018
Dec 2018
Jan 2019
B
Station
BON−AR−S2
BON−AR−S3
BON−AR−S4
BON−W−S5
BON−W−S6
Feb 2018
Mar 2018
Apr 2018
May 2018
Jun 2018
Jul 2018
Aug 2018
Sep 2018
Oct 2018
Nov 2018
Dec 2018
Jan 2019

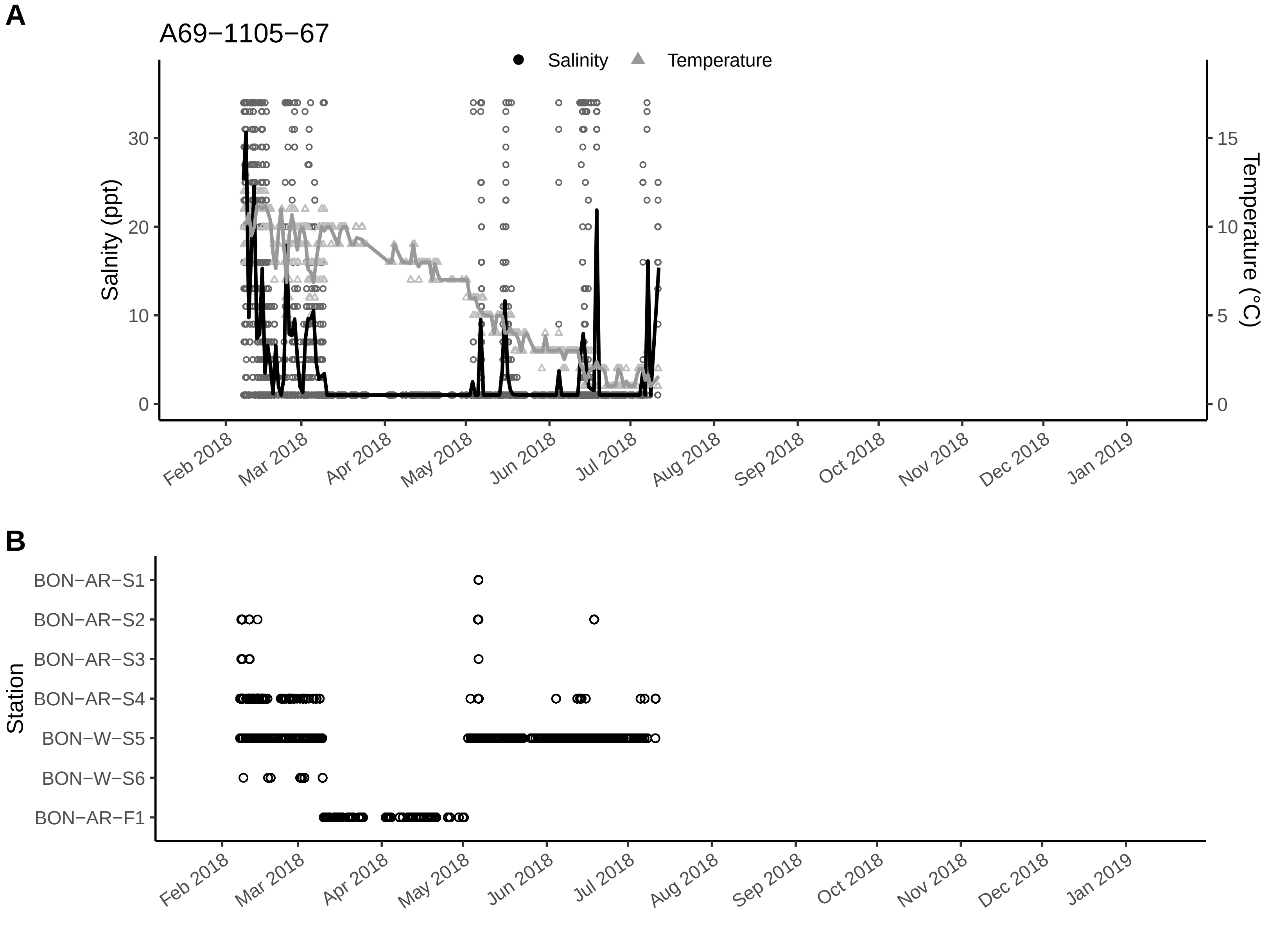
A
A69−1105−67
Salinity
Temperature
Salnity (ppt)
Temperature (°C)
0
10
20
30
0
5
10
15
Feb 2018
Mar 2018
Apr 2018
May 2018
Jun 2018
Jul 2018
Aug 2018
Sep 2018
Oct 2018
Nov 2018
Dec 2018
Jan 2019
B
Station
BON−AR−S1
BON−AR−S2
BON−AR−S3
BON−AR−S4
BON−W−S5
BON−W−S6
BON−AR−F1
Feb 2018
Mar 2018
Apr 2018
May 2018
Jun 2018
Jul 2018
Aug 2018
Sep 2018
Oct 2018
Nov 2018
Dec 2018
Jan 2019

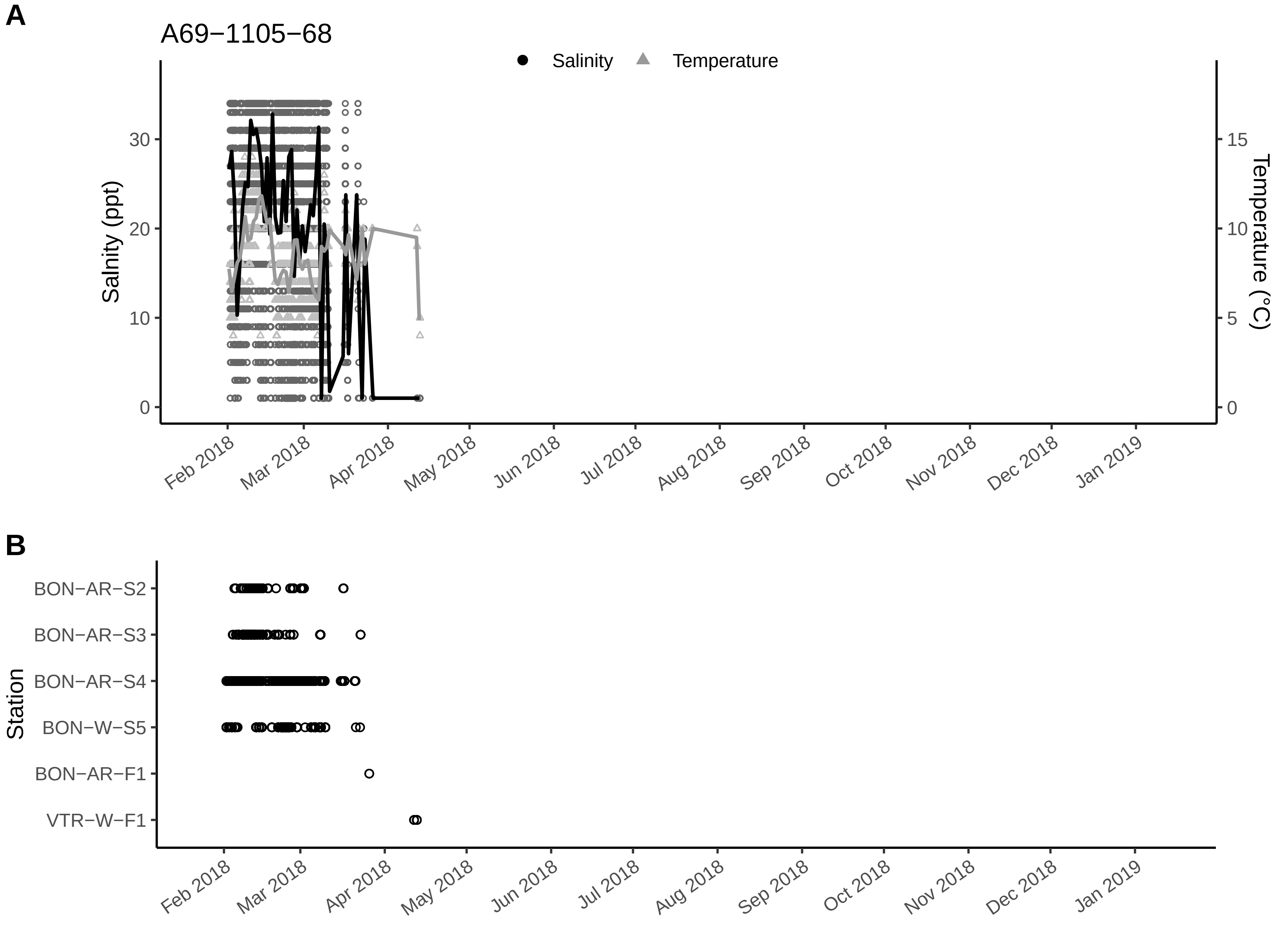
A
A69−1105−68
Salinity
Temperature
Salnity (ppt)
Temperature (°C)
0
10
20
30
0
5
10
15
Feb 2018
Mar 2018
Apr 2018
May 2018
Jun 2018
Jul 2018
Aug 2018
Sep 2018
Oct 2018
Nov 2018
Dec 2018
Jan 2019
B
Station
BON−AR−S2
BON−AR−S3
BON−AR−S4
BON−W−S5
BON−AR−F1
VTR−W−F1
Feb 2018
Mar 2018
Apr 2018
May 2018
Jun 2018
Jul 2018
Aug 2018
Sep 2018
Oct 2018
Nov 2018
Dec 2018
Jan 2019

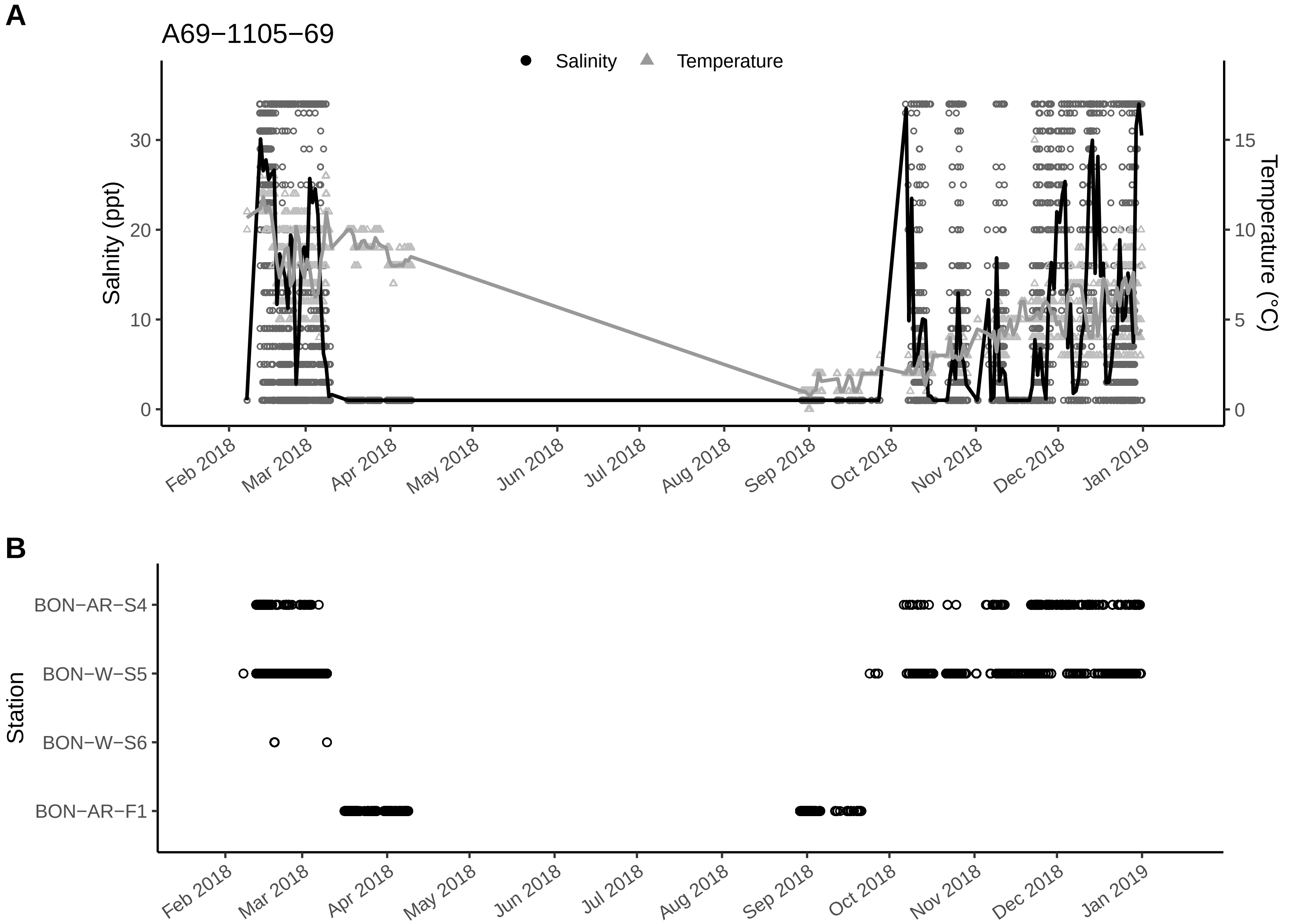
A
A69−1105−69
Salinity
Temperature
Salinity (ppt)
Temperature (°C)
0
10
20
30
0
5
10
15
Feb 2018
Mar 2018
Apr 2018
May 2018
Jun 2018
Jul 2018
Aug 2018
Sep 2018
Oct 2018
Nov 2018
Dec 2018
Jan 2019
B
Station
BON−AR−S4
BON−W−S5
BON−W−S6
BON−AR−F1
Feb 2018
Mar 2018
Apr 2018
May 2018
Jun 2018
Jul 2018
Aug 2018
Sep 2018
Oct 2018
Nov 2018
Dec 2018
Jan 2019

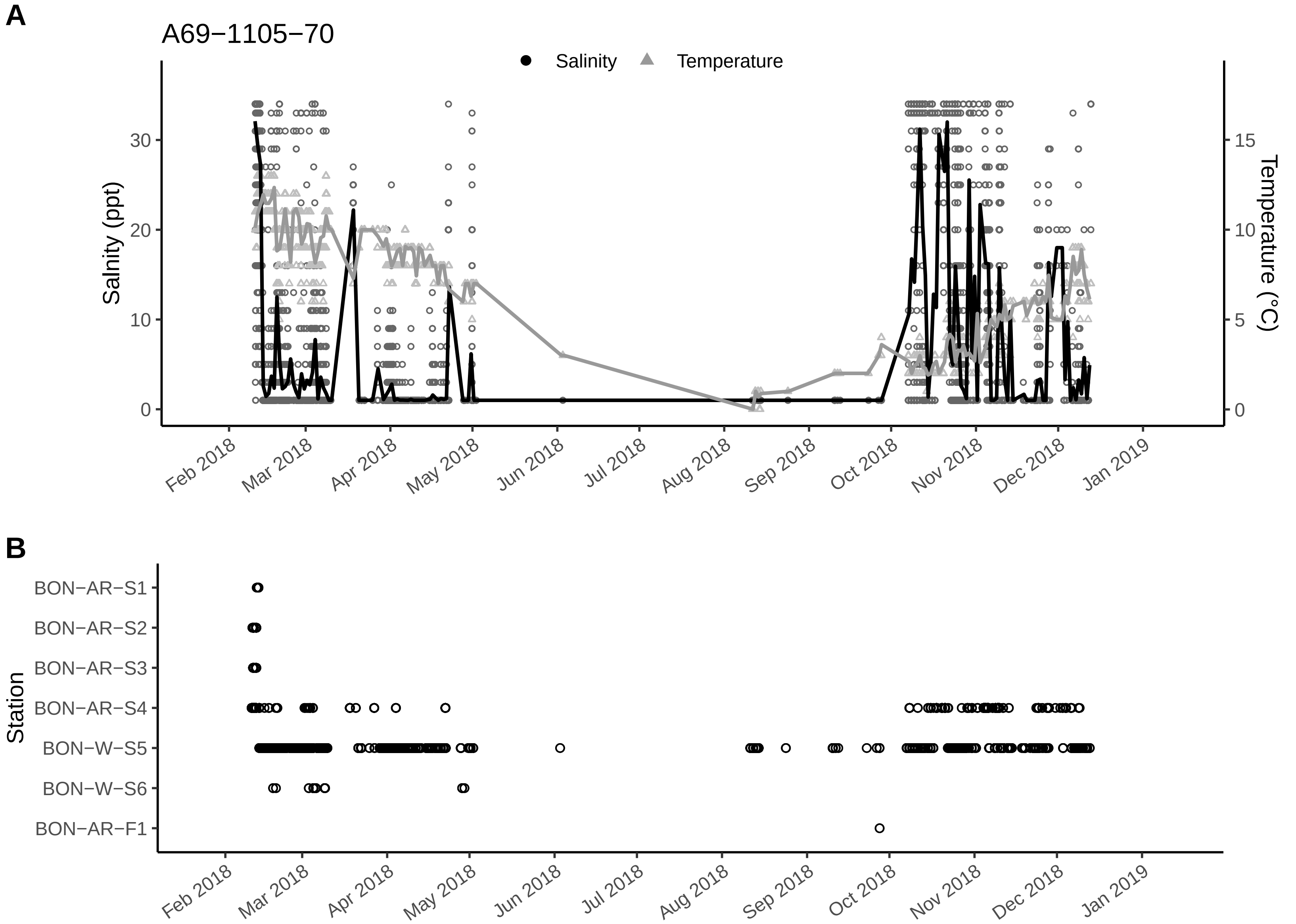
A
A69−1105−70
Salinity
Temperature
Salinity (ppt)
Temperature (°C)
Feb 2018
Mar 2018
Apr 2018
May 2018
Jun 2018
Jul 2018
Aug 2018
Sep 2018
Oct 2018
Nov 2018
Dec 2018
Jan 2019
B
Station
BON−AR−S1
BON−AR−S2
BON−AR−S3
BON−AR−S4
BON−W−S5
BON−W−S6
BON−AR−F1

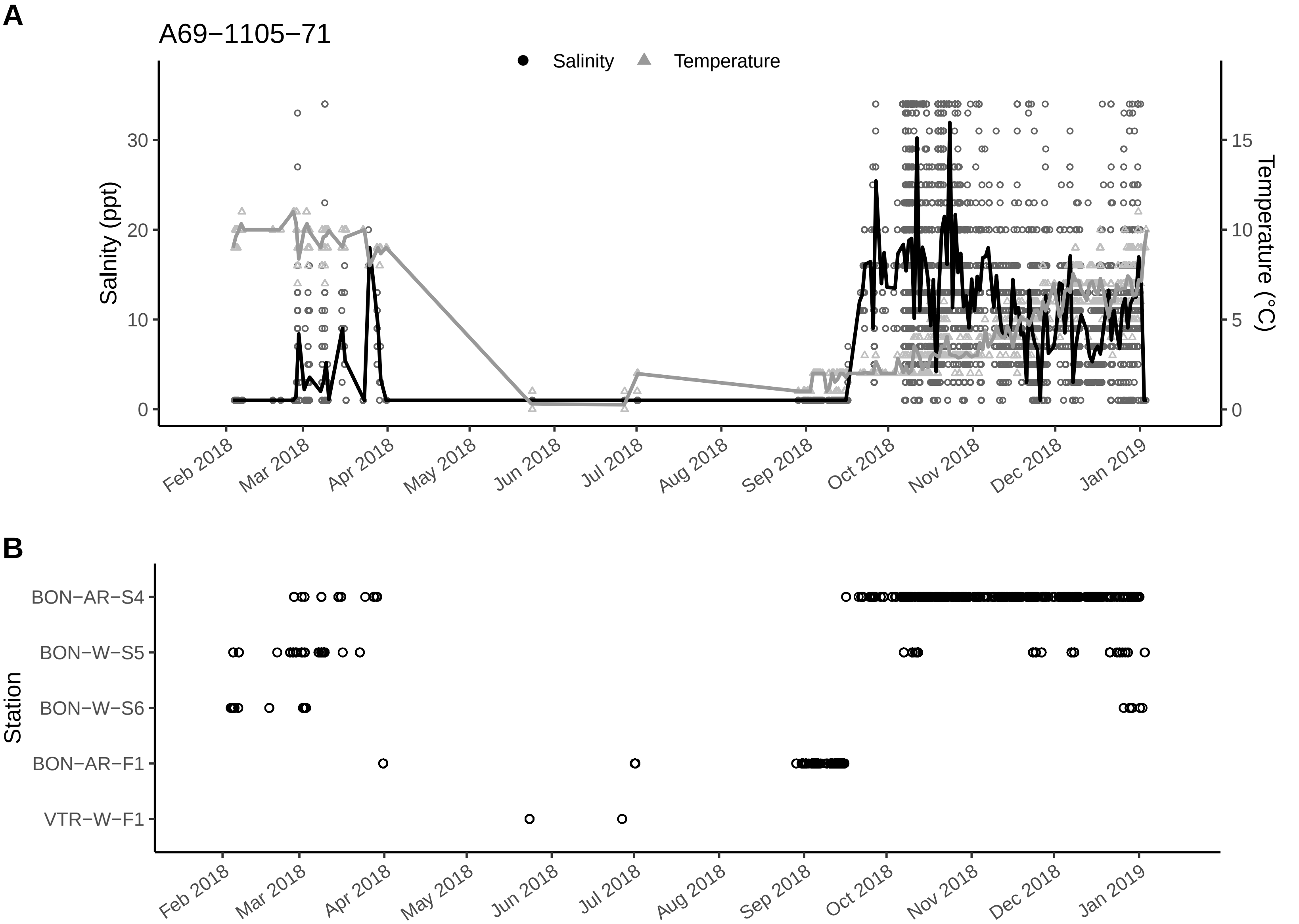
A
A69−1105−71
Salinity
Temperature
Salinity (ppt)
Temperature (°C)
0
10
20
30
0
5
10
15
Feb 2018
Mar 2018
Apr 2018
May 2018
Jun 2018
Jul 2018
Aug 2018
Sep 2018
Oct 2018
Nov 2018
Dec 2018
Jan 2019
B
Station
BON−AR−S4
BON−W−S5
BON−W−S6
BON−AR−F1
VTR−W−F1
Feb 2018
Mar 2018
Apr 2018
May 2018
Jun 2018
Jul 2018
Aug 2018
Sep 2018
Oct 2018
Nov 2018
Dec 2018
Jan 2019

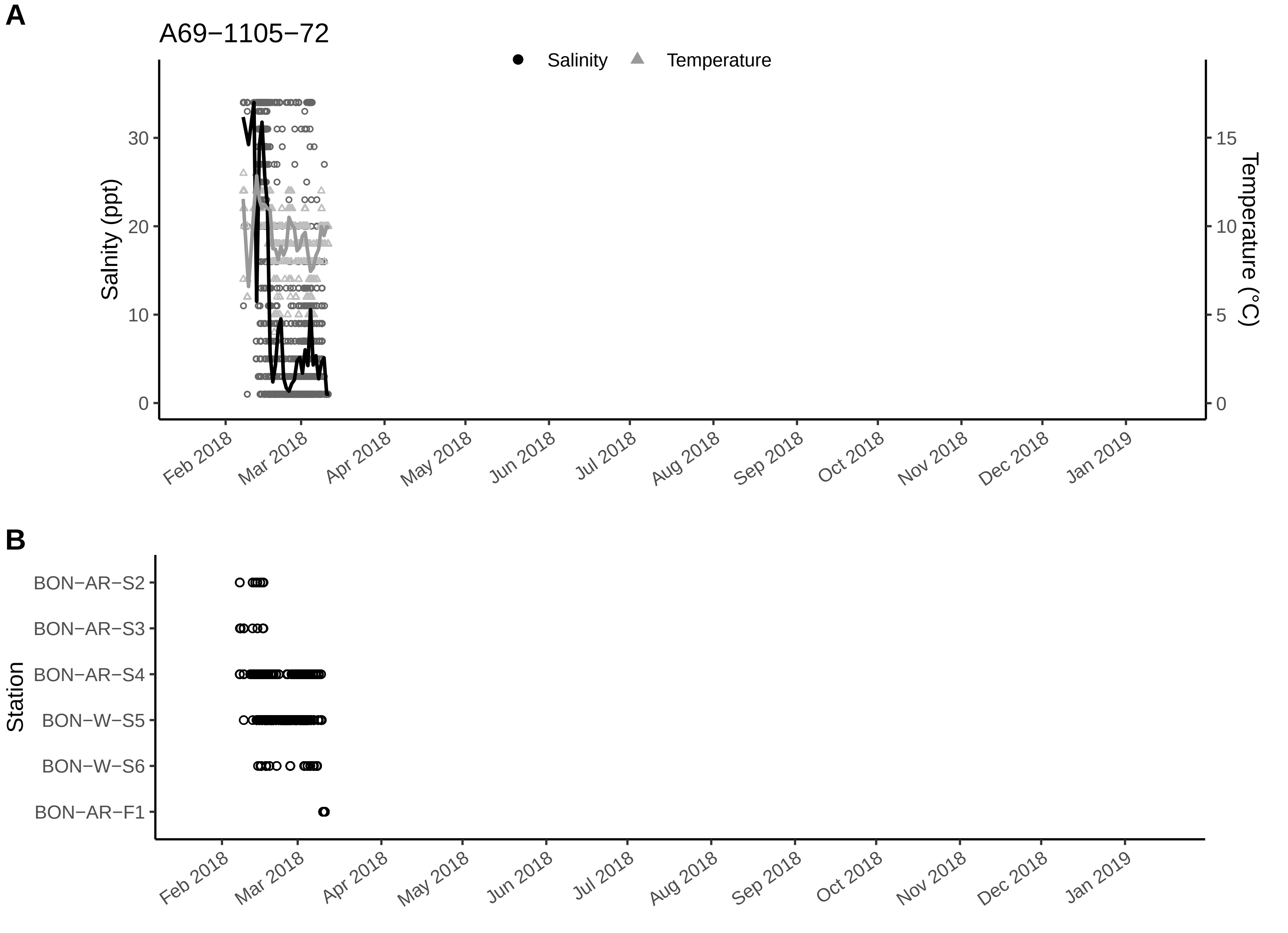
A
A69−1105−72
Salinity
Temperature
Salnity (ppt)
Temperature (°C)
0
10
20
30
0
5
10
15
Feb 2018
Mar 2018
Apr 2018
May 2018
Jun 2018
Jul 2018
Aug 2018
Sep 2018
Oct 2018
Nov 2018
Dec 2018
Jan 2019
B
Station
BON−AR−S2
BON−AR−S3
BON−AR−S4
BON−W−S5
BON−W−S6
BON−AR−F1
Feb 2018
Mar 2018
Apr 2018
May 2018
Jun 2018
Jul 2018
Aug 2018
Sep 2018
Oct 2018
Nov 2018
Dec 2018
Jan 2019

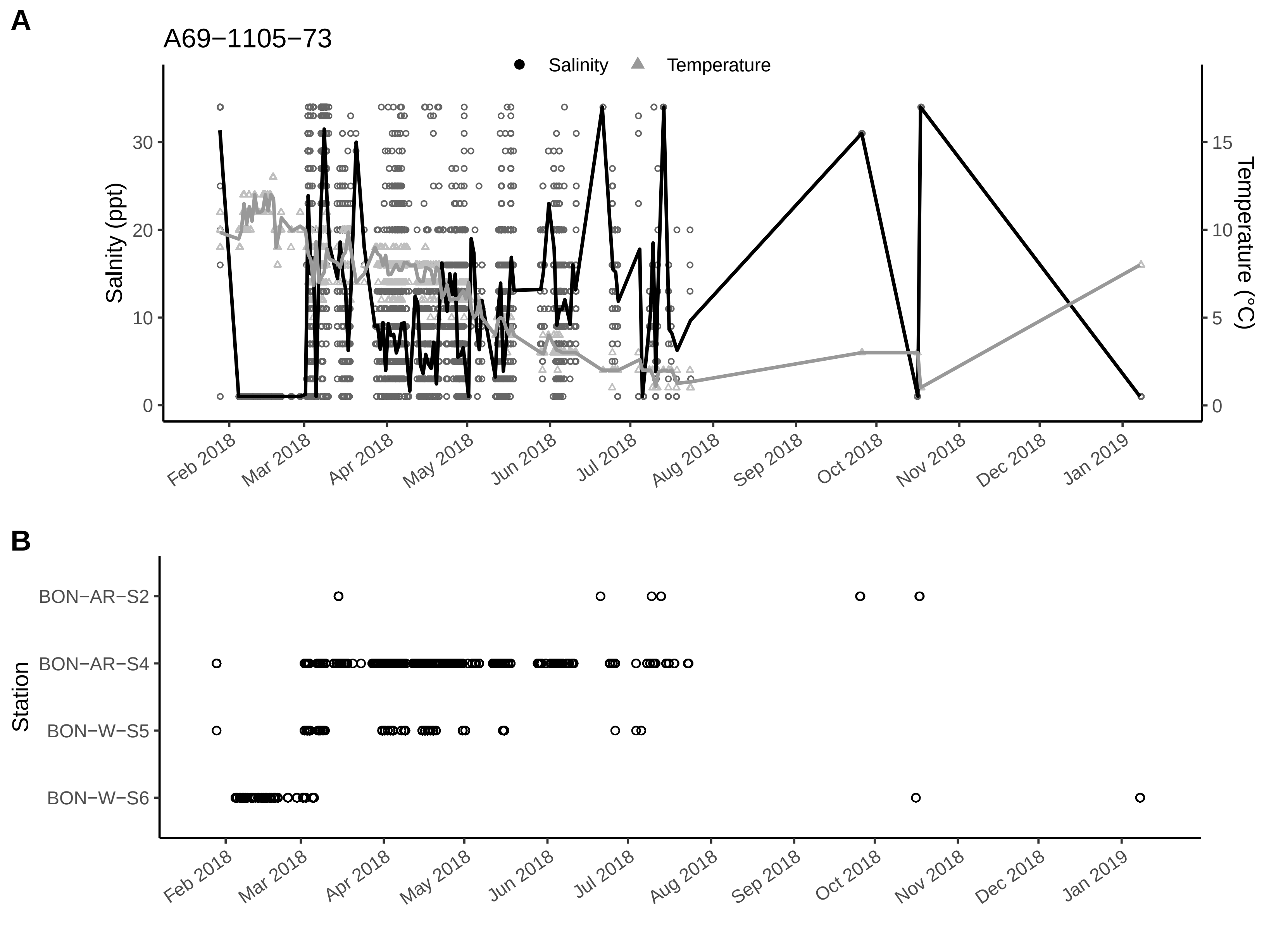

A
A69−1105−73
Salinity
Temperature
Salinity (ppt)
Temperature (°C)
0
10
20
30
0
5
10
15
Feb 2018
Mar 2018
Apr 2018
May 2018
Jun 2018
Jul 2018
Aug 2018
Sep 2018
Oct 2018
Nov 2018
Dec 2018
Jan 2019
B
Station
BON−AR−S2
BON−AR−S4
BON−W−S5
BON−W−S6
Feb 2018
Mar 2018
Apr 2018
May 2018
Jun 2018
Jul 2018
Aug 2018
Sep 2018
Oct 2018
Nov 2018
Dec 2018
Jan 2019

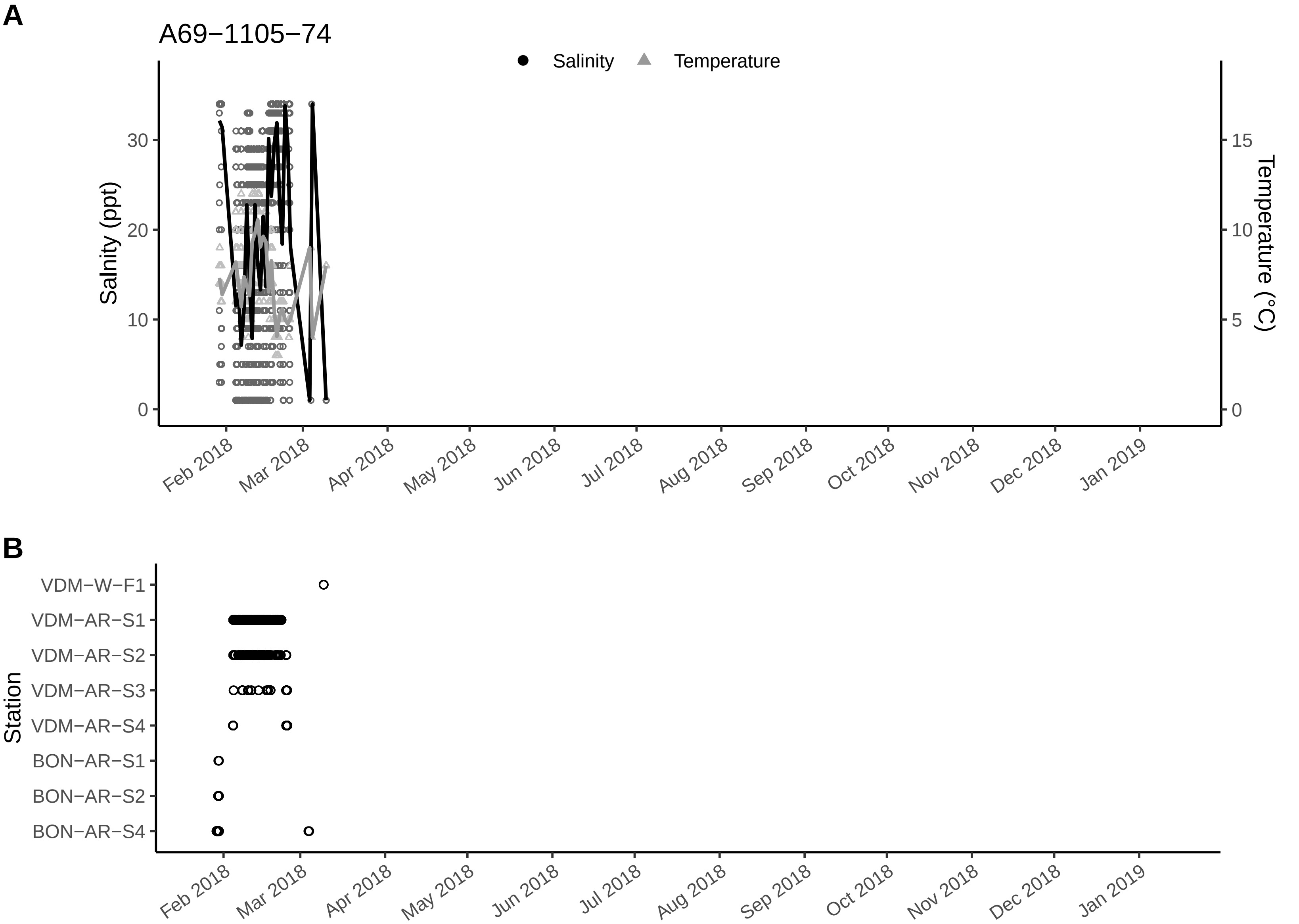

A
A69−1105−74
Salinity
Temperature
Salnity (ppt)
Temperature (°C)
0
10
20
30
0
5
10
15
Feb 2018
Mar 2018
Apr 2018
May 2018
Jun 2018
Jul 2018
Aug 2018
Sep 2018
Oct 2018
Nov 2018
Dec 2018
Jan 2019
B
Station
VDM−W−F1
VDM−AR−S1
VDM−AR−S2
VDM−AR−S3
VDM−AR−S4
BON−AR−S1
BON−AR−S2
BON−AR−S4
Feb 2018
Mar 2018
Apr 2018
May 2018
Jun 2018
Jul 2018
Aug 2018
Sep 2018
Oct 2018
Nov 2018
Dec 2018
Jan 2019

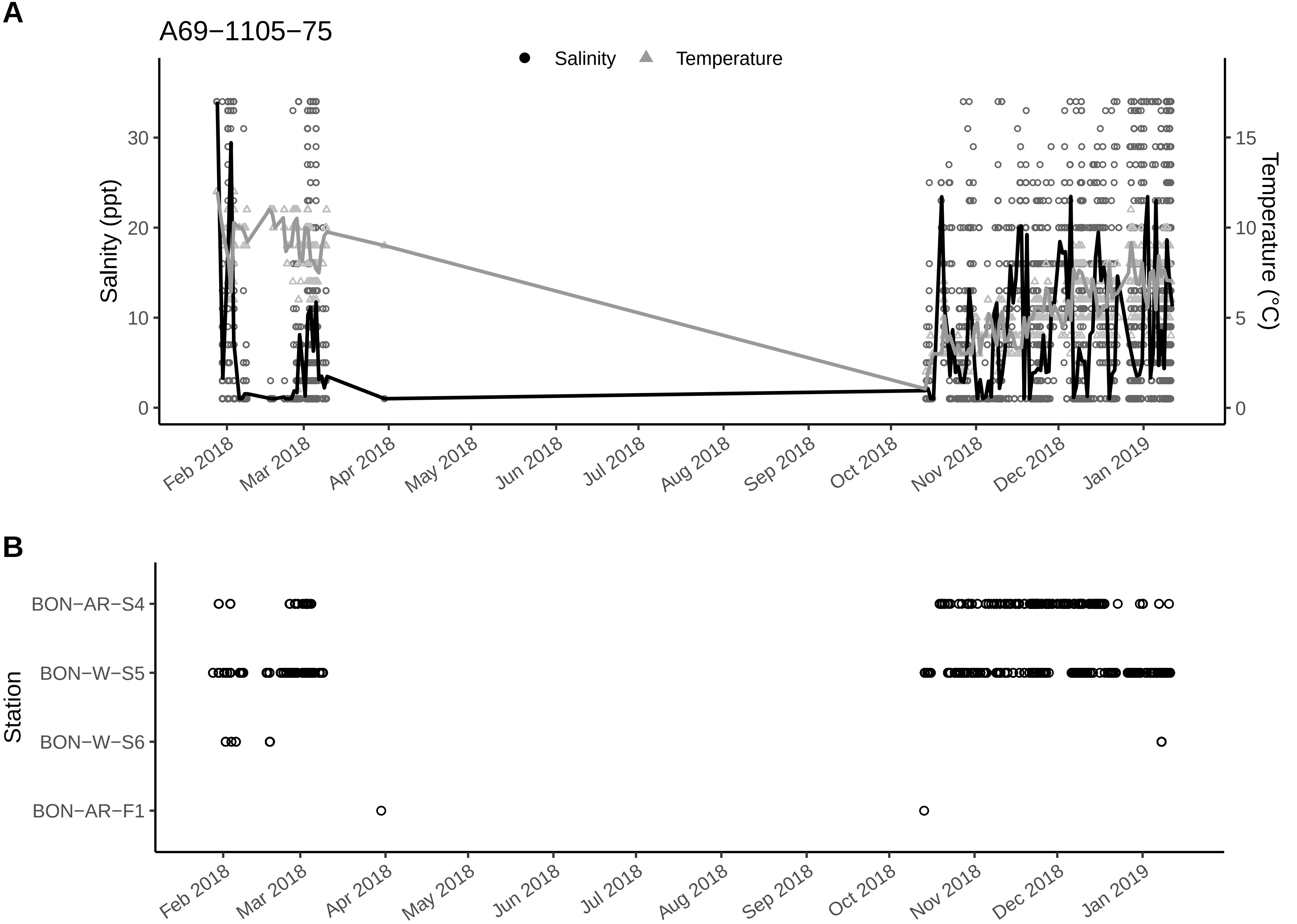

A
A69−1105−75
Salinity
Temperature
Salnity (ppt)
Temperature (°C)
0
10
20
30
0
5
10
15
Feb 2018
Mar 2018
Apr 2018
May 2018
Jun 2018
Jul 2018
Aug 2018
Sep 2018
Oct 2018
Nov 2018
Dec 2018
Jan 2019
B
Station
BON−AR−S4
BON−W−S5
BON−W−S6
BON−AR−F1
Feb 2018
Mar 2018
Apr 2018
May 2018
Jun 2018
Jul 2018
Aug 2018
Sep 2018
Oct 2018
Nov 2018
Dec 2018
Jan 2019

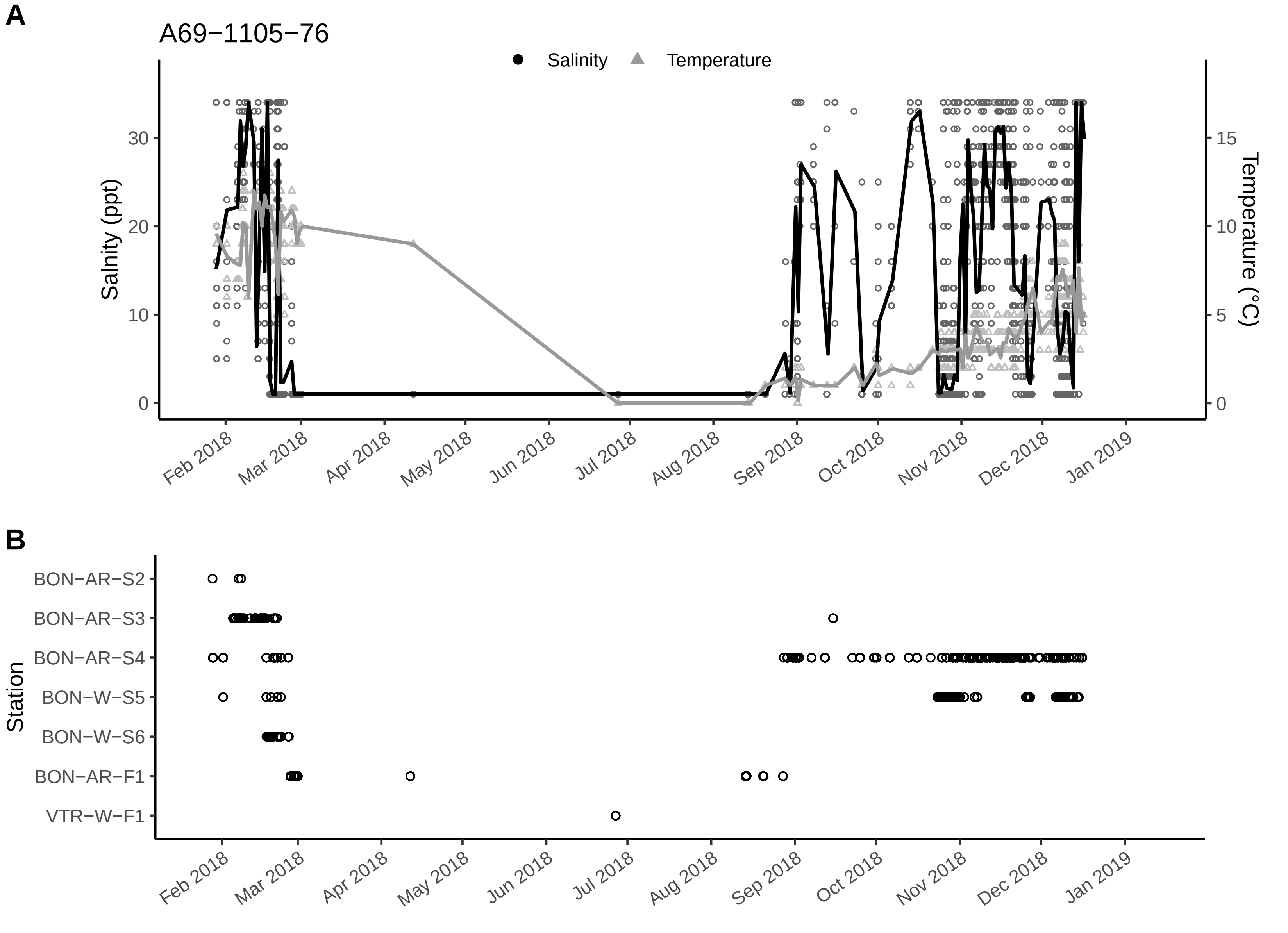

A
A69−1105−76
Salinity
Temperature
Salinity (ppt)
Temperature (°C)
0
10
20
30
0
5
10
15
Feb 2018
Mar 2018
Apr 2018
May 2018
Jun 2018
Jul 2018
Aug 2018
Sep 2018
Oct 2018
Nov 2018
Dec 2018
Jan 2019
B
Station
BON−AR−S2
BON−AR−S3
BON−AR−S4
BON−W−S5
BON−W−S6
BON−AR−F1
VTR−W−F1
Feb 2018
Mar 2018
Apr 2018
May 2018
Jun 2018
Jul 2018
Aug 2018
Sep 2018
Oct 2018
Nov 2018
Dec 2018
Jan 2019

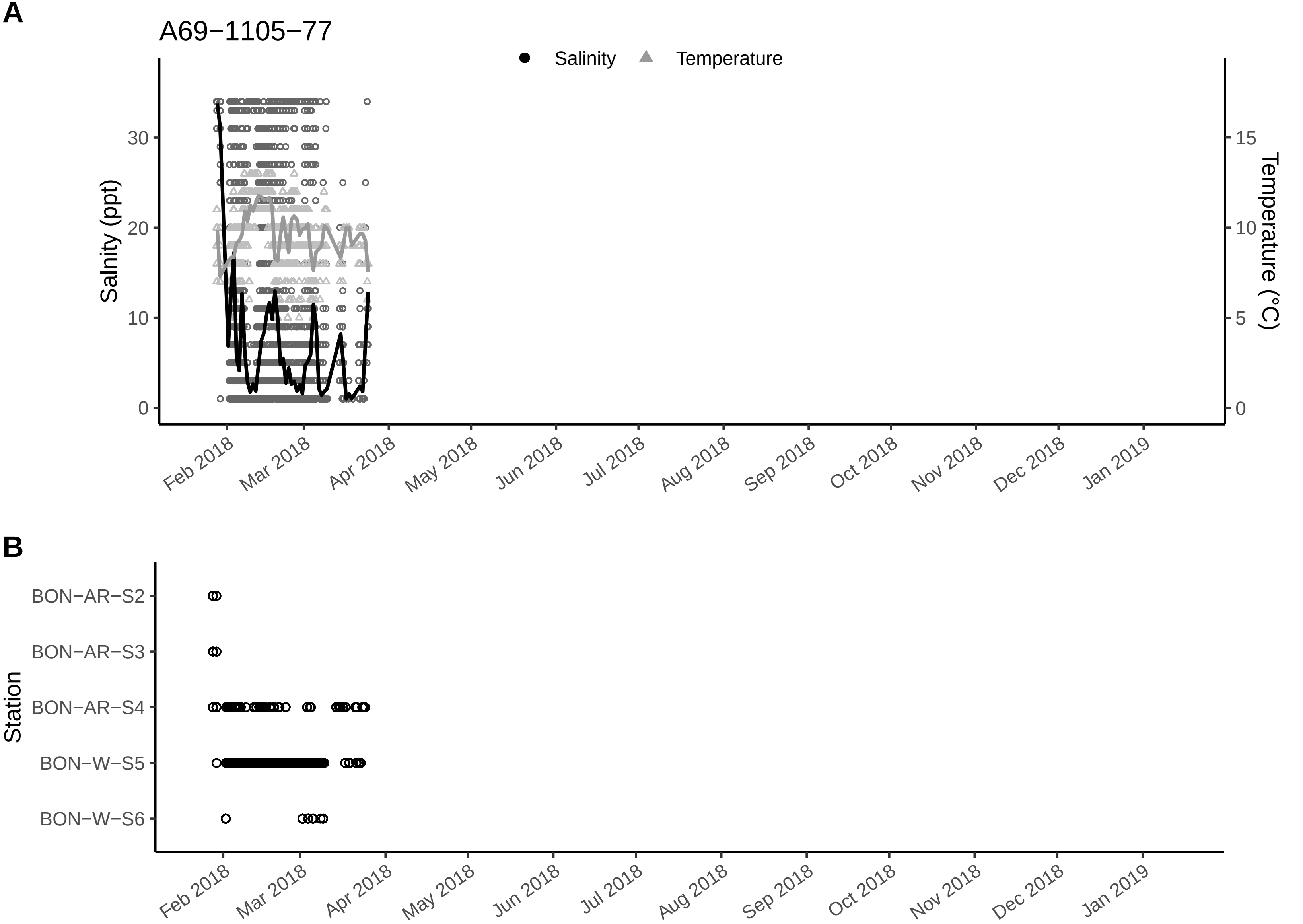
A
A69−1105−77
Salinity
Temperature
Salnity (ppt)
Temperature (°C)
0
10
20
30
0
5
10
15
Feb 2018
Mar 2018
Apr 2018
May 2018
Jun 2018
Jul 2018
Aug 2018
Sep 2018
Oct 2018
Nov 2018
Dec 2018
Jan 2019
B
Station
BON−AR−S2
BON−AR−S3
BON−AR−S4
BON−W−S5
BON−W−S6
Feb 2018
Mar 2018
Apr 2018
May 2018
Jun 2018
Jul 2018
Aug 2018
Sep 2018
Oct 2018
Nov 2018
Dec 2018
Jan 2019

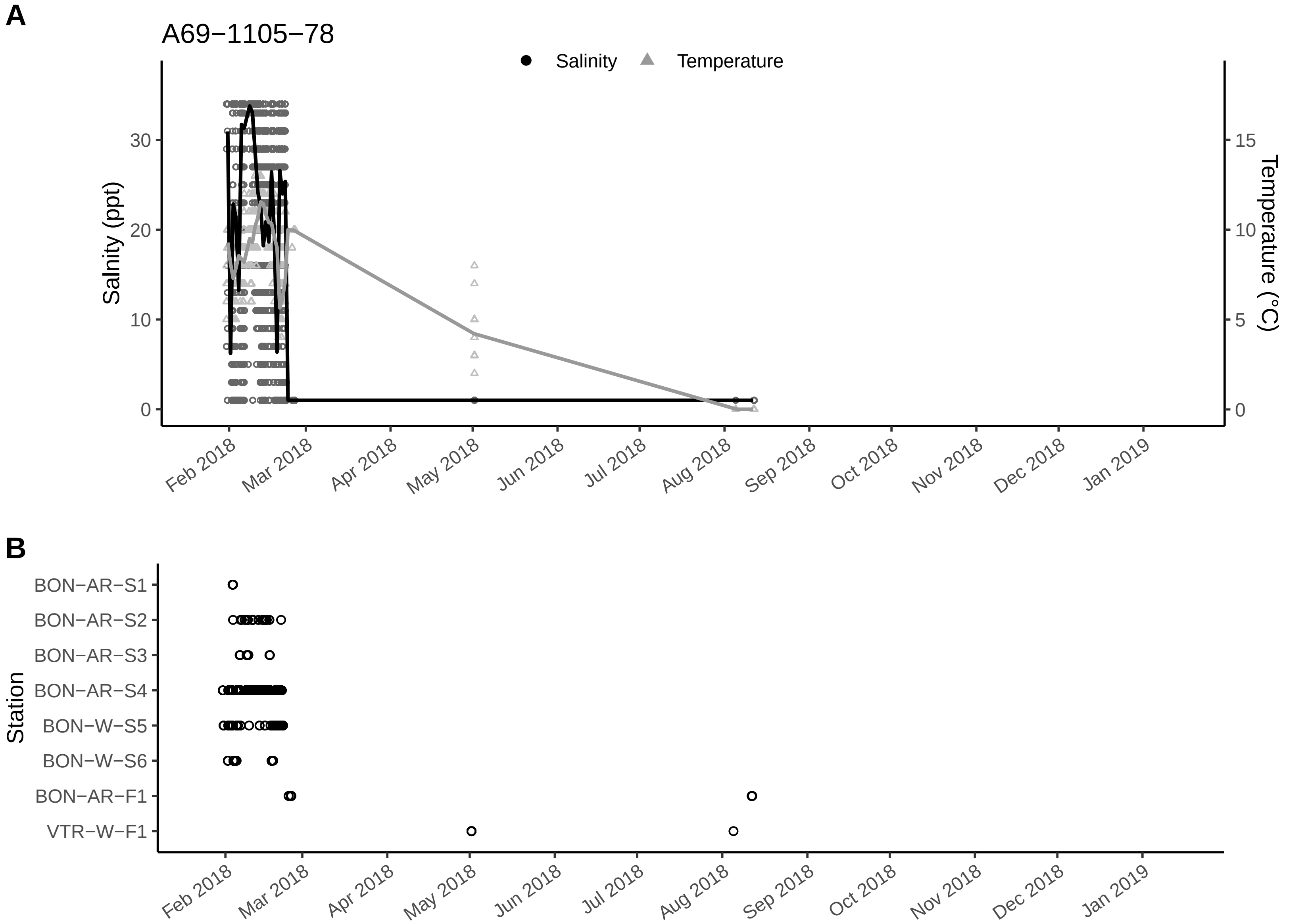
A
A69−1105−78
Salinity
Temperature
Salnity (ppt)
Temperature (°C)
Feb 2018
Mar 2018
Apr 2018
May 2018
Jun 2018
Jul 2018
Aug 2018
Sep 2018
Oct 2018
Nov 2018
Dec 2018
Jan 2019
B
Station
BON−AR−S1
BON−AR−S2
BON−AR−S3
BON−AR−S4
BON−W−S5
BON−W−S6
BON−AR−F1
VTR−W−F1

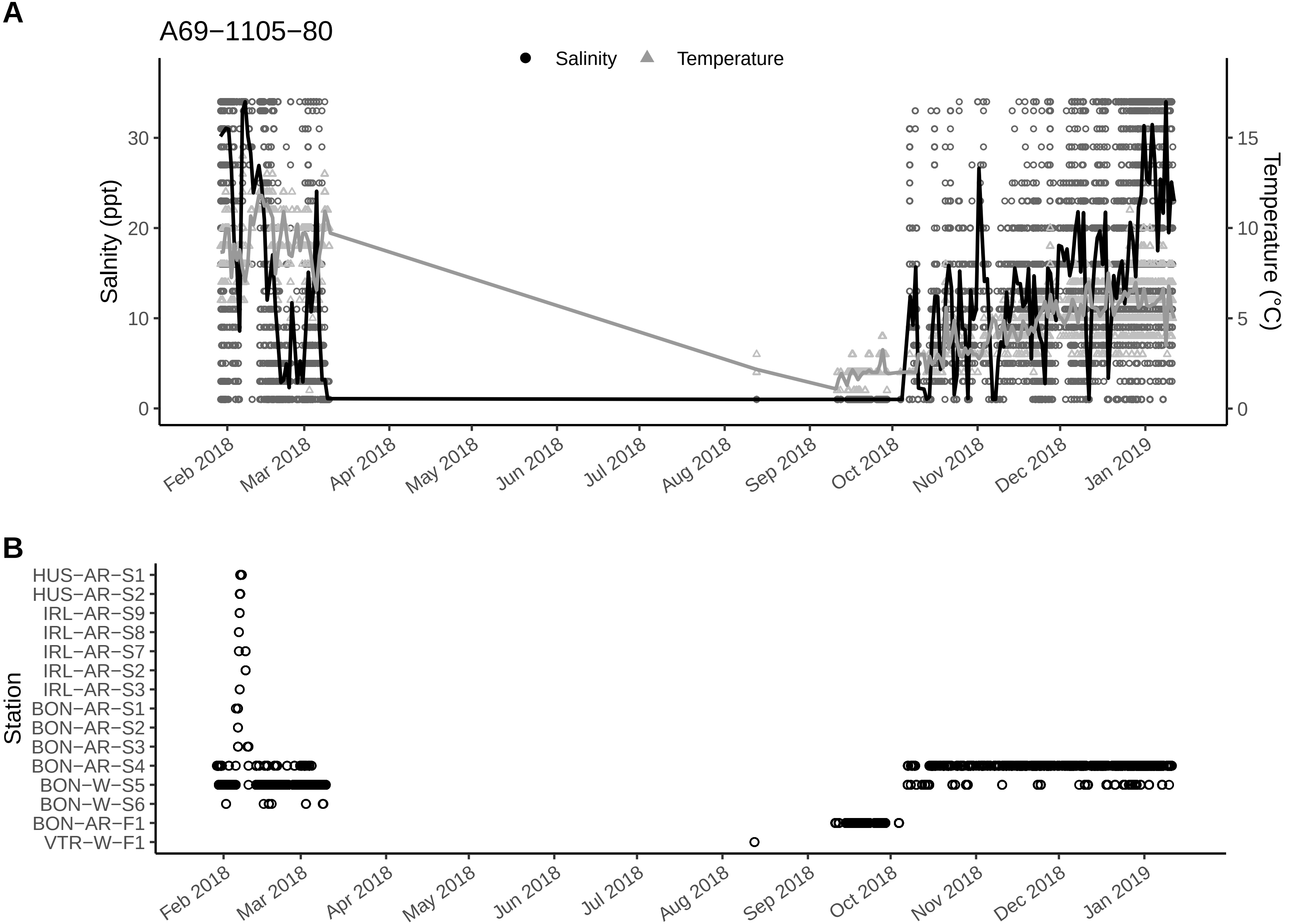
A
A69−1105−80
Salinity
Temperature
Salinity (ppt)
Temperature (°C)
0
10
20
30
0
5
10
15
Feb 2018
Mar 2018
Apr 2018
May 2018
Jun 2018
Jul 2018
Aug 2018
Sep 2018
Oct 2018
Nov 2018
Dec 2018
Jan 2019
B
Station
HUS−AR−S1
HUS−AR−S2
IRL−AR−S9
IRL−AR−S8
IRL−AR−S7
IRL−AR−S2
IRL−AR−S3
BON−AR−S1
BON−AR−S2
BON−AR−S3
BON−AR−S4
BON−W−S5
BON−W−S6
BON−AR−F1
VTR−W−F1
Feb 2018
Mar 2018
Apr 2018
May 2018
Jun 2018
Jul 2018
Aug 2018
Sep 2018
Oct 2018
Nov 2018
Dec 2018
Jan 2019

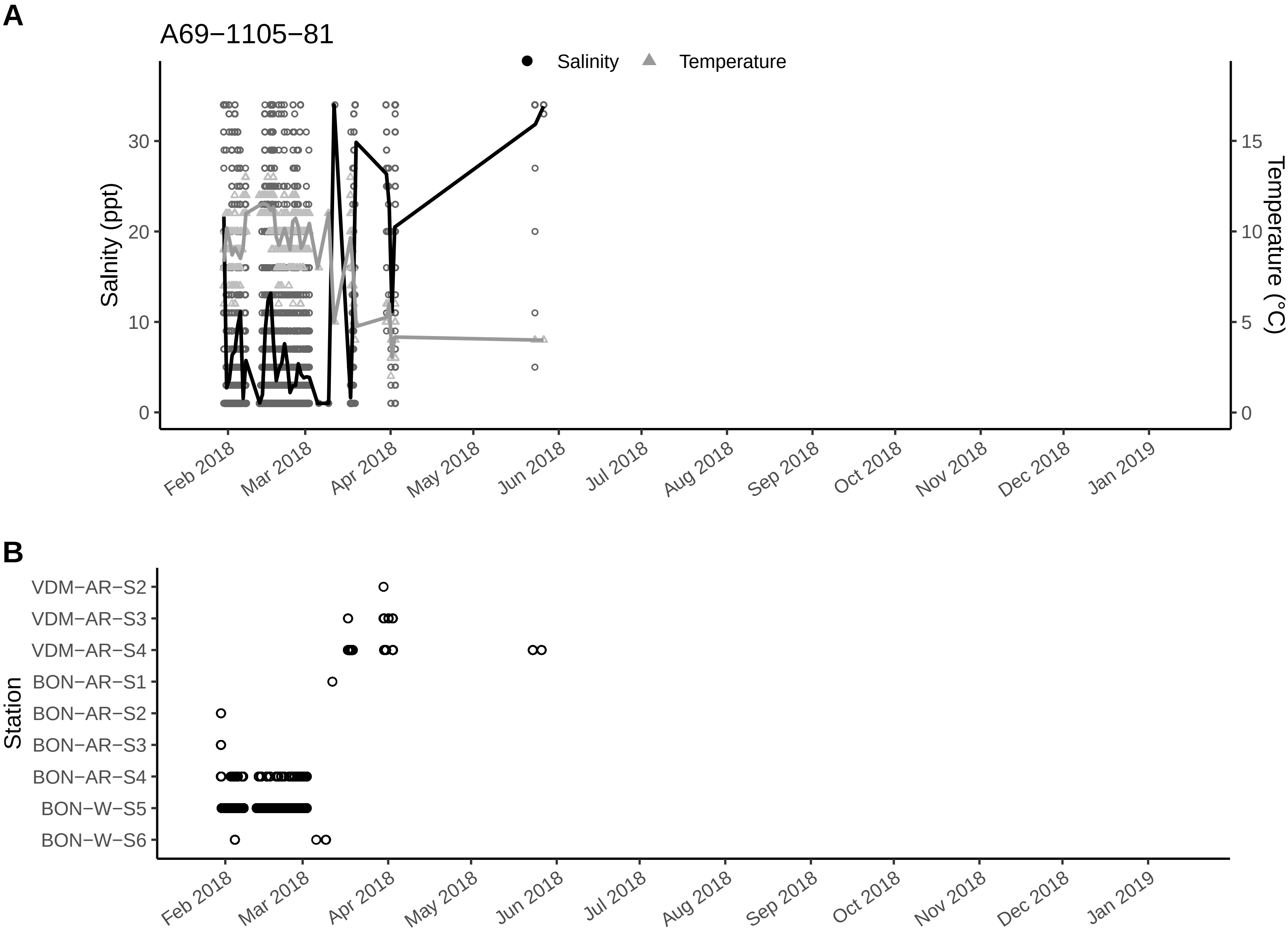
A
A69−1105−81
Salinity
Temperature
Salnity (ppt)
Temperature (°C)
0
10
20
30
0
5
10
15
Feb 2018
Mar 2018
Apr 2018
May 2018
Jun 2018
Jul 2018
Aug 2018
Sep 2018
Oct 2018
Nov 2018
Dec 2018
Jan 2019
B
Station
VDM−AR−S2
VDM−AR−S3
VDM−AR−S4
BON−AR−S1
BON−AR−S2
BON−AR−S3
BON−AR−S4
BON−W−S5
BON−W−S6
Feb 2018
Mar 2018
Apr 2018
May 2018
Jun 2018
Jul 2018
Aug 2018
Sep 2018
Oct 2018
Nov 2018
Dec 2018
Jan 2019

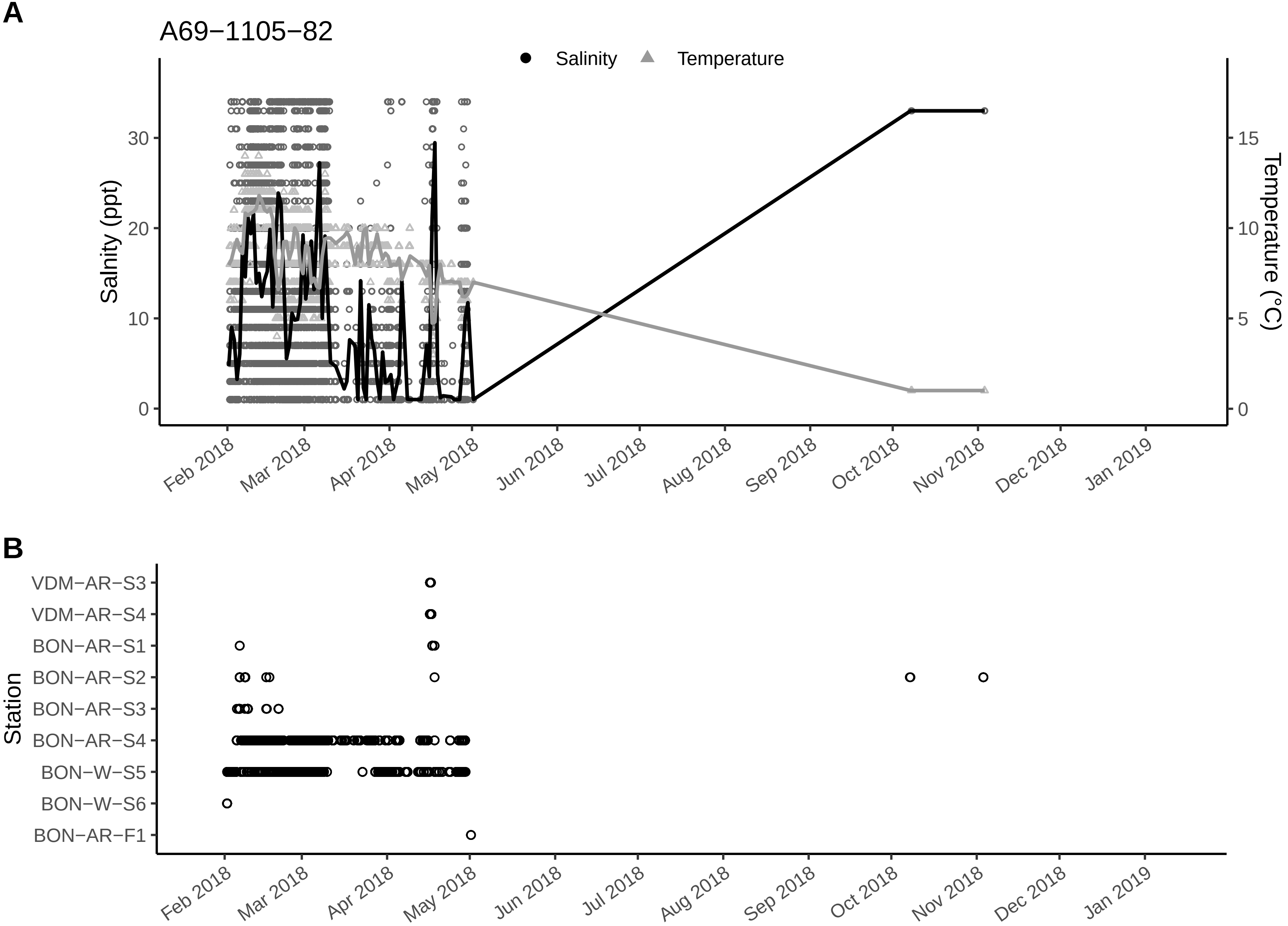
A
A69−1105−82
Salinity
Temperature
Salnity (ppt)
Temperature (°C)
0
10
20
30
0
5
10
15
Feb 2018
Mar 2018
Apr 2018
May 2018
Jun 2018
Jul 2018
Aug 2018
Sep 2018
Oct 2018
Nov 2018
Dec 2018
Jan 2019
B
Station
VDM−AR−S3
VDM−AR−S4
BON−AR−S1
BON−AR−S2
BON−AR−S3
BON−AR−S4
BON−W−S5
BON−W−S6
BON−AR−F1
Feb 2018
Mar 2018
Apr 2018
May 2018
Jun 2018
Jul 2018
Aug 2018
Sep 2018
Oct 2018
Nov 2018
Dec 2018
Jan 2019

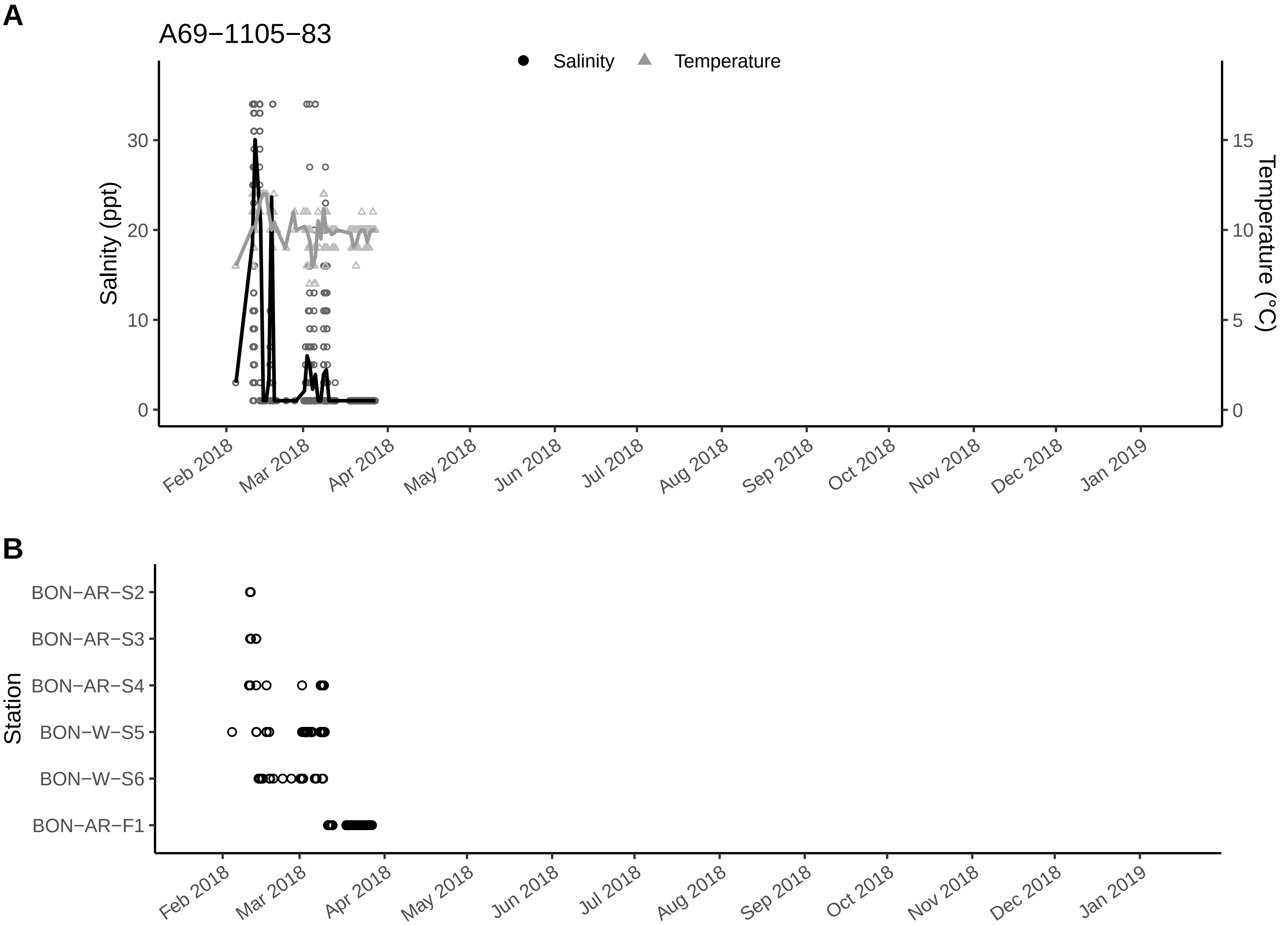
A
A69−1105−83
Salinity
Temperature
Salnity (ppt)
Temperature (°C)
0
10
20
30
0
5
10
15
Feb 2018
Mar 2018
Apr 2018
May 2018
Jun 2018
Jul 2018
Aug 2018
Sep 2018
Oct 2018
Nov 2018
Dec 2018
Jan 2019
B
Station
BON−AR−S2
BON−AR−S3
BON−AR−S4
BON−W−S5
BON−W−S6
BON−AR−F1
Feb 2018
Mar 2018
Apr 2018
May 2018
Jun 2018
Jul 2018
Aug 2018
Sep 2018
Oct 2018
Nov 2018
Dec 2018
Jan 2019

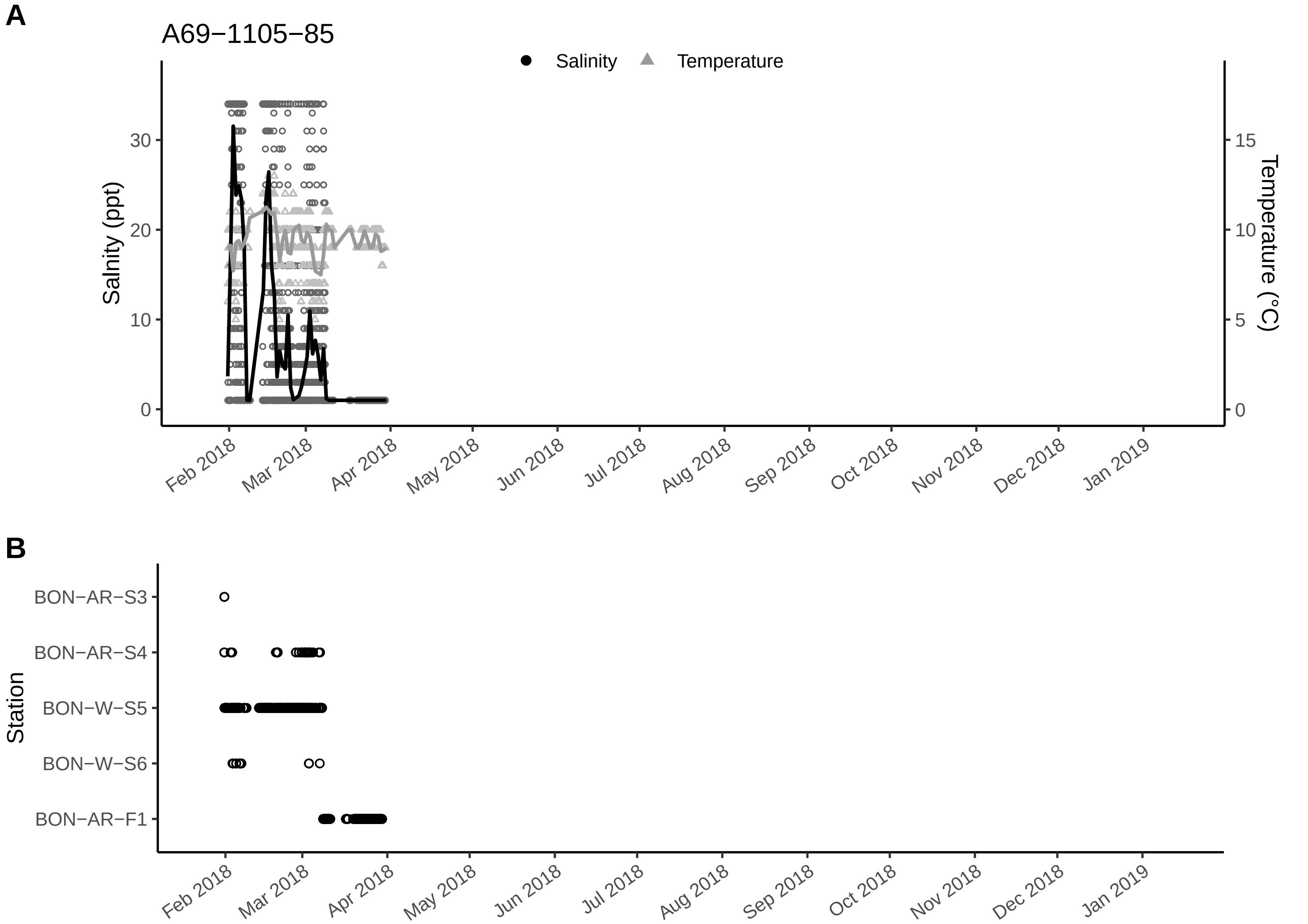
A
A69−1105−85
Salinity
Temperature
Salnity (ppt)
Temperature (°C)
0
10
20
30
0
5
10
15
Feb 2018
Mar 2018
Apr 2018
May 2018
Jun 2018
Jul 2018
Aug 2018
Sep 2018
Oct 2018
Nov 2018
Dec 2018
Jan 2019
B
Station
BON−AR−S3
BON−AR−S4
BON−W−S5
BON−W−S6
BON−AR−F1
Feb 2018
Mar 2018
Apr 2018
May 2018
Jun 2018
Jul 2018
Aug 2018
Sep 2018
Oct 2018
Nov 2018
Dec 2018
Jan 2019

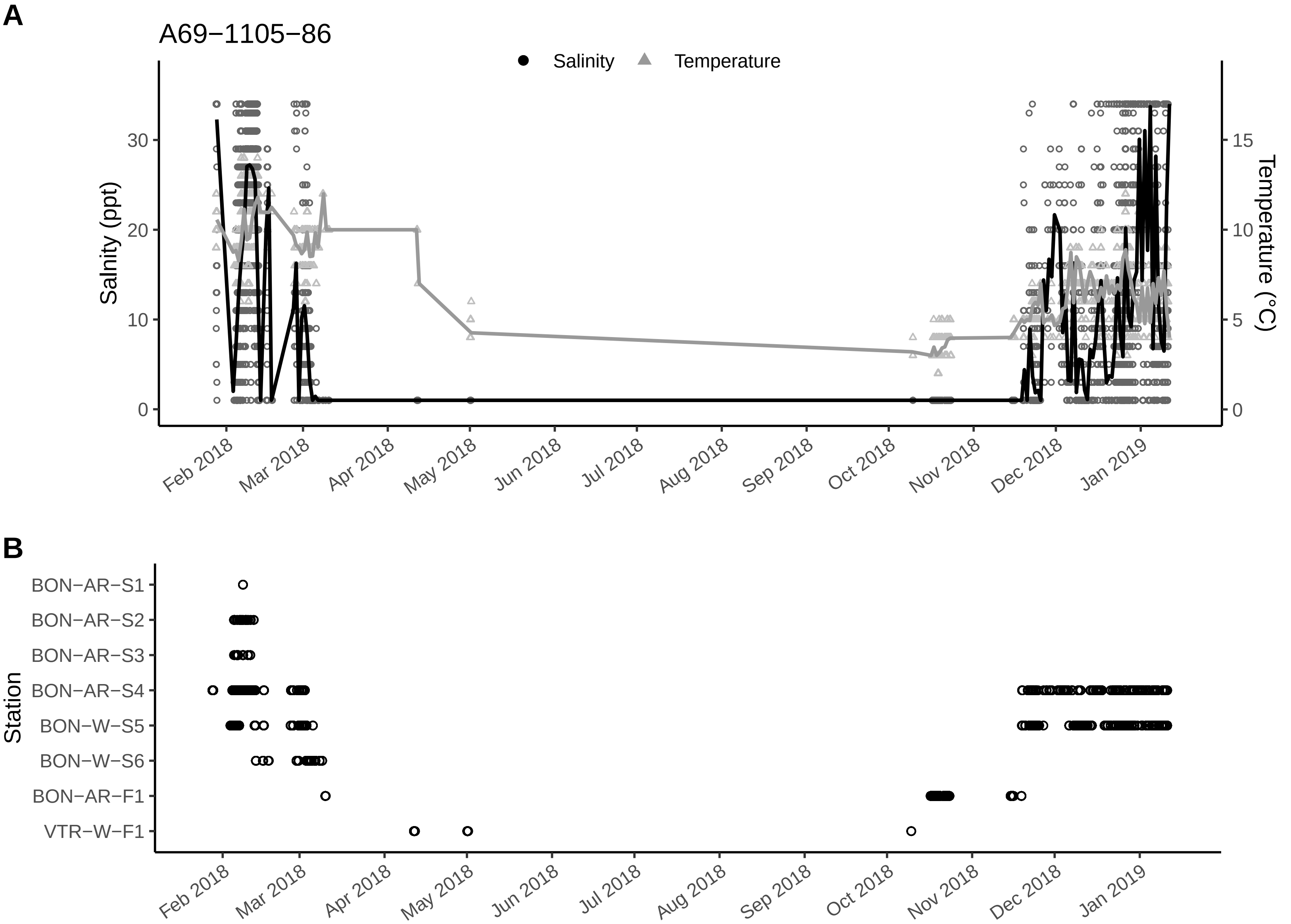
A
A69−1105−86
Salinity
Temperature
Salinity (ppt)
Temperature (°C)
0
10
20
30
0
5
10
15
Feb 2018
Mar 2018
Apr 2018
May 2018
Jun 2018
Jul 2018
Aug 2018
Sep 2018
Oct 2018
Nov 2018
Dec 2018
Jan 2019
B
Station
BON−AR−S1
BON−AR−S2
BON−AR−S3
BON−AR−S4
BON−W−S5
BON−W−S6
BON−AR−F1
VTR−W−F1
Feb 2018
Mar 2018
Apr 2018
May 2018
Jun 2018
Jul 2018
Aug 2018
Sep 2018
Oct 2018
Nov 2018
Dec 2018
Jan 2019

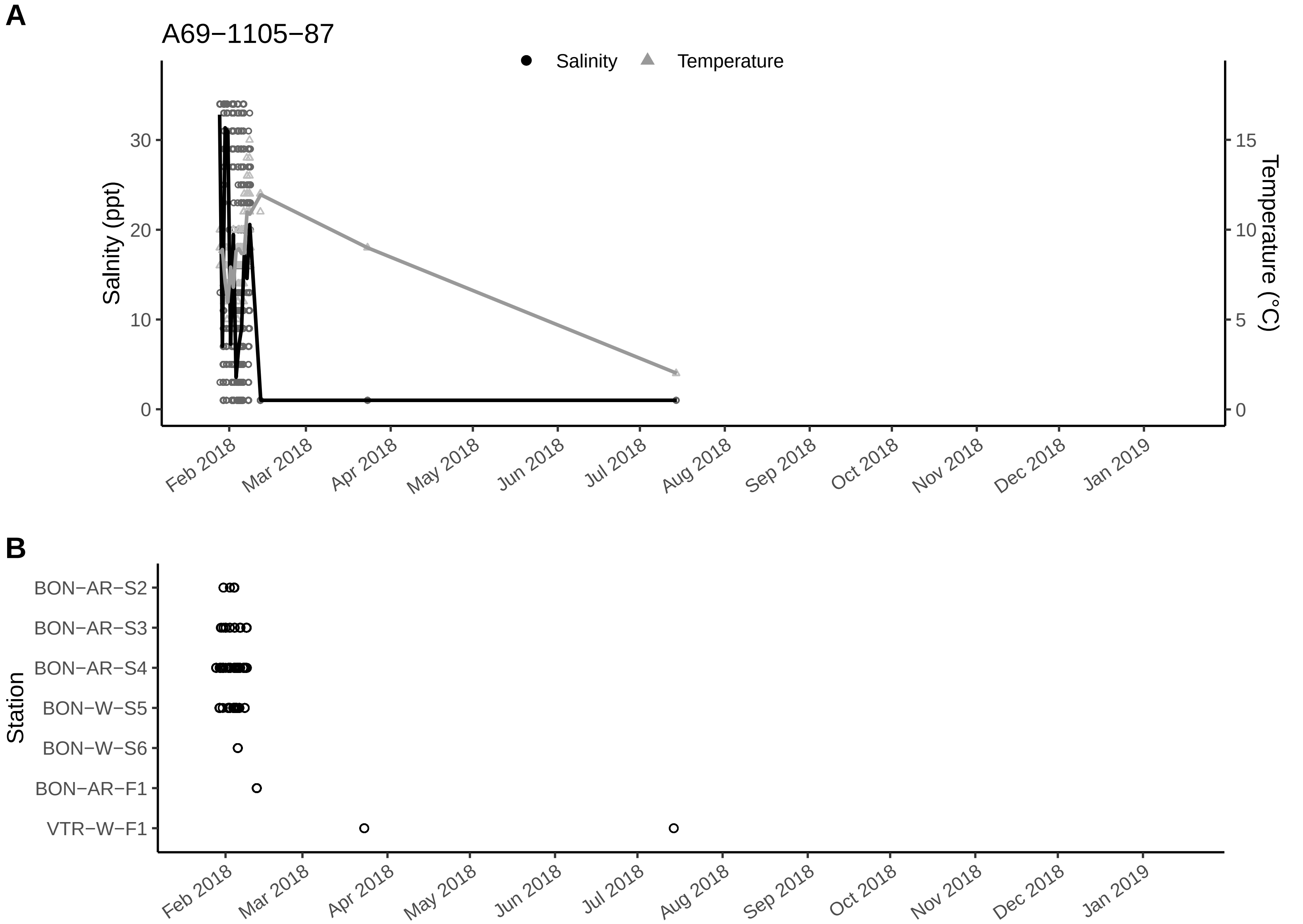

A
A69−1105−87
Salinity
Temperature
Salnity (ppt)
Temperature (°C)
0
10
20
30
0
5
10
15
Feb 2018
Mar 2018
Apr 2018
May 2018
Jun 2018
Jul 2018
Aug 2018
Sep 2018
Oct 2018
Nov 2018
Dec 2018
Jan 2019
B
Station
BON−AR−S2
BON−AR−S3
BON−AR−S4
BON−W−S5
BON−W−S6
BON−AR−F1
VTR−W−F1
Feb 2018
Mar 2018
Apr 2018
May 2018
Jun 2018
Jul 2018
Aug 2018
Sep 2018
Oct 2018
Nov 2018
Dec 2018
Jan 2019

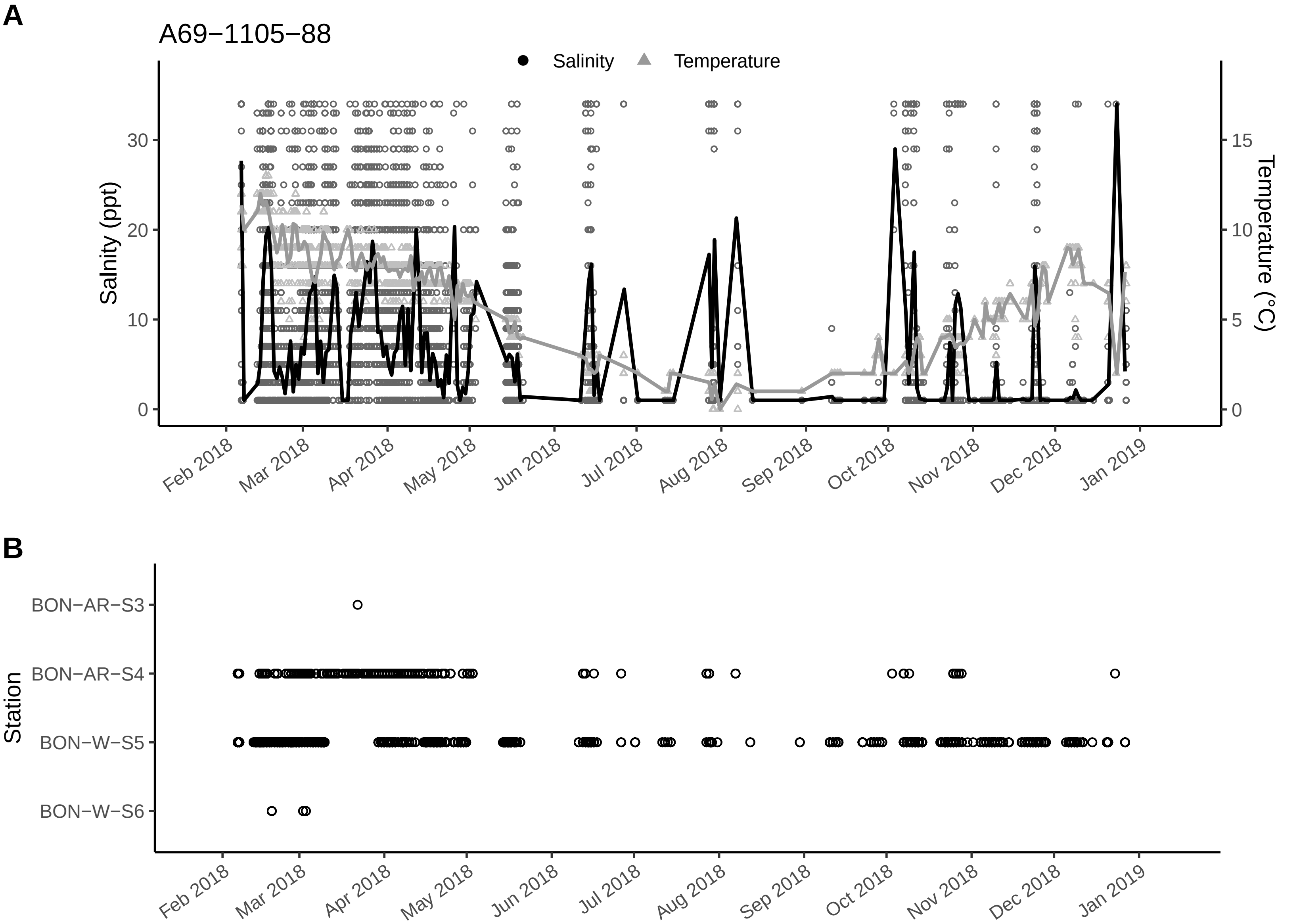
A
A69−1105−88
Salinity
Temperature
Salinity (ppt)
Temperature (°C)
0
10
20
30
0
5
10
15
Feb 2018
Mar 2018
Apr 2018
May 2018
Jun 2018
Jul 2018
Aug 2018
Sep 2018
Oct 2018
Nov 2018
Dec 2018
Jan 2019
B
Station
BON−AR−S3
BON−AR−S4
BON−W−S5
BON−W−S6
Feb 2018
Mar 2018
Apr 2018
May 2018
Jun 2018
Jul 2018
Aug 2018
Sep 2018
Oct 2018
Nov 2018
Dec 2018
Jan 2019

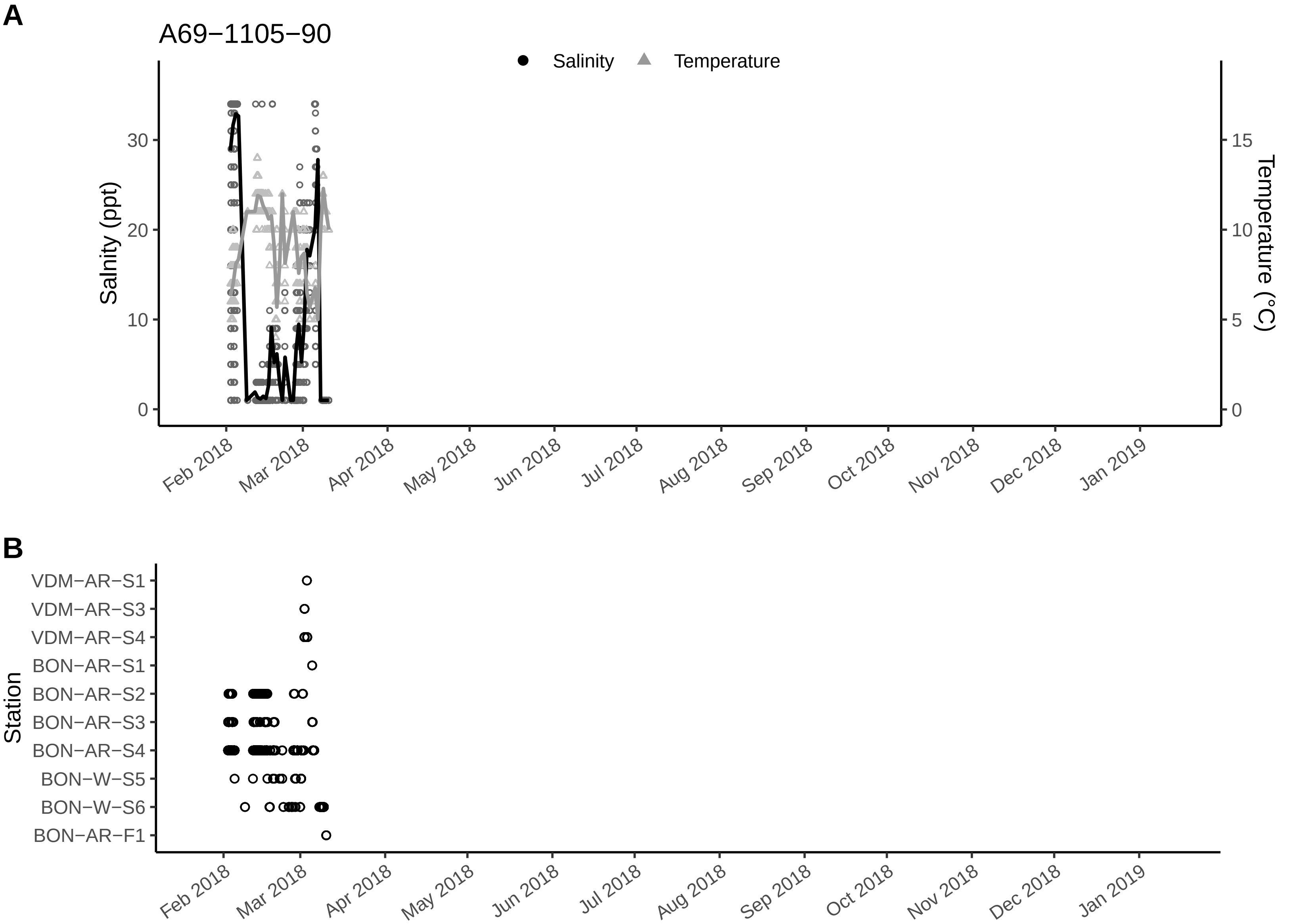

A
A69−1105−90
Salinity
Temperature
Salinity (ppt)
Temperature (°C)
0
10
20
30
0
5
10
15
Feb 2018
Mar 2018
Apr 2018
May 2018
Jun 2018
Jul 2018
Aug 2018
Sep 2018
Oct 2018
Nov 2018
Dec 2018
Jan 2019
B
Station
VDM−AR−S1
VDM−AR−S3
VDM−AR−S4
BON−AR−S1
BON−AR−S2
BON−AR−S3
BON−AR−S4
BON−W−S5
BON−W−S6
BON−AR−F1
Feb 2018
Mar 2018
Apr 2018
May 2018
Jun 2018
Jul 2018
Aug 2018
Sep 2018
Oct 2018
Nov 2018
Dec 2018
Jan 2019

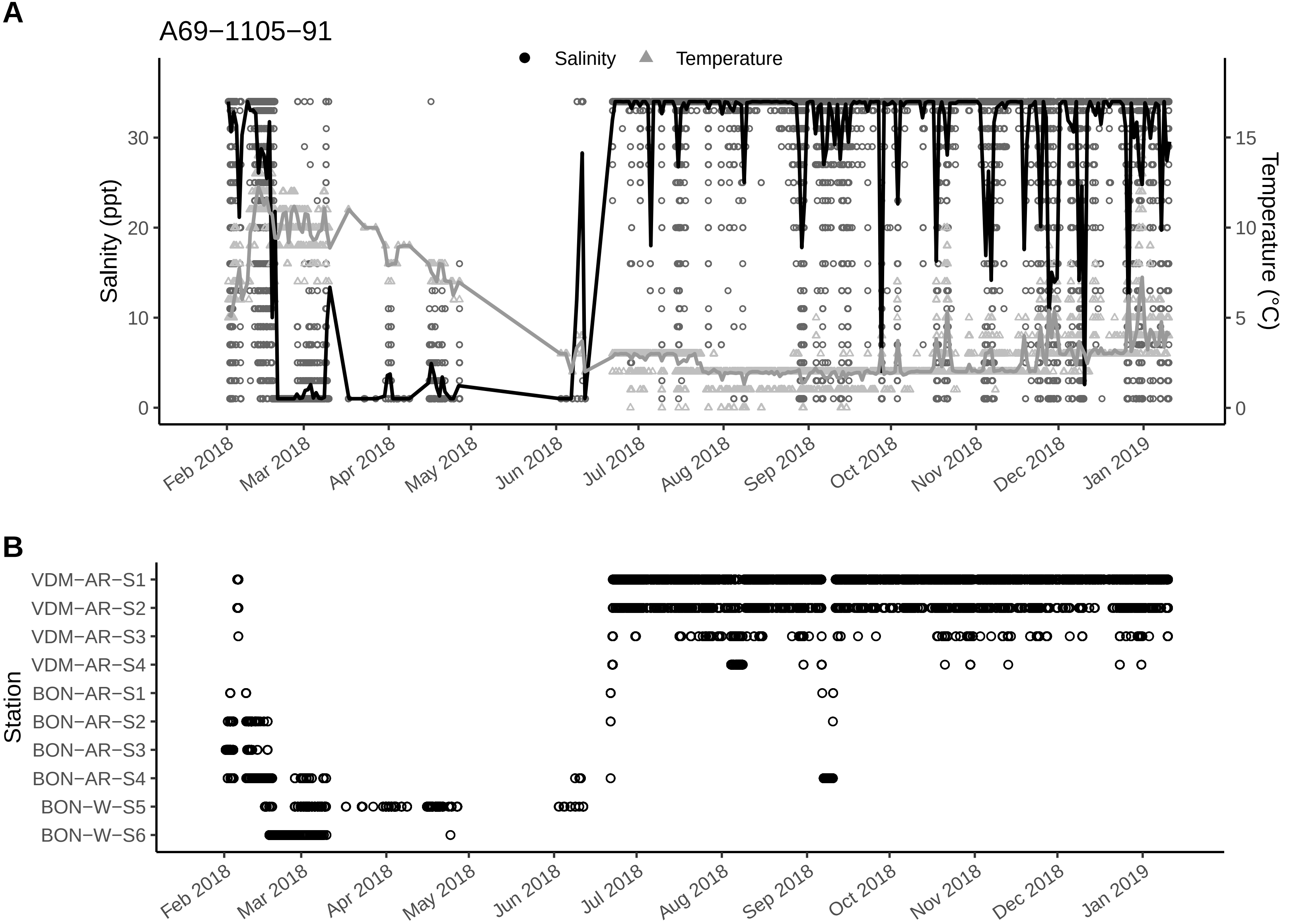
A
A69−1105−91
Salinity
Temperature
Salinity (ppt)
Temperature (°C)
0
10
20
30
0
5
10
15
Feb 2018
Mar 2018
Apr 2018
May 2018
Jun 2018
Jul 2018
Aug 2018
Sep 2018
Oct 2018
Nov 2018
Dec 2018
Jan 2019
B
Station
VDM−AR−S1
VDM−AR−S2
VDM−AR−S3
VDM−AR−S4
BON−AR−S1
BON−AR−S2
BON−AR−S3
BON−AR−S4
BON−W−S5
BON−W−S6
Feb 2018
Mar 2018
Apr 2018
May 2018
Jun 2018
Jul 2018
Aug 2018
Sep 2018
Oct 2018
Nov 2018
Dec 2018
Jan 2019

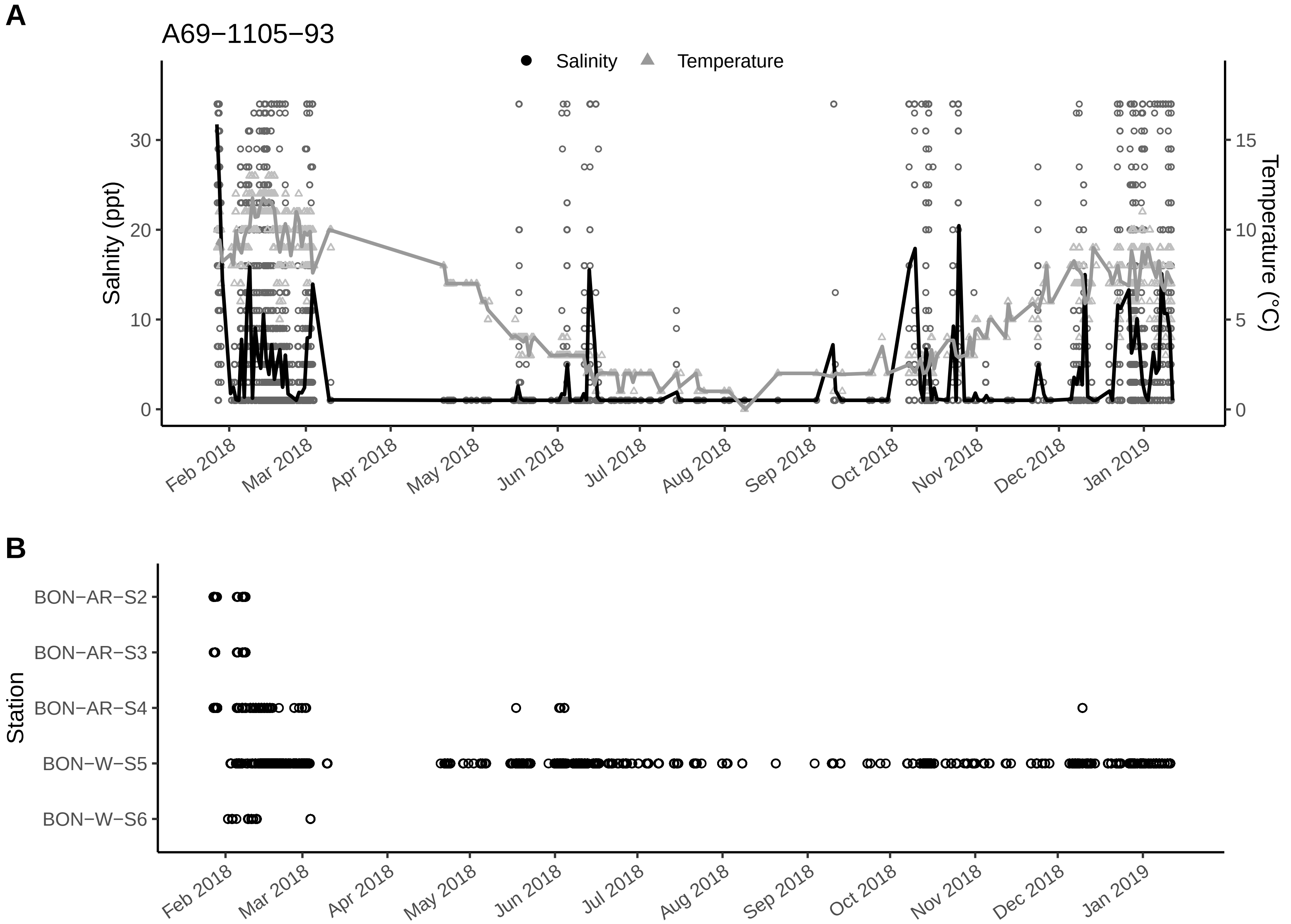
A
A69−1105−93
Salinity
Temperature
Salinity (ppt)
Temperature (°C)
0
10
20
30
0
5
10
15
Feb 2018
Mar 2018
Apr 2018
May 2018
Jun 2018
Jul 2018
Aug 2018
Sep 2018
Oct 2018
Nov 2018
Dec 2018
Jan 2019
B
Station
BON−AR−S2
BON−AR−S3
BON−AR−S4
BON−W−S5
BON−W−S6
Feb 2018
Mar 2018
Apr 2018
May 2018
Jun 2018
Jul 2018
Aug 2018
Sep 2018
Oct 2018
Nov 2018
Dec 2018
Jan 2019

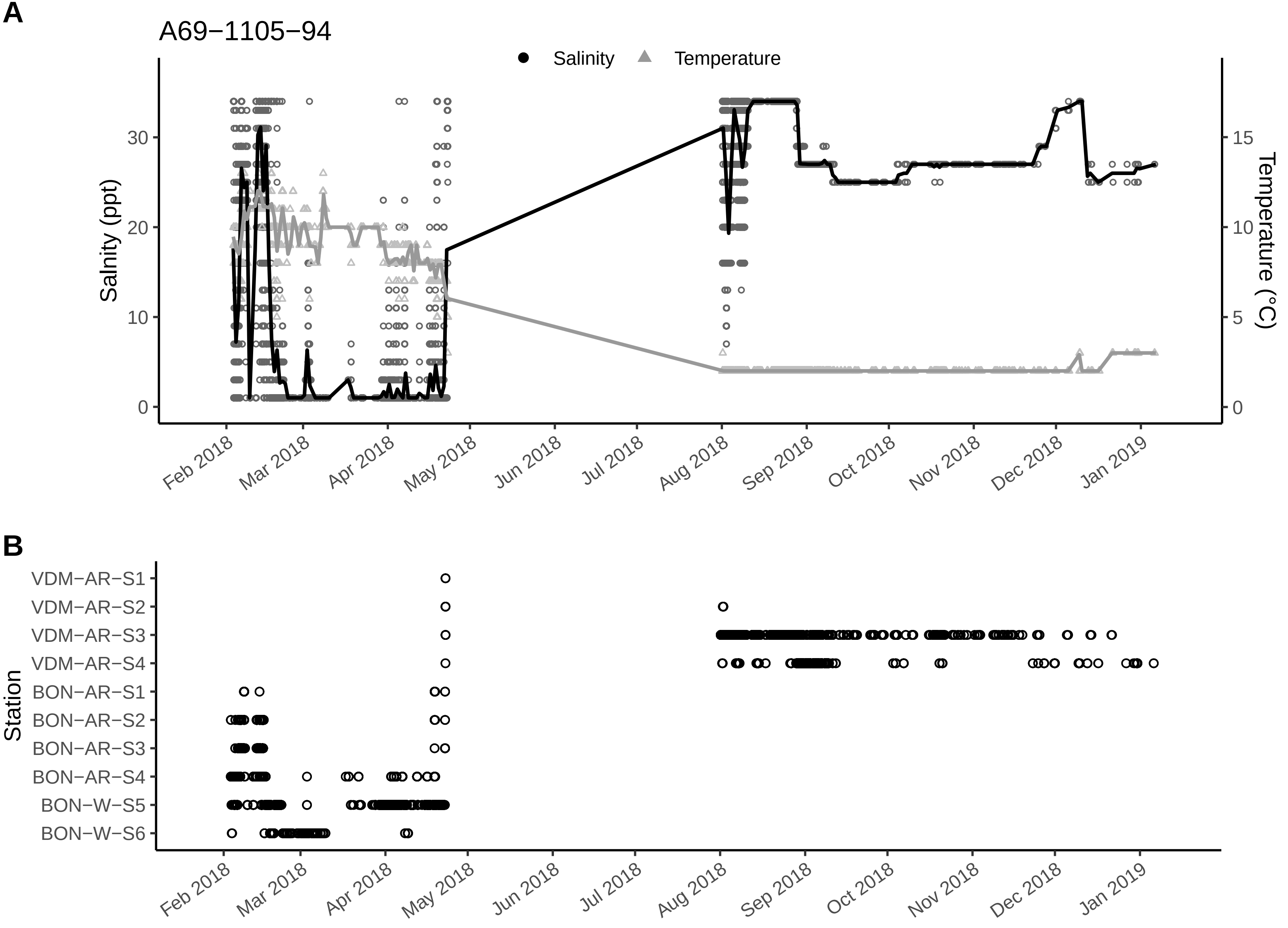
A
A69−1105−94
Salinity
Temperature
Salinity (ppt)
Temperature (°C)
0
10
20
30
0
5
10
15
Feb 2018
Mar 2018
Apr 2018
May 2018
Jun 2018
Jul 2018
Aug 2018
Sep 2018
Oct 2018
Nov 2018
Dec 2018
Jan 2019
B
Station
VDM−AR−S1
VDM−AR−S2
VDM−AR−S3
VDM−AR−S4
BON−AR−S1
BON−AR−S2
BON−AR−S3
BON−AR−S4
BON−W−S5
BON−W−S6
Feb 2018
Mar 2018
Apr 2018
May 2018
Jun 2018
Jul 2018
Aug 2018
Sep 2018
Oct 2018
Nov 2018
Dec 2018
Jan 2019

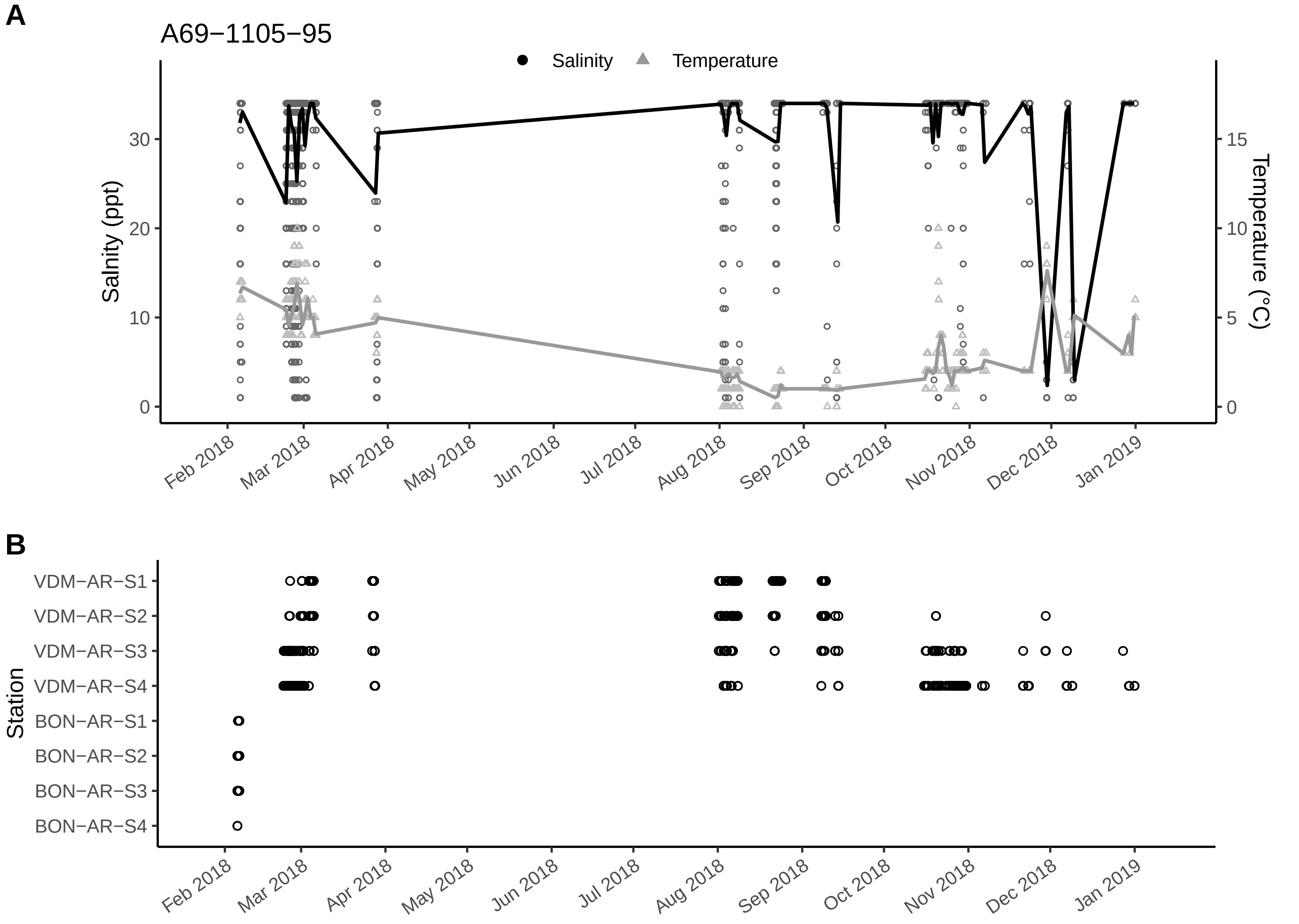
A
A69−1105−95
Salinity
Temperature
Salnity (ppt)
Temperature (°C)
0
10
20
30
0
5
10
15
Feb 2018
Mar 2018
Apr 2018
May 2018
Jun 2018
Jul 2018
Aug 2018
Sep 2018
Oct 2018
Nov 2018
Dec 2018
Jan 2019
B
Station
VDM−AR−S1
VDM−AR−S2
VDM−AR−S3
VDM−AR−S4
BON−AR−S1
BON−AR−S2
BON−AR−S3
BON−AR−S4
Feb 2018
Mar 2018
Apr 2018
May 2018
Jun 2018
Jul 2018
Aug 2018
Sep 2018
Oct 2018
Nov 2018
Dec 2018
Jan 2019

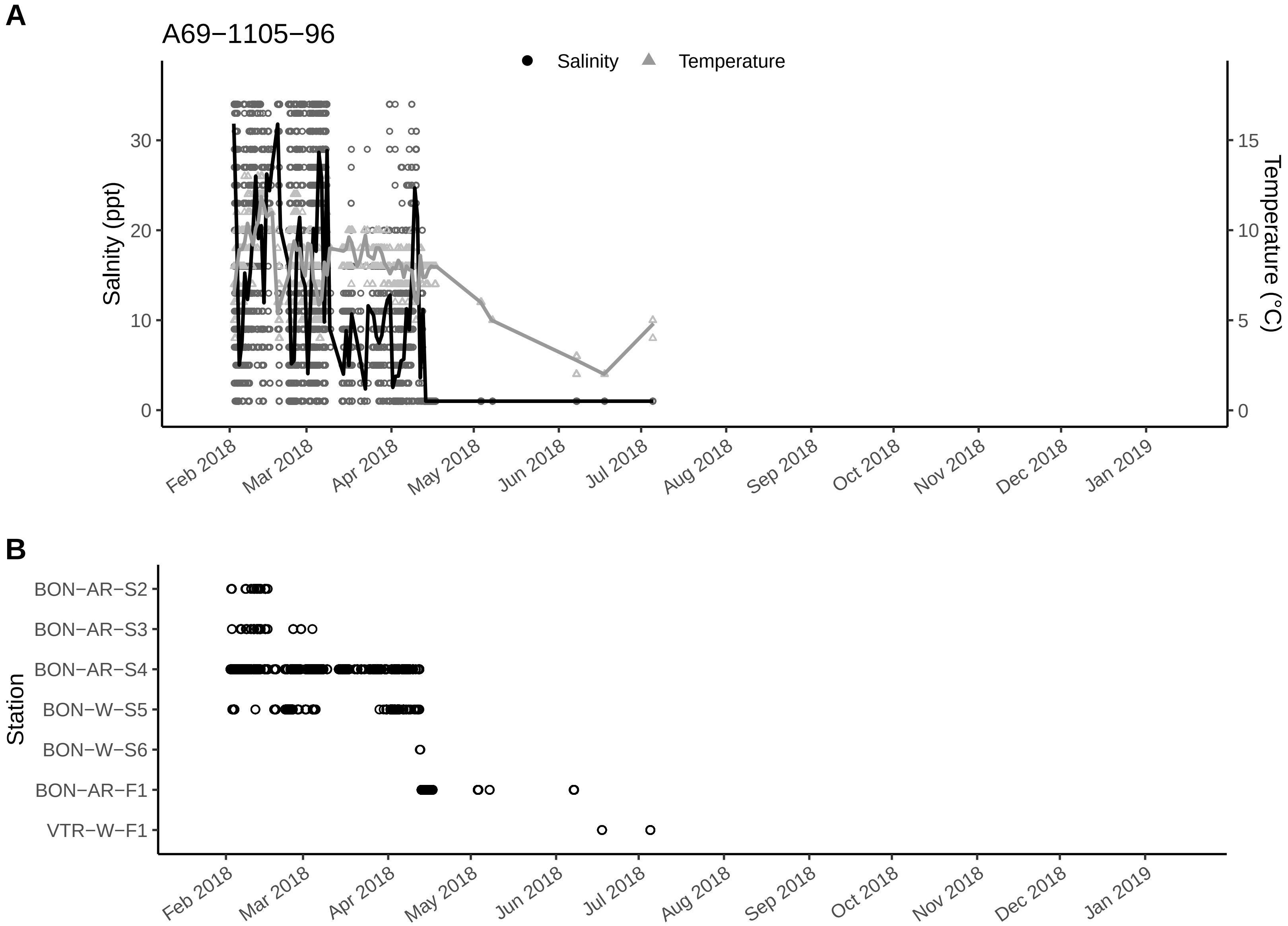

A
A69−1105−96
Salinity
Temperature
Salnity (ppt)
Temperature (°C)
0
10
20
30
0
5
10
15
Feb 2018
Mar 2018
Apr 2018
May 2018
Jun 2018
Jul 2018
Aug 2018
Sep 2018
Oct 2018
Nov 2018
Dec 2018
Jan 2019
B
Station
BON−AR−S2
BON−AR−S3
BON−AR−S4
BON−W−S5
BON−W−S6
BON−AR−F1
VTR−W−F1

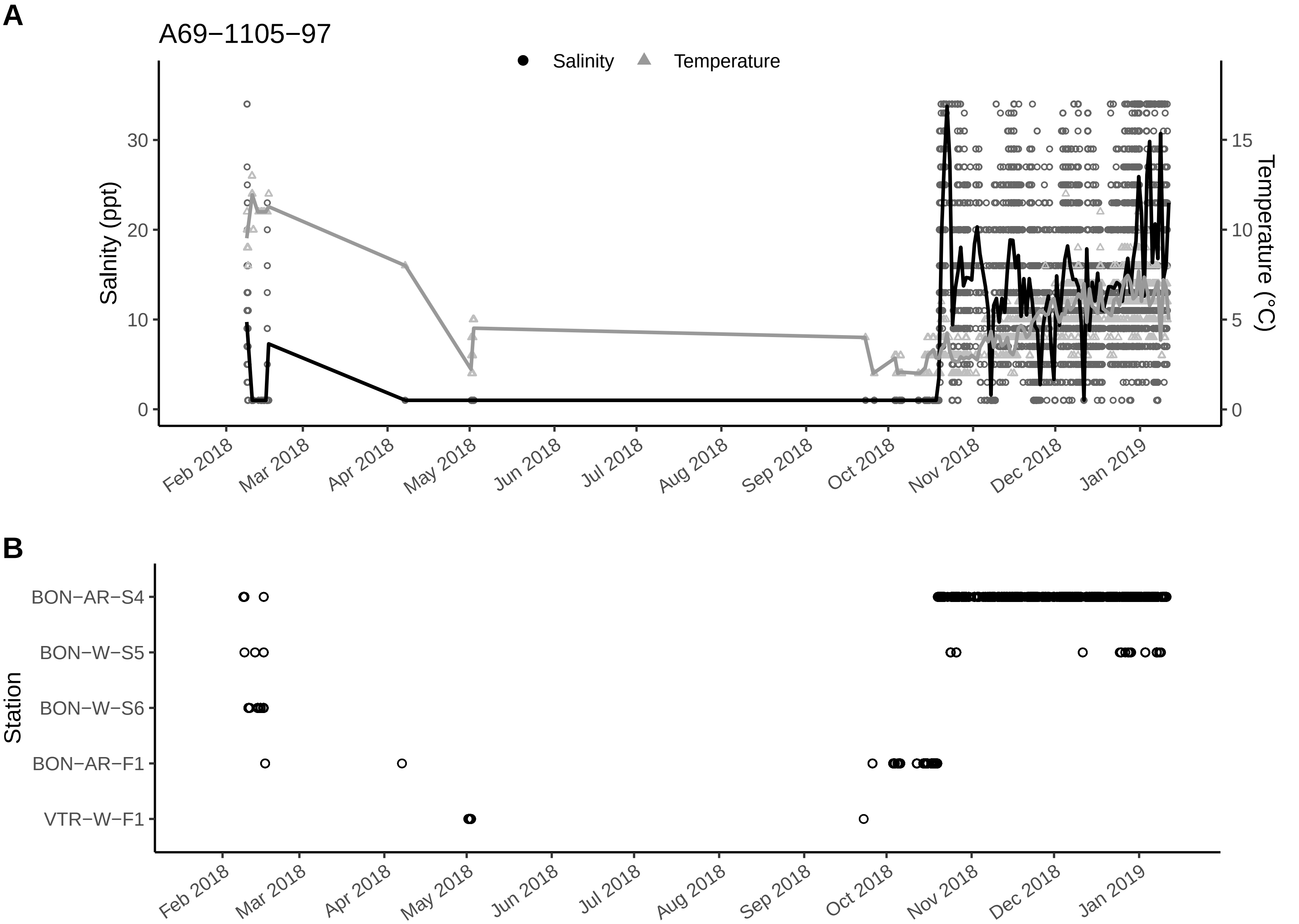

A
A69−1105−97
Salinity
Temperature
Salinity (ppt)
Temperature (°C)
0
10
20
30
0
5
10
15
Feb 2018
Mar 2018
Apr 2018
May 2018
Jun 2018
Jul 2018
Aug 2018
Sep 2018
Oct 2018
Nov 2018
Dec 2018
Jan 2019
B
Station
BON−AR−S4
BON−W−S5
BON−W−S6
BON−AR−F1
VTR−W−F1
Feb 2018
Mar 2018
Apr 2018
May 2018
Jun 2018
Jul 2018
Aug 2018
Sep 2018
Oct 2018
Nov 2018
Dec 2018
Jan 2019

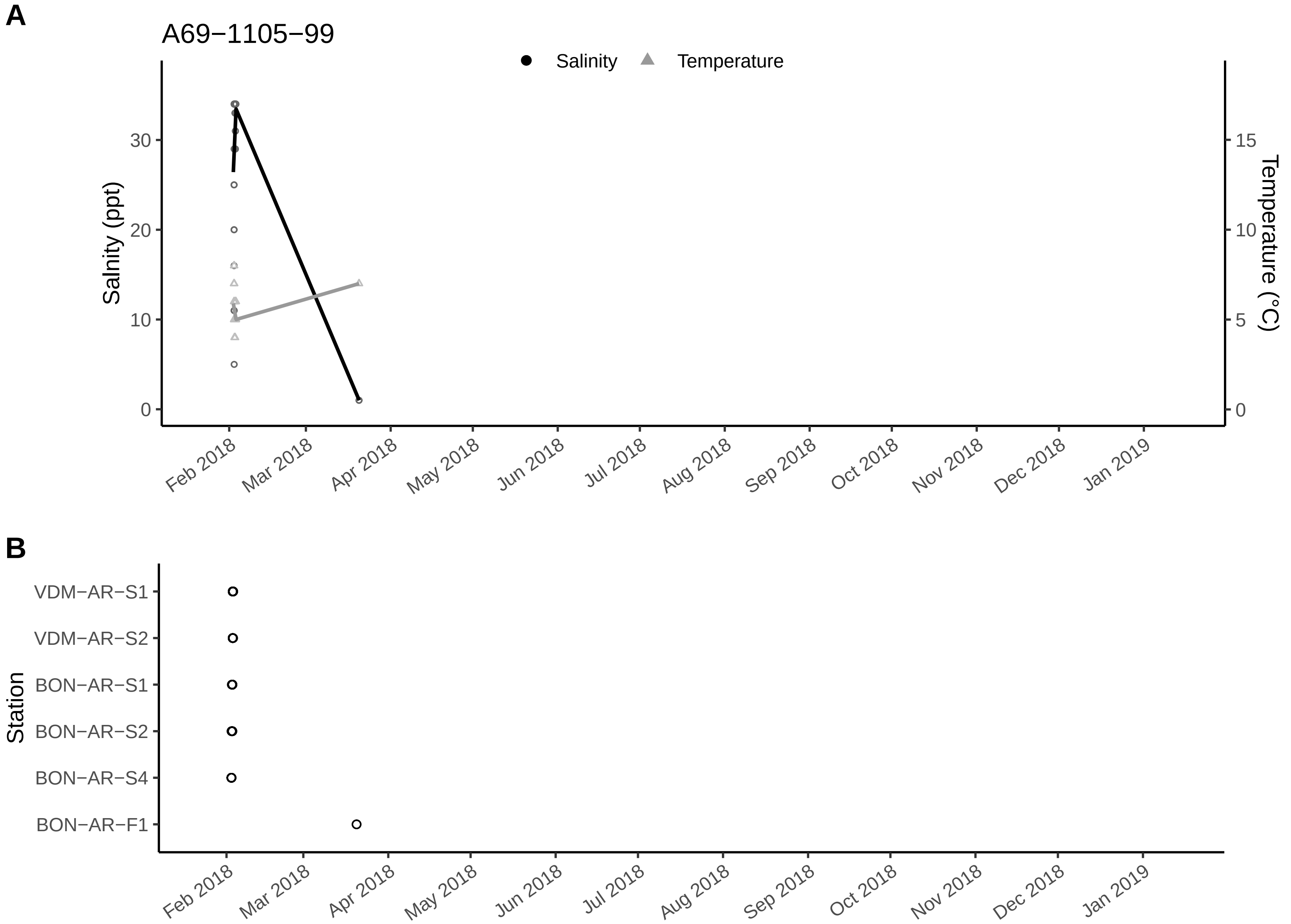
A
A69−1105−99
Salinity
Temperature
Salinity (ppt)
Temperature (°C)
0
10
20
30
0
5
10
15
Feb 2018
Mar 2018
Apr 2018
May 2018
Jun 2018
Jul 2018
Aug 2018
Sep 2018
Oct 2018
Nov 2018
Dec 2018
Jan 2019
B
Station
VDM−AR−S1
VDM−AR−S2
BON−AR−S1
BON−AR−S2
BON−AR−S4
BON−AR−F1
Feb 2018
Mar 2018
Apr 2018
May 2018
Jun 2018
Jul 2018
Aug 2018
Sep 2018
Oct 2018
Nov 2018
Dec 2018
Jan 2019

S1: Individual plots of A) salinity and water temperatures surrounding acoustically tagged brown trout during the tracking period. Solid lines: mean average. Open circles: single registrations. B) registrations at acoustic arrays deployed at sea and in freshwater. Legends on y-axis should be read: Bon: Lac Bontemps; VTR: Val Travers; VDM: Vallee des Merveilles; IRL: Baie Irlandaise; HUS: Passe Huske; AR/W: acoustic receiver model VR2-AR or VR2-W; -XX: receiver ID

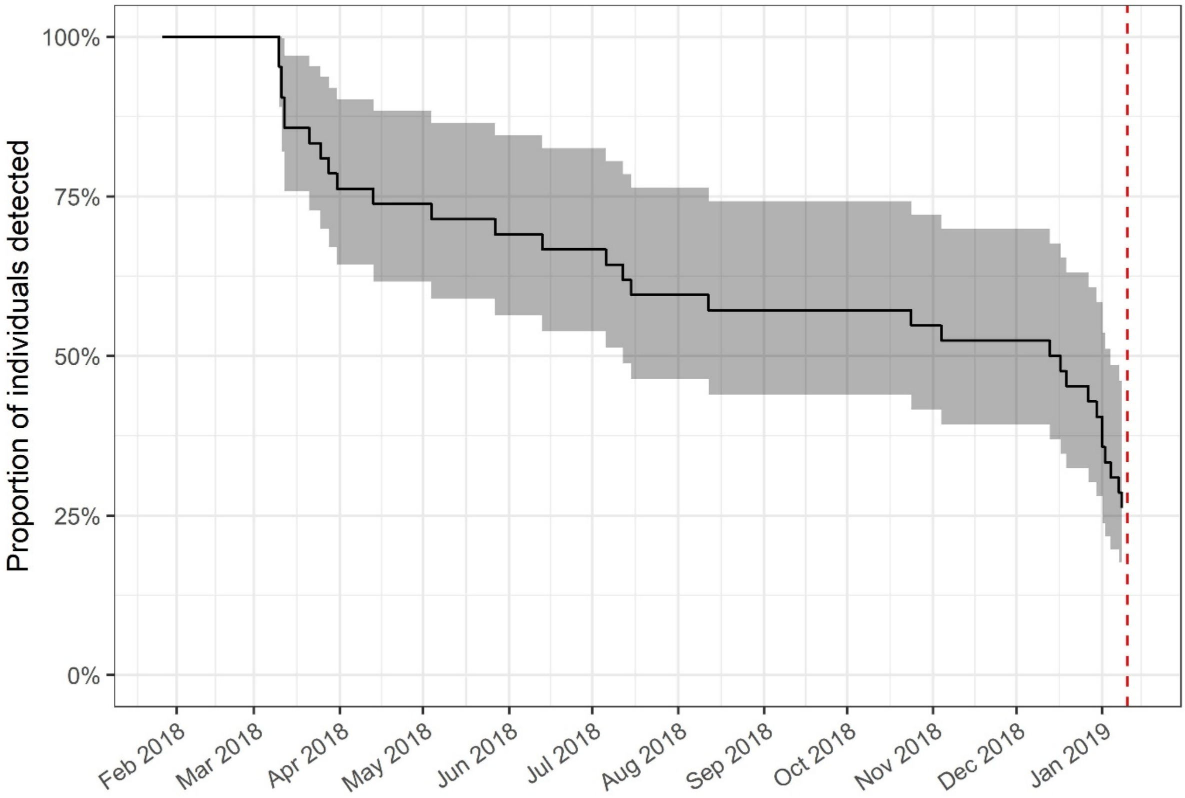

S2: Kaplan-Meir survival curves for acoustically tagged brown trout. The curve shows when fish were lost to the study through mortality, tag expulsion or predation followed by the predator excreting the tag.

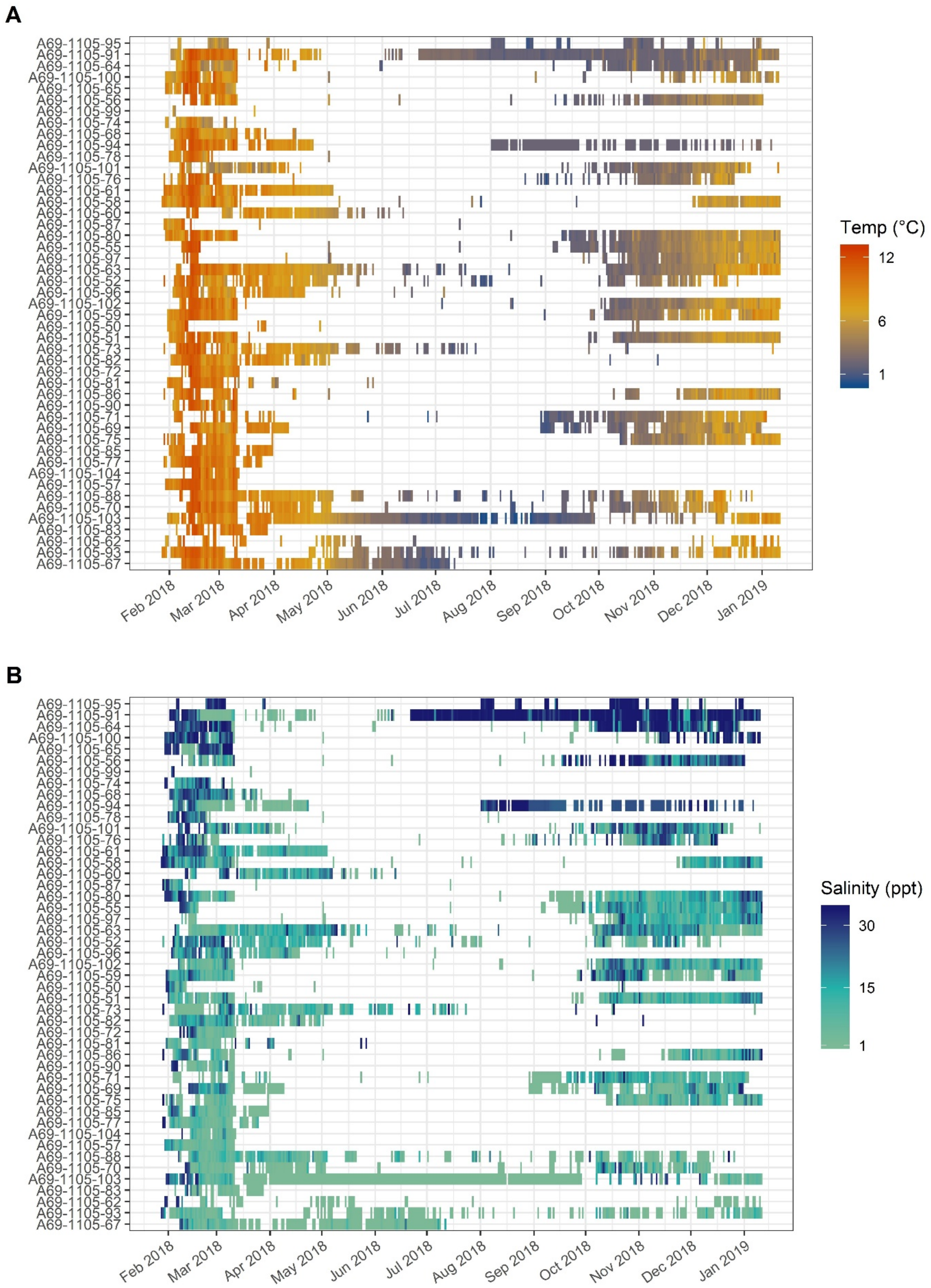

S3: Levels of water temperature (A) and salinity (B) surrounding acoustically tagged brown trout. Horisontal lines represent individual fish.
